# Pd_8_(PDip)_6_: Cubic, Unsaturated, Zerovalent

**DOI:** 10.1002/advs.202400699

**Published:** 2024-04-18

**Authors:** Kevin Breitwieser, Matteo Bevilacqua, Sneha Mullassery, Fabian Dankert, Bernd Morgenstern, Samuel Grandthyll, Frank Müller, Andrea Biffis, Christian Hering‐Junghans, Dominik Munz

**Affiliations:** ^1^ Coordination Chemistry Saarland University Campus C4.1 D‐66123 Saarbrücken Germany; ^2^ Dipartimento di Scienze Chimiche Università degli Studi di Padova via Marzolo 1 Padova I‐35131 Italy; ^3^ Experimental Physics and Center for Biophysics Saarland University Campus E2.9 D‐66123 Saarbrücken Germany; ^4^ Katalyse mit phosphorhaltigen Materialien Leibniz Institut für Katalyse e.V Albert‐Einstein‐Straße 29a D‐18059 Rostock Germany

**Keywords:** atomically precise, cluster, cube, phosphinidene, unsaturation

## Abstract

Atomically precise nanoclusters hold promise for supramolecular assembly and (opto)electronic‐ as well as magnetic materials. Herein, this work reports that treating palladium(0) precursors with a triphosphirane affords strongly colored Pd_8_(PDip)_6_ that is fully characterized by mass spectrometry, heteronuclear and Cross‐Polarization Magic‐Angle Spinning (CP‐MAS) NMR‐, infrared (IR), UV–vis, and X‐ray photoelectron (XP) spectroscopies, single‐crystal X‐Ray diffraction (sc‐XRD), mass spectrometry, and cyclovoltammetry (CV). This coordinatively unsaturated 104‐electron Pd(0) cluster features a cubic Pd_8_‐core, µ_4_‐capping phosphinidene ligands, and is air‐stable. Quantum chemical calculations provide insight to the cluster's electronic structure and suggest 5*s*/4*d* orbital mixing as well as minor Pd─P covalency. Trapping experiments reveal that cluster growth proceeds via insertion of Pd(0) into the triphosphirane. The unsaturated cluster senses ethylene and binds isocyanides, which triggers the rearrangement to a tetrahedral structure with a reduced frontier orbital energy gap. These experiments demonstrate facile cluster manipulation and highlight non‐destructive cluster rearrangement as is required for supramolecular assembly.

## Graphical abstract

An air‐stable Pd_8_ hexaphosphinidene nanocluster is reported. Spectroscopic investigations and calculations substantiate zerovalency, whereas the coordinative unsaturation allows for facile post‐functionalization.

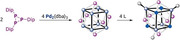

## 1 Introduction

**Figure 1:**
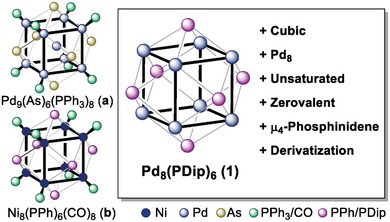
Previous work on coordinatively saturated Pd_9_‐ (a) and Ni_8_ (b) nanoclusters, and cluster **1** studied herein.

Cuboid structures are esthetically pleasing for their high symmetry. Symmetry is also important in self‐assembly to atomically precise nanoclusters.^[^ ^1^ ^]^ Such electronic confinement on the molecular level promises access to new materials for (opto‐)electronic devices, catalysts, magnets, and sensors.^[^ ^2^ ^]^ Hitherto, the field focused on clusters of the *p*‐block^[^ ^3^ ^]^ and heterometallic derivatives,^[^ ^4^ ^]^ bio‐mimetic manganese cubanes relevant for water‐splitting,^[^ ^5^ ^]^ iron‐sulfide cubanoids present in cytochromes and nitrogenases,^[^ ^6^ ^]^ as well as the coinage metals^[^ ^7^ ^]^ with a specific attention for gold. ^[^ ^8^ ^]^ Gold clusters are commonly synthesized through reduction of Au(I) or Au(III) precursors, and hence feature with rare exceptions^[^ ^9^ ^]^ an oxidation state >0 and coordinative saturation.^[^ ^9^ ^]^ Conversely, achieving coordinative unsaturation is desirable, as it provides a handle for controlled post‐functionalization, site‐differentiation, and the tuning of electronic properties.^[^ ^10^ ^]^ Atomically precise palladium clusters have so far attracted less attention than their gold counterparts. In fact, only a few examples beyond Pd_3_ are known,^[^ ^11^ ^]^ despite their emerging relevance for bond activation and electronic materials.^[^ ^12^ ^]^ Paralleling gold, most pallada‐clusters feature oxidation states >0, are obtained in low yields, and are coordinatively saturated. Whereas Pd_8_‐wires,^[^ ^13^ ^]^ nanosheets,^[^ ^14^ ^]^ and body‐centered [Pd_8_B(PPh_2_)_3_(PPh_3_)_2_(SXyl)_6_)]^[^ ^15^ ^]^ as well as [Pd_9_As_6_(PPh_3_)_8_]^[^ ^16^ ^]^ (**Figure** **1**a) are known, and notwithstanding computational prediction by Saillard and Halet,^[^ ^17^ ^]^ a Pd_8_‐cube remains elusive. Notably, compound **a** was obtained following earlier work on nickel by Dahl and Fenske, namely the isolation of cubic [Ni_8_(PPh)_6_(CO)_8_] (Figure 1b), [Ni_8_(PPh)_6_(PPh_3_)_4_], and [Ni_8_(P*i*Pr)_6_(PMe_3_)_6_].^[^ ^18^ ^]^ Palladium cluster **a** as well as the related Ni_8_‐compounds are stabilized by strongly coordinating and thus difficult‐to‐replace carbonyl or phosphine ligands. This is because they were synthesized, analogously to the common procedures for gold, in the presence of trapping ligands.

Herewith, we report the isolation, characterization, and derivatization of a cubic Pd_8_ cluster (**1**). Nanocluster **1** is obtained through the redox‐neutral reaction of Pd(0) precursors with a phosphorus(I) compound, namely a triphosphirane,^[^ ^19^ ^]^ in the absence of trapping ligands. Opposed to previous synthetic strategies, this approach provides high yields and access to coordinative unsaturation. Detailed spectroscopic investigations in combination with quantum‐chemical calculations reveal zero‐valency for the palladium‐atoms and 5*s*/4*d* mixing. Post‐synthetic derivatization is demonstrated through the reaction with an isocyanide.

## 2 Results and Discussion

**Scheme 1:**
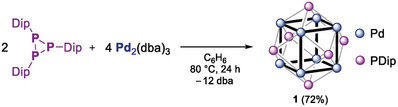
Synthesis of cluster **1** using low‐valent P(I)‐ and Pd(0) precursors.

Heating two equivalents of tris(dibenzylideneacetone)palladium, Pd_2_(dba)_3_, with one equivalent of the triphosphirane (PDip)_3_,^[^ ^19a^ ^]^ (Dip = 2,6‐*i*Pr_2_C_6_H_3_) in benzene to reflux for 1 day gave a mixture of two phosphorus‐containing products in an approximate ratio of 3:1 according to quantitative ^31^P‐ (Figure S6, Supporting Information) and ^1^H NMR spectroscopy (Figure S7, Supporting Information) using an internal standard. The ^31^P signal of the byproduct at *δ* = −102 ppm splits into a doublet (^1^ *J*_P‐H_ = 231.6 Hz) without ^1^H‐decoupling, indicating a PH moiety that likely derives from intramolecular CH insertion of the in situ formed phosphinidene PDip. The major product **1** (*δ* = +533 ppm) could be isolated analytically pure in 72% yield after workup (**Scheme** **1**). High‐resolution mass spectrometry (HRMS; Figure S11, Supporting Information) identified **1** as [Pd_8_(PDip)_6_].

**Figure 2:**
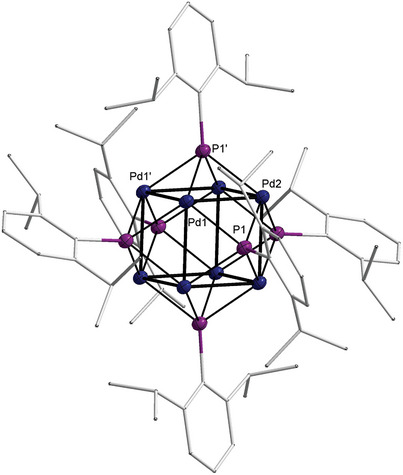
Molecular structure of **1** in the solid state. Dip‐substituents are given in wireframes, H‐atoms are omitted for clarity, thermal ellipsoids are displayed at 50% probability. Selected bond lengths (Å) and angles (°): Pd1–Pd2, 2.6997(6); Pd1–Pd1 2.6866(6); Pd1–P1 2.332(2); Pd1’–P1’ 2.341(2); Pd2–P1 2.358(2); P–C, 1.848(6); Pd1‐P1‐Pd2, 70.29(5).

Single‐crystals of **1** suitable for single‐crystal X‐Ray diffraction (sc‐XRD) were obtained from benzene solutions (**Figure** **2**). They confirmed a cubic Pd_8_‐cluster, which is capped by six µ_4_‐phosphinidene ligands (Figure 2). Compound **1** crystallized in the trigonal space group *R* $\bar{3}$ comprising a threefold axis of symmetry and an inversion center. The *S*_6_ symmetry in the solid‐state is due to the orientation of the Dip ligands (Figures S29 and S33, Supporting Information), and the Pd_8_ cube features almost equidistant Pd─Pd atoms with 2.6997(6) and 2.6866(6) Å. These values are consistent with body‐centered Pd_9_ cube **a** (2.71 and 2.68 Å), and slightly smaller than found in the face‐centered cubic structure of the metal (2.75 Å).^[^ ^20^ ^]^ Palladium µ^4^‐phosphinidenes remained hitherto unknown, yet the Pd─P bonds (2.332(2) to 2.358(2) Å) are elongated compared to the nickel complex **b** (2.20 and 2.22 Å).

Cross‐Polarized Magic Angle Spinning (CP‐MAS) ^31^P NMR spectroscopy^[^ ^21^ ^]^ of **1** confirmed axial symmetry (asymmetry parameter *η* = (*δ*_22_ − *δ*_33_)/*δ*_11_ = 0) also for the bulk material (Figure S8, Supporting Information). It revealed furthermore large anisotropy with *Ω* = *δ*_||_ − *δ*_⊥_ = 1124 ppm (*δ*_⊥_ = +160 ppm; *δ*_||_ = +1284 ppm; *δ*_iso_ = +535 ppm). DFT calculations (vide infra) of ^31^P NMR tensors (Figures S42 and S43, Supporting Information) are in line with a phosphinidene(I) electronic structure.

**Figure 3:**
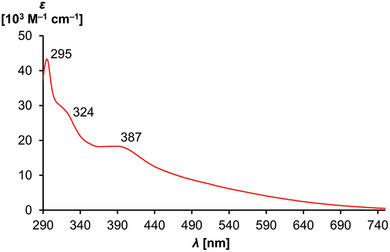
UV–vis electronic absorption spectrum of **1**.

Cluster **1** is dark red‐to‐brown and of exceptionally intense color. The UV–vis electronic absorption spectrum (**Figure** **3**) of single‐crystals shows maxima at 295 (*ε* = 47 100 m ^−1^ cm^−1^), 324 (*ε* = 27 500 m ^−1^ cm^−1^) and 387 nm (*ε* = 18 300 m ^−1^ cm^−1^). The mostly featureless and exceedingly broad spectrum with tailing up to 750 nm and a characteristic band between 370 and 390 nm is typical for palladium nanoparticles.^[^ ^22^ ^]^ TD‐DFT calculations suggest that the low‐energy transitions exhibit metal‐to‐ligand (Pd→P) charge transfer character (Figure S41, Supporting Information), which is consistent with an electron‐rich metal cluster, and phosphinidene character of the PDip‐substituents. Emission spectra did not reveal photoluminescence beyond a quantum efficiency of 1%.

X‐ray photoelectron spectroscopic (XPS) measurements yielded a signal for the palladium 3*d*_5/2_ level at 335.7 eV (Figure S35, Supporting Information). This value is slightly higher than for palladium foil (335.2 eV) and in the order of magnitude as found for palladium(0) complexes such as Pd(PPh_3_)_4_ and Pd_2_(dba)_3_ (335.6 and 336.4 eV, respectively).^[^ ^22^ ^]^ In contrast, palladium(I) (Pd_2_(OAc)_2_ (PPh_3_)_2_, 336.9 eV; Pd_2_Cl_2_(dppm), 337.1 eV) and especially palladium(II) complexes (PdBr_2_, 337.1 eV; PdCl_2_(PPh_3_)_2_, 337.8 eV; Pd(OAc)_2_, 338.7 eV) show higher binding energies (Table S2, Supporting Information).^[^ ^23^ ^]^ As for the phosphorus atoms’ P2*p* signals, binding energies of 130.2 eV were determined for **1**, and 130.4 eV for (PDip)_3_ (Figure S36, Supporting Information). These values are significantly lower than found for phosphorus(III) compounds (Table S3, Supporting Information) such as PdCl_2_(PPh_3_)_2_ (131.5 eV) and PPh_3_ (130.9 eV), yet similar to red phosphorus (130.0 eV) and even close to Ni_85_P_15_ (129.6 eV).^[^ ^23^ ^]^ In short, the XPS measurements confirm a redox‐neutral synthesis of **1** with physical oxidation states of 0 for the palladium and +I for the phosphorus atoms. Cyclic voltammetry (CV) measurements were performed to assess the potential for reversible redox chemistry. Although they suffered from the low solubility of **1**, a reversible wave was found at −2.13 V versus Fc/Fc^+^ (Figures S12 and S13, Supporting Information).

Opposed to coordinatively semi‐saturated [Ni_8_(PPh)_6_(PPh_3_)_4_], which has been described as “exceedingly air‐sensitive,” unsaturated **1** is thermally and chemically robust. It features a decomposition point of 291 °C, is air‐stable in the solid state, and persists^[^ ^24^ ^]^ in benzene‐solutions on moist air over days. Quantum‐chemical calculations at the r^2^SCAN‐3c and ZORA‐DF/def2‐TZVPP levels of theory (DF: PBE, TPSSh, PBE0) were conducted to elucidate the electronic structure of **1**. These calculations describe **1** as a zerovalent Pd_8_ phosphinidene cluster (Table S3, Supporting Information) with unusual 104 valence electrons (8 × 10, Pd; 6 × 4, PDip), which has been predicted though by Saillard and Halet to be in principle a favorable number of electrons.^[^ ^17b^ ^]^ The Electron Localization Function (*ELF*; Figures S44 and S45, Table S8, Supporting Information)^[^ ^25^ ^]^ indicates a high degree of electron delocalization within the Pd_8_ cube and thus parallels in principle elemental metals (*ELF* value *η* = 0.2 for the Pd─Pd bond critical point (BCP)), but the maxima are localized exclusively at the metal sites. This suggests, in combination with the significant Pd─ligand interaction (*η* = 0.4; Pd─P BCP), that the µ_4_‐coordinating phosphinidene ligands are crucial for the cluster's stability.

**Figure 4:**
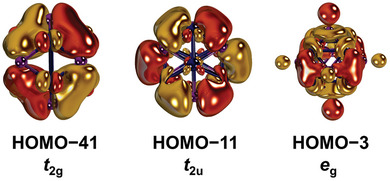
Selected canonical orbitals (PBE), Dip‐groups truncated with H‐atoms. See Figure S38, Supporting Information for the entire MO‐diagram.

Also, the shapes of pertinent canonical Kohn‐Sham molecular orbitals (MOs, **Figure** **4**; see Figure S38, Supporting Information for the complete MO diagram) such as the HOMO−41 (*t*_2g_), HOMO−11 (*t*_2u_) and HOMO−3 (*e*_g_) support efficient electron delocalization. The Pd─P covalency and the 5*s* character in these orbitals is substantial.^[^ ^26^ ^]^ This is illustrated by the HOMO−41, where two Pd‐atoms feature *c*(5*s*) = 0.35, and where the Pd:P contribution amounts to 3:1 (Löwdin's Population Analysis). The latter mixing is in line with the XPS measurements (vide supra), which substantiate a marginally higher physical oxidation state than for Pd‐metal.

**Figure 5:**
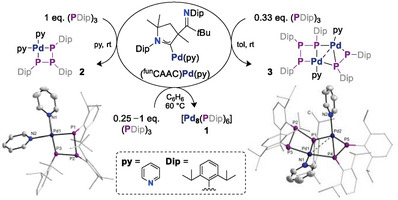
Reactivity of (^fun^CAAC)Pd(py) towards (PDip)_3_ and molecular structures of **2** (left) and **3** (right) in the solid state with Dip‐groups rendered as wireframes, H‐atoms and disordered parts omitted for clarity. See Figures S30 and S31, Supporting Information for further details.

Cluster **1** also forms when replacing Pd_2_(dba)_3_ by an exceedingly reactive palladium(0) precursor, namely an imino‐functionalized cyclic(alkyl)(amino)carbene (^fun^CAAC)^[^ ^27^ ^]^ supported complex (^fun^CAAC)Pd(py).^[^ ^28^ ^]^ This ligand with a hemilabile imino‐group supports terminal palladium nitrenes,^[^ ^29^ ^]^ which are isoelectronic with phosphinidenes. Treating (^fun^CAAC)Pd(py) with 0.25, 0.33 or 1 equivalents of (PDip)_3_ in perdeutero‐benzene led to the instantaneous release of the free ^fun^CAAC and quantitative conversion to various intermediates, which converted at 60 °C to **1** (**Figure** **5**; Figure S14, Supporting Information). The dissociation of the ^fun^CAAC, which is only metastable in free form,^[^ ^27b^ ^]^ is likely due to steric effects.^[^ ^30^ ^]^ When running the reaction at room temperature in pyridine, single crystals suitable for SC‐XRD analysis formed in situ and identified compound **2** [(PDip)_3_Pd(py)_2_] (Figure 5, left) as pyridine‐trapped intermediate *en route* to **1**. The same is true for single crystals of **3** [(PDip)_5_Pd_2_(py)_2_] (Figure 5, right), which formed in situ at room temperature in toluene.

We conclude that, instead of phosphinidene transfer, the palladium(0) precursor initially inserts into one P─P bond of (PDip)_3_, which is followed by dissociation of the CAAC to give **2**. The subsequent cluster growth to **3** presumably^[^ ^31^ ^]^ involves the association of two monomers of **2** under formal elimination of PDip. Intrigued by the coordinative unsaturation of **1**, we tested its stability in the presence of common reagents in organometallic chemistry. Exposure to dihydrogen in an in situ NMR experiment with heating to 60 °C did not lead to signs of decomposition, which further highlights the robustness of **1**. In the presence of ethylene, a weak interaction was evidenced by a shift of the signal in the ^31^P NMR spectrum from 533 to 537 ppm (Figure S24, Supporting Information) and slightly shifted cluster signals in the ^1^H NMR spectroscopic analysis (Figure S25, Supporting Information). Corroborating the weak interaction, ethylene is removed quantitatively *in vacuo*, thereby cleanly regenerating **1**.

**Figure 6:**
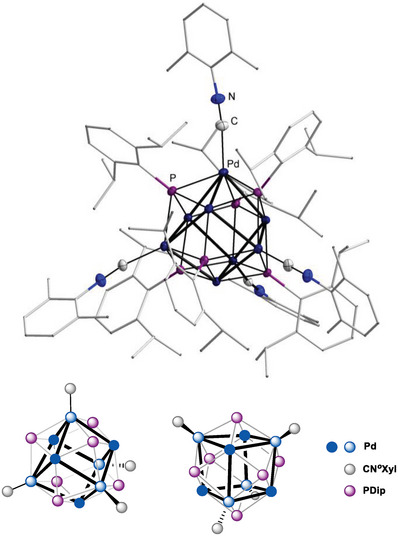
Molecular structure of **4** in the solid state (top). Dip‐ and *o*Xyl substituents are given in wireframes, H‐atoms and co‐crystallized solvent molecules are omitted for clarity, thermal ellipsoids are displayed at 50% probability, structural parameters are given in Figure S32, Supporting Information. Illustration of the cluster's polyhedron from two different angles (bottom; *c.f*. Figure S33, Supporting Information).

Probing the potential for material chemistry and supramolecular assembly, cluster **1** was treated with *ortho*‐xylyl isocyanide (CN*o*Xyl). Indeed, sequential decoration by either two‐, three‐, or four (Figures S26 and S27, Supporting Information) isocyanide ligands was achieved. Tetracoordinate [Pd_8_(PDip)_6_(CN*o*Xyl)_4_] **4** was isolated analytically pure. Vapor diffusion of pentane into a saturated solution in benzene afforded single crystals suitable for sc‐XRD experiments, which contained two molecules of co‐crystallized benzene and one molecule of pentane. The isocyanide ligands distort the Pd_8_‐cube toward a triakistetrahedron by pulling out four palladium atoms, whereupon the other four palladium atoms approach each other. The coordination geometry may then be understood as a pair of two dual Pd_4_ tetrahedra, whereof the corners of the larger one (4.20 Å versus 3.46 Å edge length) are coordinated by the isocyanides (**Figure** **6**, bottom). Both the closest Pd─Pd (average: 2.74 Å) and Pd─P (average: 2.36 Å) distances (Figure S32, Supporting Information) increase slightly with respect to nanocluster **1**. In line with the elongated bonds, the *ELF* analysis suggests enhanced electron delocalization within the Pd_8_ cube (Figures S46 and S47, Table S8, Supporting Information). The *T*_2_ C─N stretching vibration of the isocyanide ligands appears at 2078 cm^−1^ in the infrared (IR) spectrum (Figure S22, Supporting Information), which is consistent with literature values for palladium(0), but not with palladium(II) complexes, where more than 2200 cm^−1^ are expected.^[^ ^32^ ^]^ As anticipated, **4** absorbs light even stronger than **1**, and the maxima in the UV–vis spectrum (Figure S23, Supporting Information) are redshifted to 297 (*ε* = 173 500 m ^−1^ cm^−1^) and 431 nm (*ε* = 88 800 m ^−1^ cm^−1^), with tailing beyond 760 nm. The isocyanide ligands lower the optical HOMO–LUMO energy gap by about 0.33 eV, thus demonstrating that the decoration of the vacant metal‐sites allows not only for derivatization, yet also the modification of the cluster's electronic properties. Eventually, we explored the reaction of Pd_2_(dba)_3_ with (PDip)_3_ in the presence of xylyl isocyanide, but found that **4** was obtained only in traces (Figure S21, Supporting Information). This observation further highlights the synthetic value of coordinatively unsaturated **1**.

## 3 Conclusion

In summary, treating palladium(0) precursor Pd_2_(dba)_3_ with the triphosphirane (PDip)_3_ affords the nanocluster [Pd_8_(PDip)_6_] (**1**) in 72% yield. The cubic Pd_8_ core is thereby stabilized by µ^4^‐capping phosphinidene ligands. Intermediates **2** and **3** *en route* to **1** were trapped as pyridine adducts and provide insights into the cluster growth mechanism. Nanocluster **1** is coordinatively unsaturated, as its synthesis proceeds in the absence of trapping agents, and features an unusual valence electron count of 104. It is strongly colored, and the broad electronic absorption spectrum parallels the ones of palladium nanoparticles, as it extends to the near infrared region and features a band ≈390 nm. Despite of its zerovalent and low‐coordinate nature, yet validating predictions by Saillard and Halet,^[^ ^17b^ ^]^ nanocluster **1** is thermally stable up to almost 300 °C, and chemically robust in the presence of moisture and dioxygen. Cluster **1** weakly coordinates ethylene and binds isocyanides. Isocyanide binding lowers the HOMO–LUMO energy gap more than 0.3 eV and triggers the structural rearrangement to tetrahedral [Pd_8_(PDip)_6_ (CN*^o^* Xyl)_4_] (**4**), which cannot be obtained directly from the precursor molecules.

## Conflict of Interest

The authors declare no conflict of interest.

## Supporting information

Supporting Information

Supporting Information

## Data Availability

The data that support the findings of this study are available in the supplementary material of this article.
