# Supplementary material for: Pd8(PDip)6: Cubic, Unsaturated, Zerovalent
Source: Adv Sci (Weinh). 2024 Apr 18;11(25):2400699. doi: 10.1002/advs.202400699 (PMC11220702; doi:10.1002/advs.202400699)
Supplement: Supplementary file 1 — Supporting Information [file ADVS-11-2400699-s002.pdf]

ADVANCED SCIENCE
Open Access

Supporting Information

for *Adv. Sci.*, DOI 10.1002/advs.202400699

$Pd_8(PDip)_6$: Cubic, Unsaturated, Zerovalent

Kevin Breitwieser, Matteo Bevilacqua, Sneha Mullassery, Fabian Dankert, Bernd Morgenstern, Samuel Grandthyll, Frank Müller, Andrea Biffis, Christian Hering-Junghans and Dominik Munz**

Supporting Information

$Pd_8(PDip)_6$: Cubic, Unsaturated, Zerovalent

Kevin Breitwieser, Matteo Bevilacqua, Sneha Mullassery, Fabian Dankert, Bernd Morgenstern, Samuel Grandthyll, Frank Müller, Andrea Biffis, Christian Hering-Junghans, Dominik Munz**

Table of Contents

1. Experimental Procedures: General S2
2. Synthesis and Characterization of **1** [$Pd_8(PDip)_6$] and Intermediates S3
   2.1. Synthesis of **1** from $Pd_2(dba)_3$ and Characterization S3
   2.2. Synthesis of **1** from (^{fun}CAAC)Pd(py) S10
   2.3. Crystallization of Trapped Intermediates **2** and **3** S11
   2.4. Stability of **1** in Aerated Benzene S11
3. Synthesis and Characterization of **4** [$Pd_8(PDip)_6(CN\textit{o}Xyl)_4$] S13
4. Interaction of **1** with Ethylene S17
5. Titration of **1** with of 2,3-Dimethylphenylisocyanide S18
6. Single Crystal Structure Elucidation and Refinement (SC-XRD) S20
7. X-Ray Photoelectron Spectroscopy (XPS) S26
8. Computational Details S30
9. References S52
10. Author Contributions S52

1. Experimental Procedures: General

All work was conducted under an atmosphere of dinitrogen, either using Schlenk-line techniques or an MBraun glovebox. Solvents were dried using a two-column solid-state purification system MBraun SPS 5/7 (MN Kieselgel 60, <0.08 mm), degassed *via* freeze-pump-thaw, and transferred to the glovebox without exposure to air. *Ortho*-difluorobenzene was dried by distillation from calcium-hydride. Benzene, pentane and hexane were stored over a mirror of potassium, tetrahydrofurane, *ortho*-difluorobenzene and diethylether were stored over activated 4Å molecular sieves. Celite was dried by heating to 200 °C *in vacuo* for 48 h. NMR solvents (Tol-d_8, C_6D_6) were obtained dry and packaged under argon and stored over a mirror of potassium. Pyridine-d_8 was obtained dry and packaged under argon and stored over molecular sieves. $(PDip)_3$[1] and (^{fun}CAAC)Pd(py)[2] were prepared according to the literature. All other reagents were obtained from commercial sources and used as is without further purification.

^{1}H, ^{31}P and ^{13}C NMR spectra were recorded on a Bruker Advance III HD 400 instrument operating at 400 MHz for ^{1}H, 162 MHz for ^{31}P and at 101 MHz for ^{13}C, respectively, at a probe temperature of 25 °C. The chemical shifts δ are calculated in ppm; the solvent residual signals of incompletely deuterated solvent molecules were used as internal reference for the ^{1}H NMR spectra, and the respective carbon solvent signals for the ^{13}C NMR spectra. NMR multiplicities are abbreviated as follows: s = singlet, d = doublet, t = triplet, spt = septet, m = multiplet, br = broad signal. Coupling constants *J* are given in Hz.

Solid state CP-MAS NMR spectra were acquired on a Bruker AV400 WB spectrometer with approximately 150 mg of sample. Chemical shifts given in the main part of the manuscript relate to the averaged values.

UV-Vis spectra were recorded on a Shimadzu UV-2600 spectrometer in quartz cells with a path length of 1 cm.

Elemental analyses were carried out on an elementar vario Micro Cube.

Melting points were determined in capillary tubes with an Electrothermal IA 9100 Melting Point Apparatus.

HRMS-APPI spectra were recorded on a Quadrupole Linear Ion Trap (QqLIT), AB Sciex API 5500 QTRAP.

Cyclovoltammetry measurements of **1** were recorded with an EmStat^{3+} Blue potentiostat by PalmSens in a nitrogen filled glovebox, using a glassy carbon working electrode, platinum counter electrode and non-aqueous Ag/Ag^{+} reference electrode as well as NBu_4PF_6 (0.2 M) as electrolyte. After the initial measurements, ferrocene was added as internal standard and the voltammograms were corrected by positioning the redox potential of ferrocene to 0 V. Compound **1** proved not soluble enough for CV in THF, and also CH_2Cl_2 and MeCN proved unsuitable. The CV was hence recorded in a saturated solution (*ca.* 10 mg of **1**) of *ortho*-difluorobenzene (4 mL), where **1** proved at least moderately soluble.

FTIR spectra for solid samples were recorded in nujol mulls on attenuated total reflectance (ATR) using a Vertex 70 spectrometer from Bruker (Karlsruhe, Germany). The spectra were obtained with an average of 16 scans in the wavelength range from 550 cm^{-1} to 3200 cm^{-1} at a resolution of 2 cm^{-1}. All IR spectra were normalized to maximum intensity 1 and the intensities are reported as very strong (vs), strong (s), medium (m), weak (w), very weak (vw).

2. Synthesis and Characterization of 1 [$Pd_8(PDip)_6$] and Intermediates

2.1. Synthesis of 1 from $Pd_2(dba)_3$ and Characterization

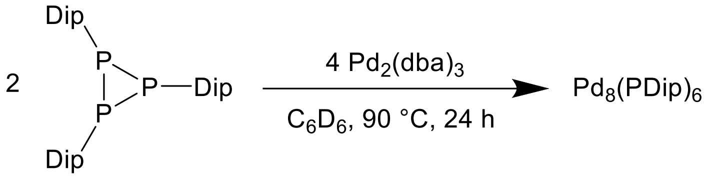

In a nitrogen-filled glovebox, $Pd_2(dba)_3$ (211 mg, 0.230 mmol, 4 equiv.) and 1,2,3-tris[2,6-bis(isopro-pyl)phenyl]triphosphirane (66 mg, 0.115 mmol, 2 equiv.) were mixed in a thick-walled Schlenk flask with vacuum valve, and benzene (6 mL) was added. The resultant dark solution was stirred outside the glovebox at 90 °C for 24 h. Upon cooling to room temperature, a fine precipitate started to form. The immediate and quantitative (D1 = 30 s) ^{31}P spectroscopic analysis of the supernatant solution at this stage indicated a mixture of two phosphorus-containing compounds with a phosphorus ratio of 3:1 (Fig. S6), thus suggesting a crude yield higher than 75%. Also the ^{1}H-NMR spectroscopic analysis, referenced *versus* the internal standard hexamethylbenzene, suggested >65% yield in solution (Fig. S7). The suspension was then concentrated to about 2 mL *in vacuo* and quantitative precipitation was induced by adding 20 mL of hexanes. The suspension was filtered over celite, thereby depositing the precipitates on top, and washed with hexanes (20 mL). The solids were then extracted with 20 mL of boiling toluene until the celite turned colourless. The resultant dark solution was concentrated *in vacuo* to *ca.* 5 mL and stored in the freezer for 16 h, resulting in the formation of dark reddish-to-black crystals. The crystalline material was collected, washed with diethyl ether (15 mL) and dried *in vacuo* to afford the dark-reddish-brown product in a yield of 72% (84 mg, 0.042 mmol). The product may be recrystallized from hot benzene, affording single-crystals suitable for X-ray diffraction.

^{1}H NMR (400 MHz, C_6D_6): δ = 1.20 (d, 72H, ^{3}J = 6.72 Hz), 5.11 (q, 12H, ^{3}J = 6.61 Hz), 7.12 (d, 12H, ^{3}J = 7.65 Hz), 7.26 (t, 6H, J = 7.70 Hz) ppm. Once precipitated, cluster **1** is essentially insoluble in C_6D_6. Hence, it was heated to 80 °C to over-saturate the solution prior to acquisition.

^{31}P NMR (162 MHz, C_6D_6): δ = 533.37 (s) ppm. Once precipitated, cluster **1** is essentially insoluble in C_6D_6. Hence, it was heated to 80 °C to over-saturate the solution prior to acquisition.

^{13}C NMR (100 MHz, C_6D_6): δ = 24.49 (*i*Pr; CH_3), 32.71 (*i*Pr; CH), 123.27 (CH^m), 131.61 (CH^p), 141.81 (CH^o), 146.71 (CP) ppm. Once precipitated, cluster **1** is essentially insoluble in C_6D_6. Hence, it was heated to 80 °C to over-saturate the solution prior to acquisition.

Melting point: 291 °C (decomposition).

UV-Vis (C_6H_6, 1.66 × 10^{-5} M): 295 nm (47,100 M^{-1} cm^{-1}), 324 nm (27,500 M^{-1} cm^{-1}), 388 nm (18,300 M^{-1} cm^{-1}).

IR (nujol): $\tilde{\nu}$ = 2943 cm^{-1} (w), 1446 cm^{-1} (w).

APPI-HRMS, [$Pd_8(PC_{12}H_{17})_6^{+}$]: Calculated 2003.87 m/z, observed 2004.02 m/z. No pertinent signal was obtained in ESI mode.

CHNS Analysis: Calcd for $C_{72}H_{102}P_6Pd_8$: C, 43.14; H, 5.13; N, 0.00; S, 0.00. Found: C, 42.90; H, 4.88; N, 0.00; S, 0.00.

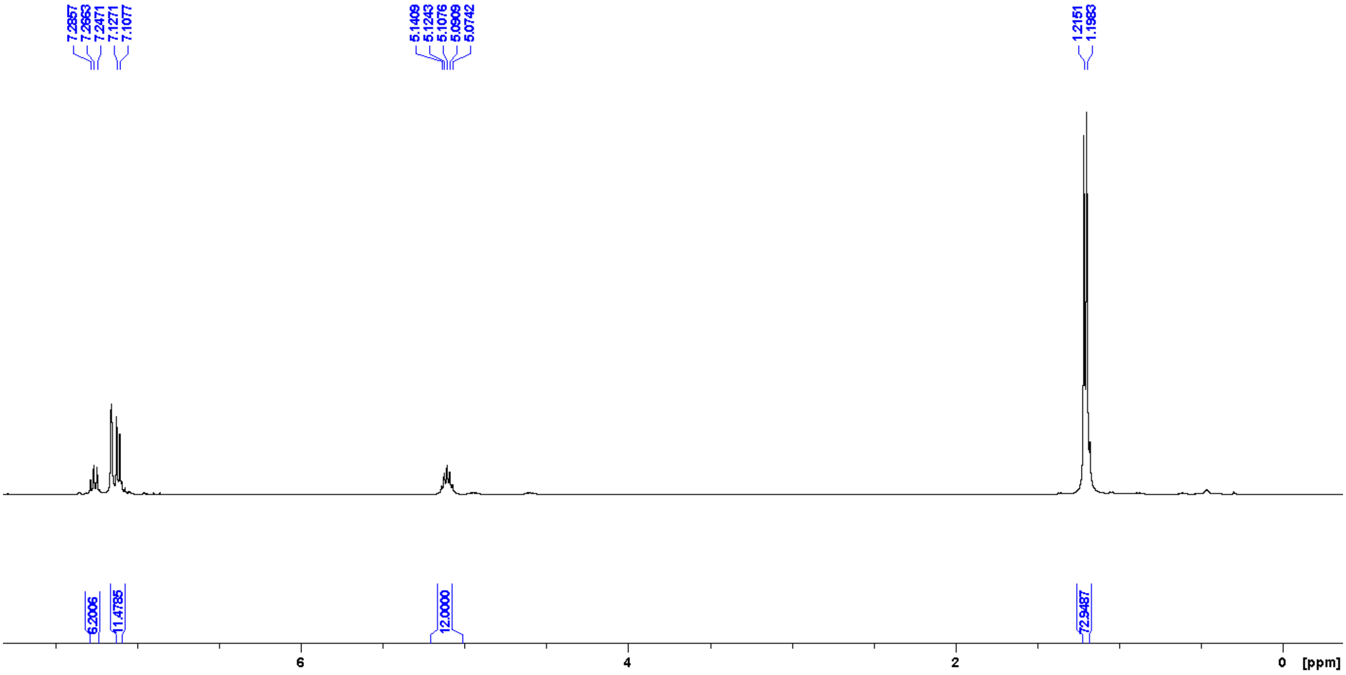

Figure S1. ^{1}H NMR (400 MHz, C_6D_6) spectrum of **1**.

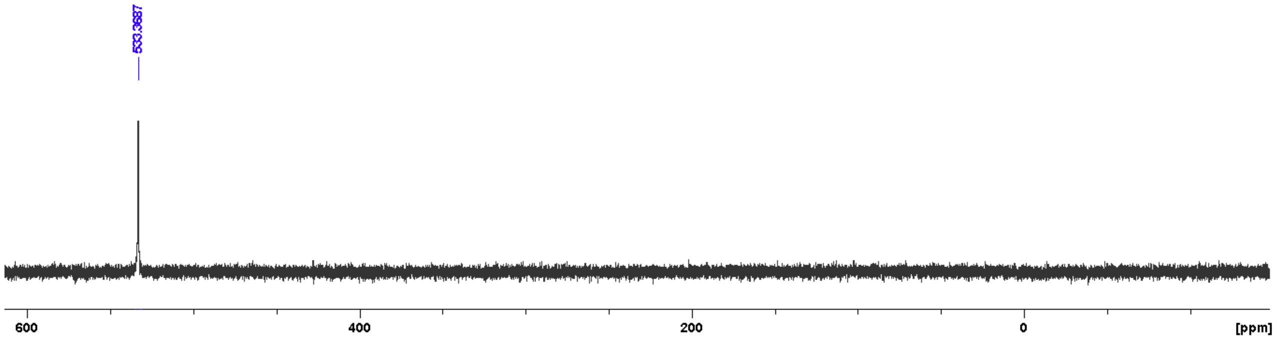

Figure S2. ^{31}P{^{1}H} NMR (162 MHz, C_6D_6) spectrum of **1**.

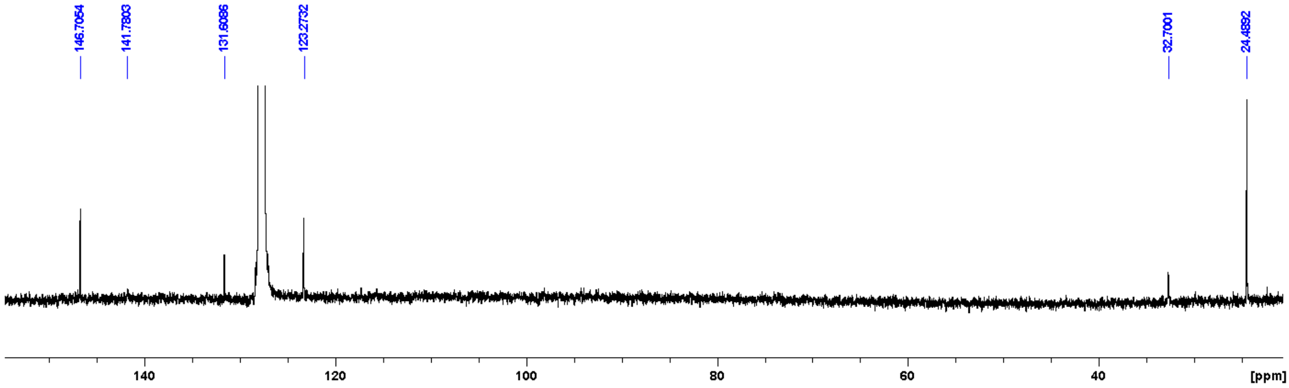

Figure S3. ^{13}C{^{1}H} NMR (101 MHz, C_6D_6) spectrum of **1**.

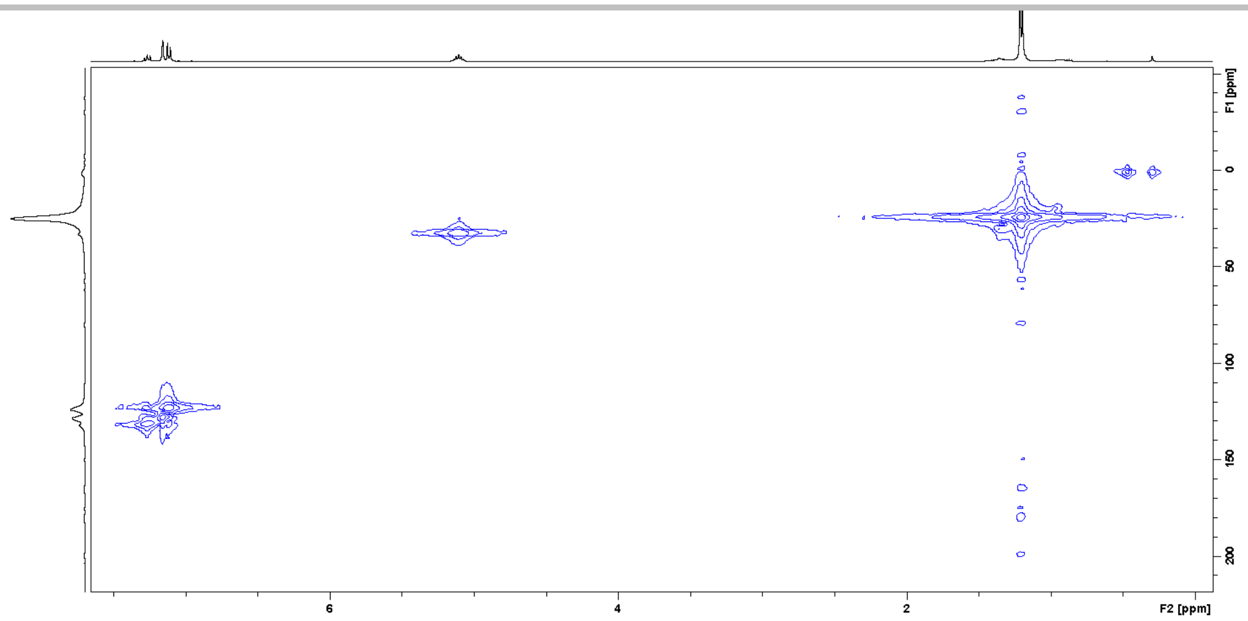

Figure S4. HMQC(^{1}H,^{13}C) NMR (C_6D_6) spectrum of **1**.

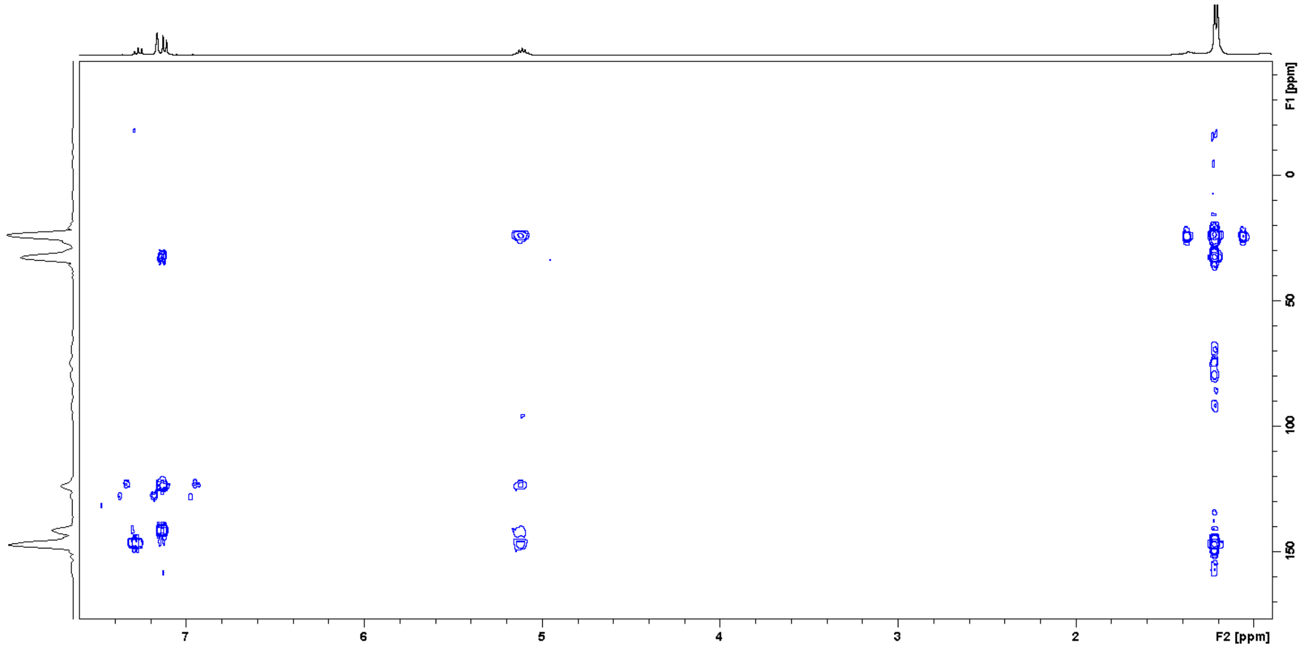

Figure S5. HMBC(^{1}H,^{13}C) NMR (C_6D_6) spectrum of **1**.

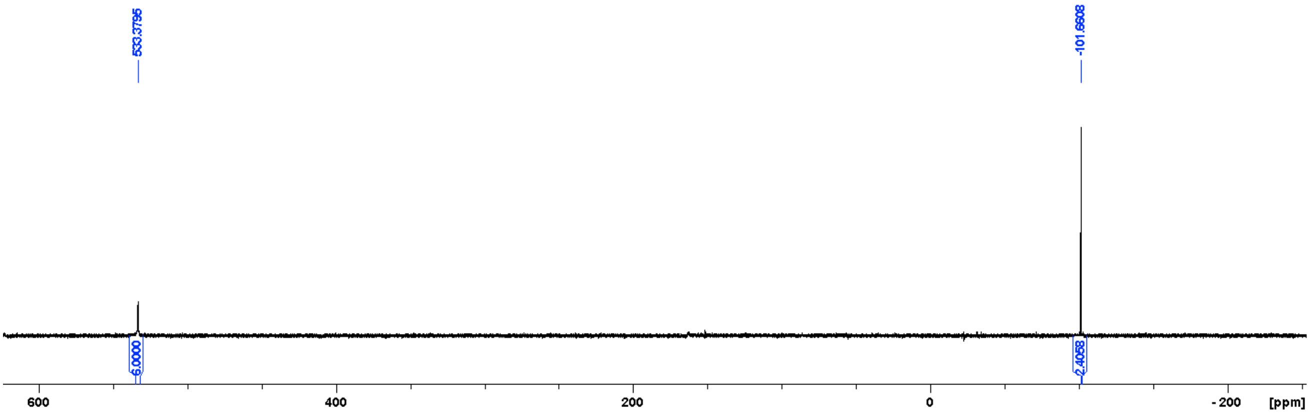

Figure S6. $^{31}P\{^{1}H\}$ NMR (162 MHz, C_6D_6) spectrum of the crude reaction mixture after heating to 90 °C for 24 h and cooling to room temperature.

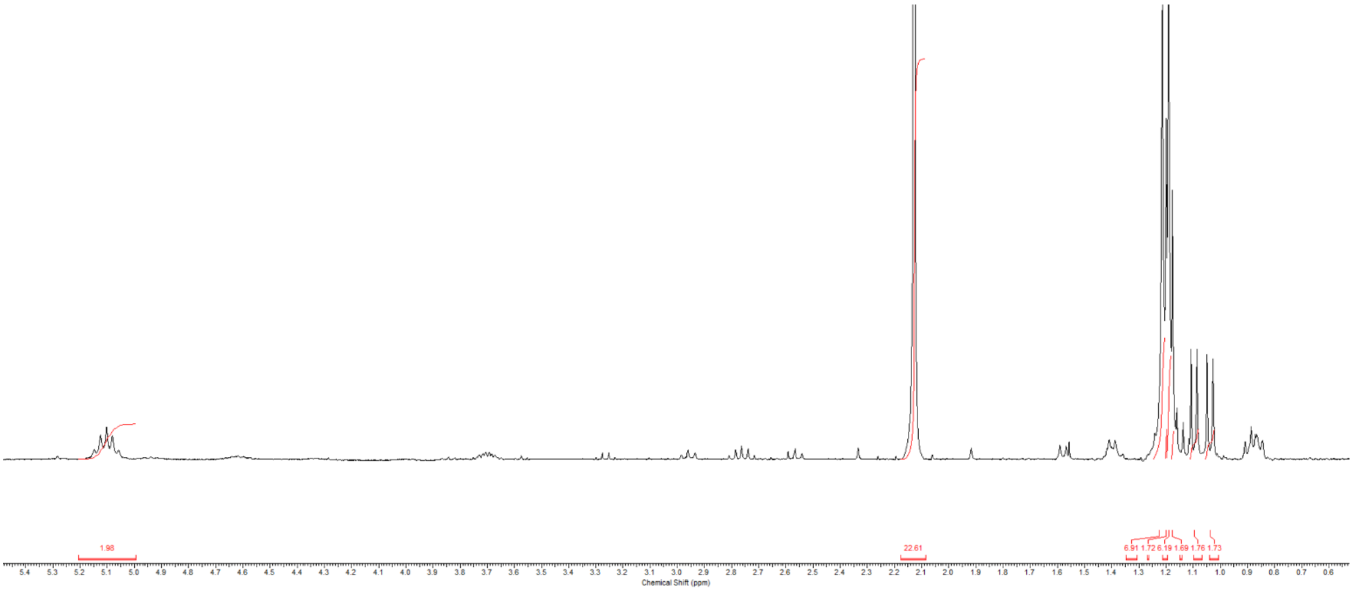

Figure S7. Pertinent area between 0.5 and 5.5 ppm of the ^{1}H NMR (400 MHz, C_6D_6) spectrum of the crude mixture after heating to 90 °C for 24 h and cooling to room temperature in the presence of the internal standard C_6Me_6 (2.13 ppm). Automatic baseline correction was applied to allow for approximate integration in the area of 1.0 – 1.3 ppm. Additionally, a ^{1}H NMR spectrum of $(PDip)_3$ had been recorded prior to addition of $Pd_2(dba)_3$ for quantification/reference in respect to the standard.

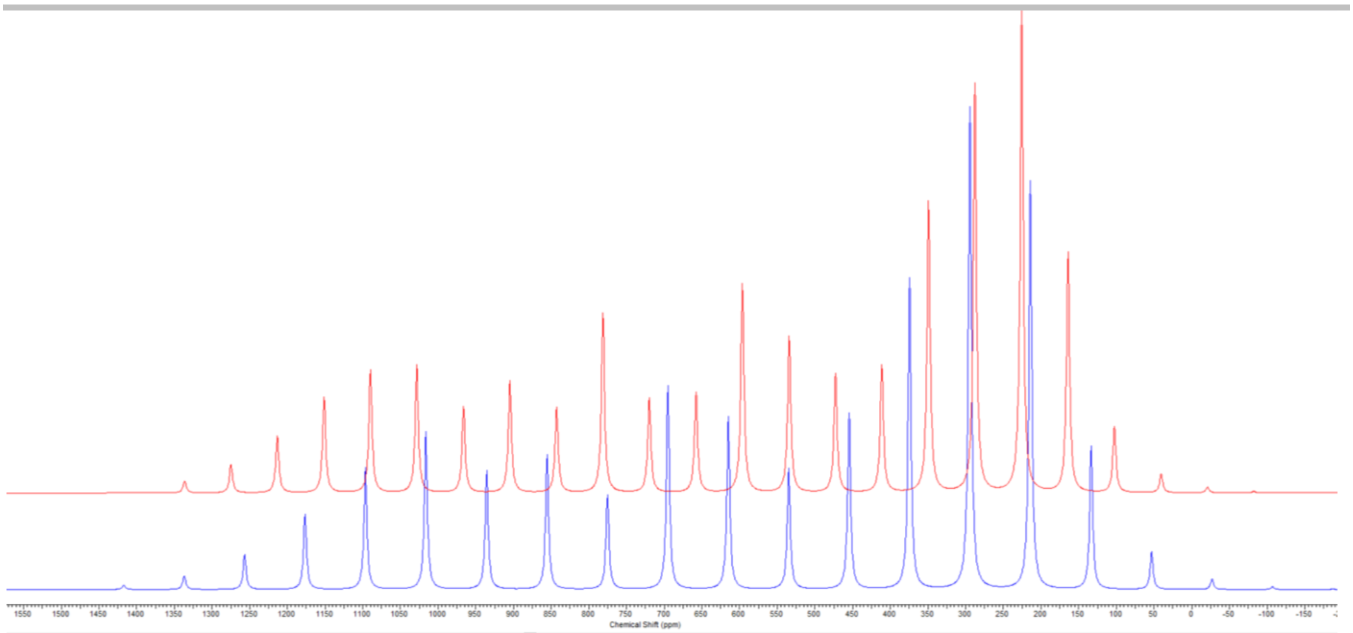

Figure S8. CP-MAS ^{31}P NMR spectrum of **1** as obtained with spinning frequencies of 10 MHz (blue, bottom; δ_{11} = 1272.4 ppm; δ_{22} = 166.5 ppm; δ_{33} = 165.3 ppm) and 13 MHz (red, top); δ_{11} = 1295.6 ppm; δ_{22} = 154.8 ppm; δ_{33} = 154.8 ppm. Values given in the main part of the manuscript relate to the averaged values.

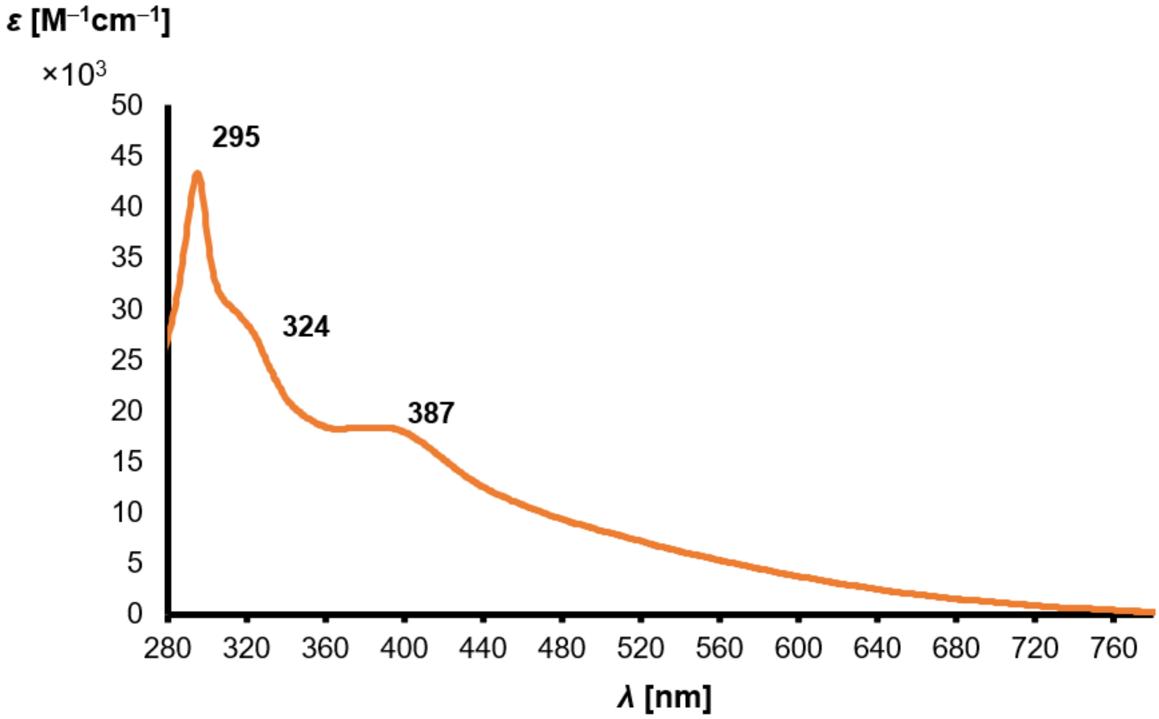

Figure S9. UV-Vis electronic absorption spectrum of **1** dissolved in C_6H_6, concentration = 1.7×10^{-5} M.

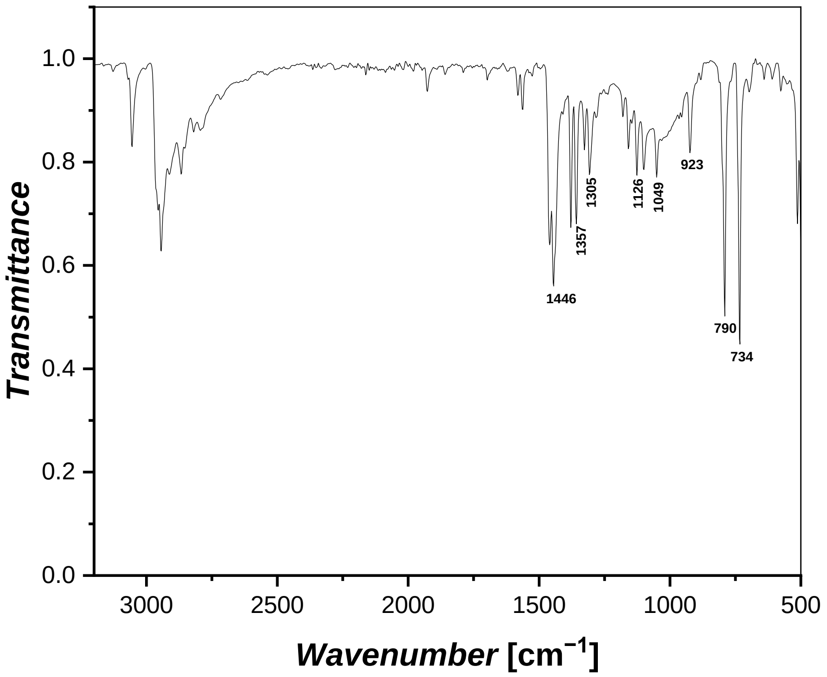

Figure S10. IR spectrum of **1**.

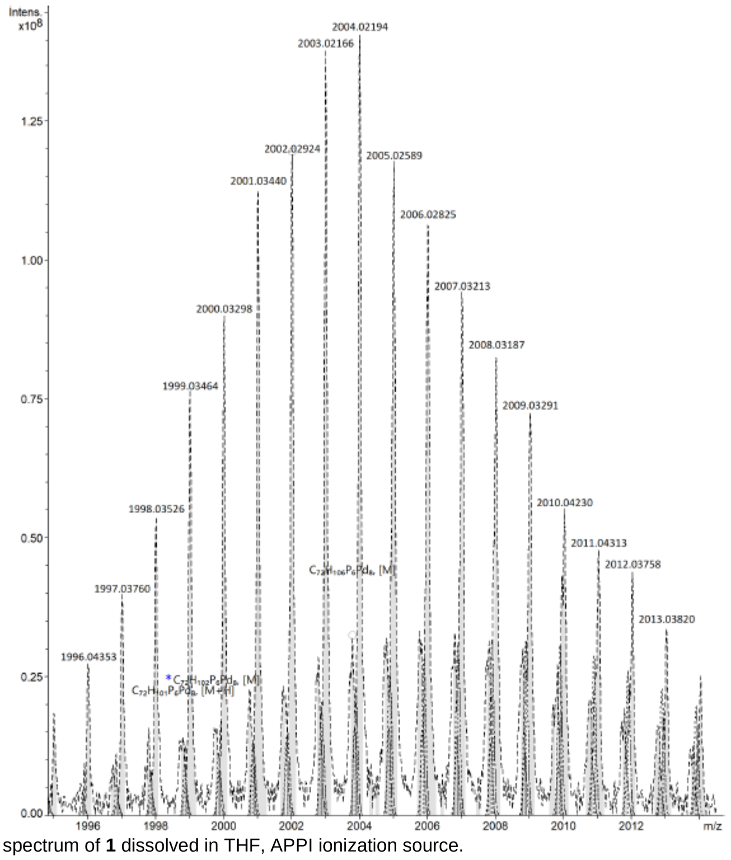

Figure S11. High-resolution mass spectrum of **1** dissolved in THF, APPI ionization source.

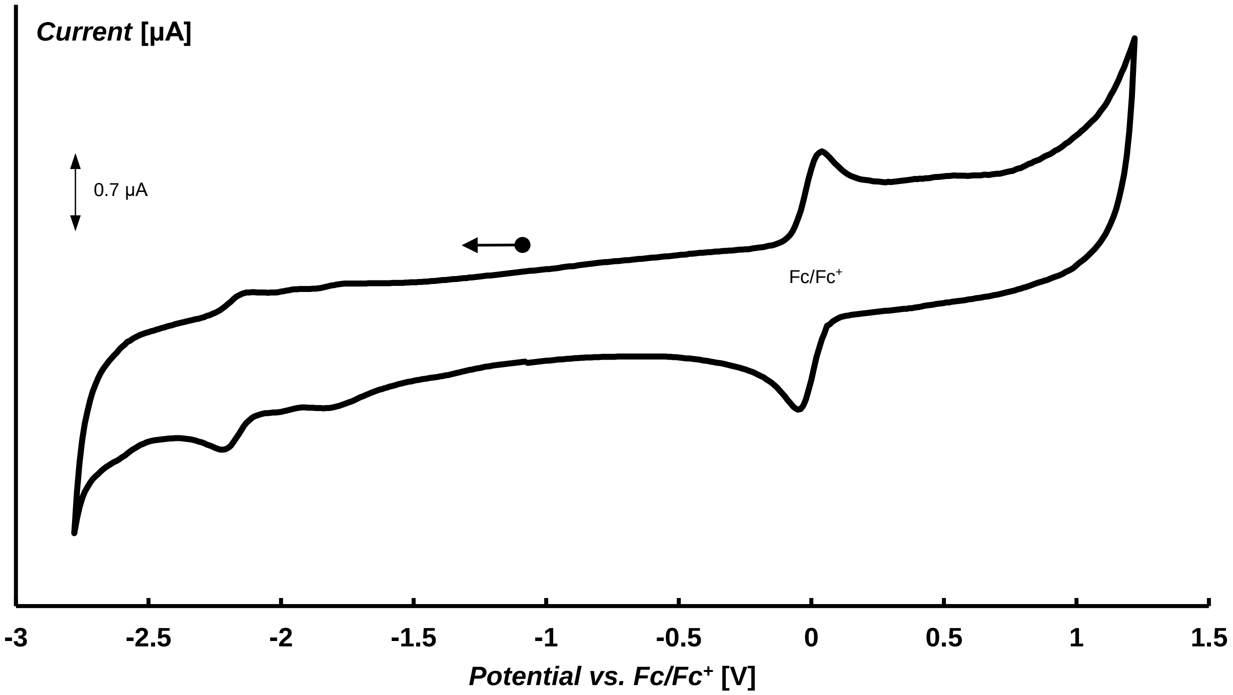

Figure S12. Cyclic voltammogram for **1**, measured at 100 mV s^{-1} in 0.2 M NBu_4PF_6 *ortho*-difluorobenzene solution, in the presence of the internal ferrocene standard (second scan of two is shown). Open-Circuit Potential (OCP; in the absence of ferrocene): −0.05 V).

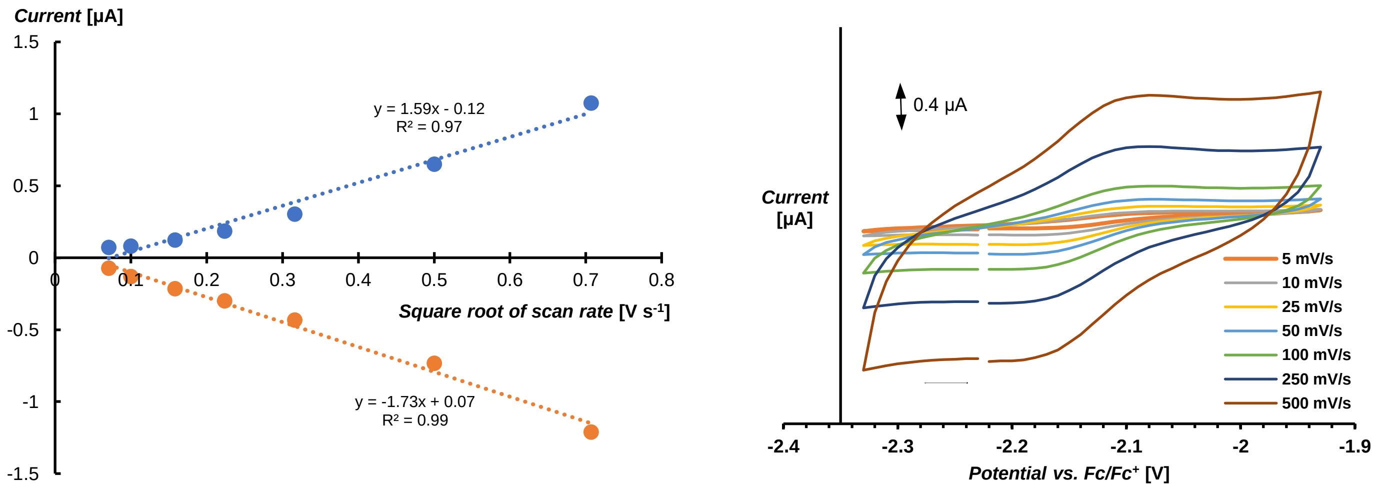

Figure S13. The Randles-Ševčík plot (left) suggests, despite of the weak signal, that the redox-event at −2.13 V (right; second scan of two is shown) is likely reversible.

2.2. Synthesis of 1 from (^{fun}CAAC)Pd(py)

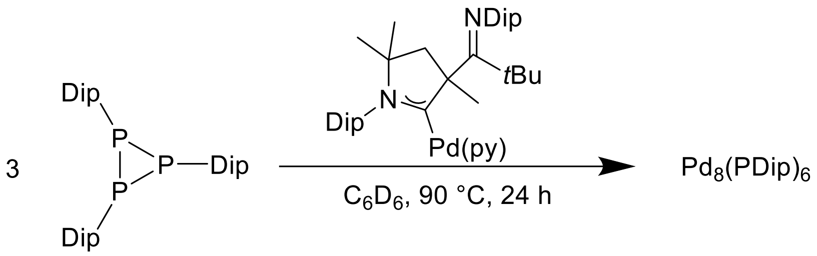

In a nitrogen-filled glovebox, (^{fun}CAAC)Pd(py) (30 mg, 0.043 mmol, 1 equiv.) and 1,2,3-tris[2,6-bis(isopropyl)phenyl]triphosphorane (8 mg, 0.33 equiv.; or 6 mg, 0.011 mmol, 0.25 equiv.; or 18 mg, 1 equiv.) were dissolved in C_6D_6 and combined in a J-Young NMR tube at room temperature. The dark mixture was analyzed *via* ^{1}H NMR, ^{31}P NMR and ^{31}P{^{1}H} NMR immediately and after 13 h. The reaction mixture was heated to 60 °C for 2 h and again analyzed *via* NMR, followed by additionally 1 h at 90 °C, and another NMR spectroscopic analysis. Upon cooling, a dark solid precipitated. Workup may be performed as indicated under 2.1. The spectra obtained with the two different concentrations of $(PDip)_3$ were essentially equivalent, thus revealing that the stoichiometry of $(PDip)_3$ (0.25 *vs.* 0.33 equiv. *vs.* 1 equiv.) does not have a strong effect on the outcome of the reaction.
The starting material $(PDip)_3$ reacted already at room temperature to new species (#, 314.21 (d, 114.8 Hz); §, −1.14 (d, 220.8 Hz); −3.31 (d, 217.7 Hz)), which after 13 h partially converted to further intermediates (%, 111.88 (dd, 182.4 Hz, 28.8 Hz); 56.34 (d, 182.0 Hz); 53.02 (d, 181.7 Hz)). Those intermediates are tentatively assigned to a four-membered PdP_3 oxidative addition product and follow-up multinuclear species. Besides, a three-membered PdP2 complex is known to be formed from $(C_2H_4)Pd(PPh_3)_2$ and $(tBuP)_3$ at room temperature.[3] Eventually, the title compound (*, 533.41 (s)) formed. An assumed separate reaction pathway exists *via* (+, −71.59 (s); −98.99 (s)) to the unidentified side products (&, −38.07 (s), −101.68 (s)). Note that the product at −101.68 ppm, in the non-^{1}H-decoupled ^{31}P spectrum shows a doublet (J = 231.6 Hz), indicating the formation of a P–H bond. This may be due to a cyclization pathway involving the isopropyl groups of the Dip group with the proton ending up on phosphorus. Eventually note that the synthesis with $Pd_2(dba)_3$, which is less reactive than (^{fun}CAAC)Pd(py), proceeds with higher selectivity.

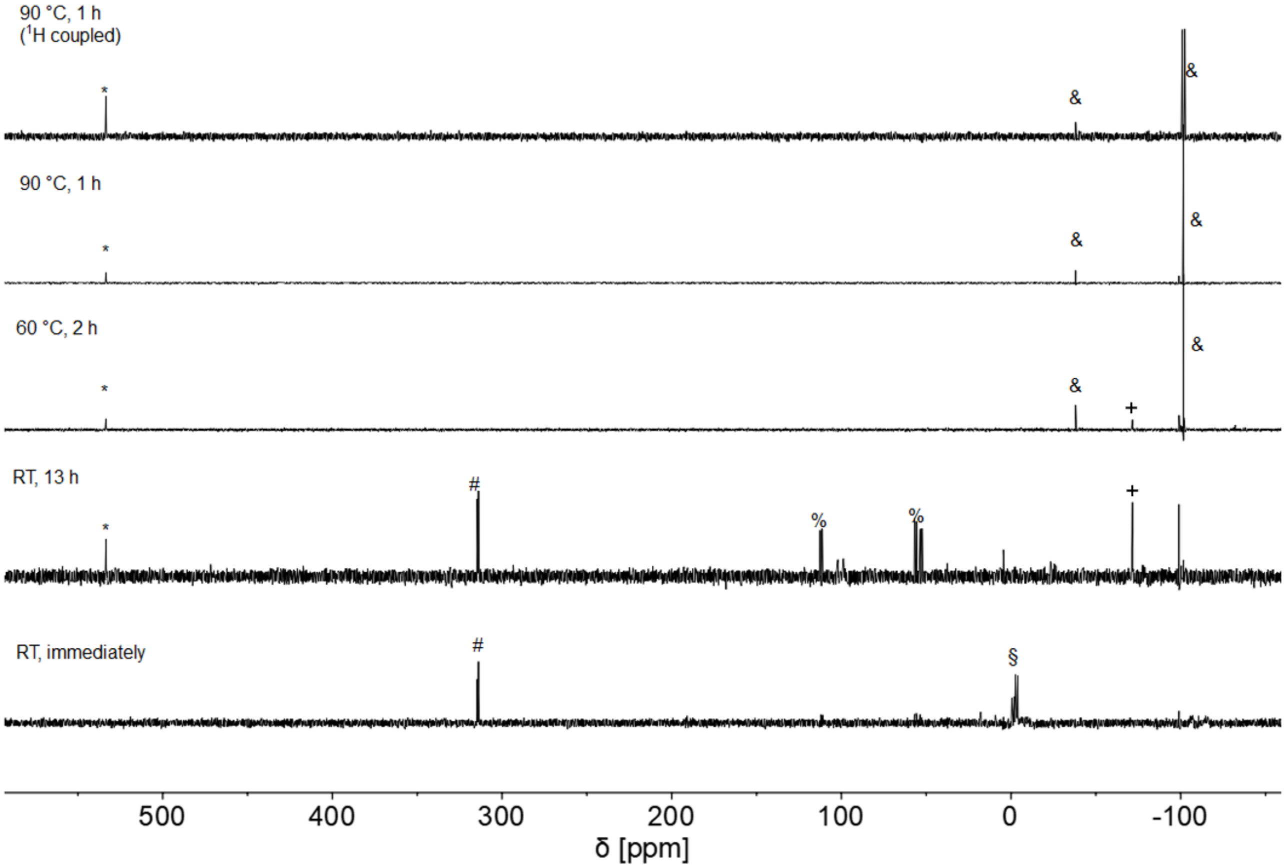

Figure S14. ^{31}P{^{1}H} NMR (162 MHz, C_6D_6) for the reaction of (^{fun}CAAC)Pd(py) with 0.33 equiv. of $(PDip)_3$.

2.3. Crystallization of Trapped Intermediates 2 and 3

$[Pd(py)_2(PDip)_3]$ **2**: In a nitrogen-filled glovebox, (^{fun}CAAC)Pd(py) (30 mg, 0.043 mmol, 1 equiv.) and 1,2,3-tris[2,6-bis(isopropyl)phenyl]-triphosphirane (24 mg, 0.042 mmol, 1 equiv.) were dissolved in melting pyridine-d_5 and kept at RT overnight. During this time, the complex crystallized from the solution as dark-orange platelets.

$[Pd_2(py)_2(PDip)_2(PDip)_3]$ **3**: In a nitrogen-filled glovebox, (^{fun}CAAC)Pd(py) (30 mg, 0.043 mmol, 1 equiv.) and 1,2,3-tris[2,6-bis(isopropyl)phenyl]triphosphirane (8 mg, 0.014 mmol, 0.33 equiv.) were dissolved in melting toluene-d_5 and kept at RT overnight. From the dark reaction mixture, deep dark-red crystals were obtained through vapor diffusion of pentane into the toluene solution at −35 °C.

Both pyridine complexes **2** and **3** are insoluble in a wide range of solvents, thus preventing further analysis.

2.4 Stability of 1 in Aerated Benzene

Solid nanocluster **1** was kept on air for 48 hours (without signs for decomposition), and then dissolved in boiling, dry C_6D_6. The sample dissolved quantitatively, and no signs for decomposition were obtained in the ^{1}H NMR spectroscopic analysis.
Nanocluster **1** is not soluble enough in organic solvents at room temperature to obtain an appropriate ^{1}H NMR spectrum. Therefore, 3 mg of **1** were dissolved 0.5 mL of boiling and dry C_6D_6. The sample contained hexamethylbenzene and traces of silicon grease, which were used as internal standard. A ^{1}H NMR spectrum was recorded, and the solution was then kept open to air for 5 min, and several times shaken vigorously. The tube was left open to air (it was shaken vigorously every 30 min to minimize diffusion effects), and the decay/precipitation of **1** was followed by ^{1}H NMR spectroscopy over 12 h (Fig. S16). A fine brown-red precipitate of **1** (Fig. S15, A and B) formed. After 12 h, about 25% of **1** remained in solution. It was again heated to reflux to redissolve all of **1**, whereby a minor amount of colorless residue formed at the wall of the NMR tube (Fig. S15, C), and the ^{1}H NMR spectroscopic analysis of the homogeneous sample confirmed the recovery of overall 75% of **1** in solution in respect to the starting concentration, as well as the presence of approximately 12 equiv. of water (Fig. S16). The NMR-tube was sealed again, and the further decay of **1** was monitored over the course of 50 more hours (Fig. S16), upon which about 15% of **1** remained in solution. Even after further heating to reflux for 2 h (closed valve), about 45% of **1** remained in solution (Fig. S15, D; Fig. S16). Quantitative decomposition of the sample (with the concomitant formation of some colorless precipitate, palladium black and the change of the solution's color from red to yellow) was obtained after heating to reflux for another 24 h (Fig. S15, E; Fig. S16). We conclude that the half-life $t_{1/2}$ of **1** dissolved in C_6D_6 at room temperature amounts to at least 3 days.

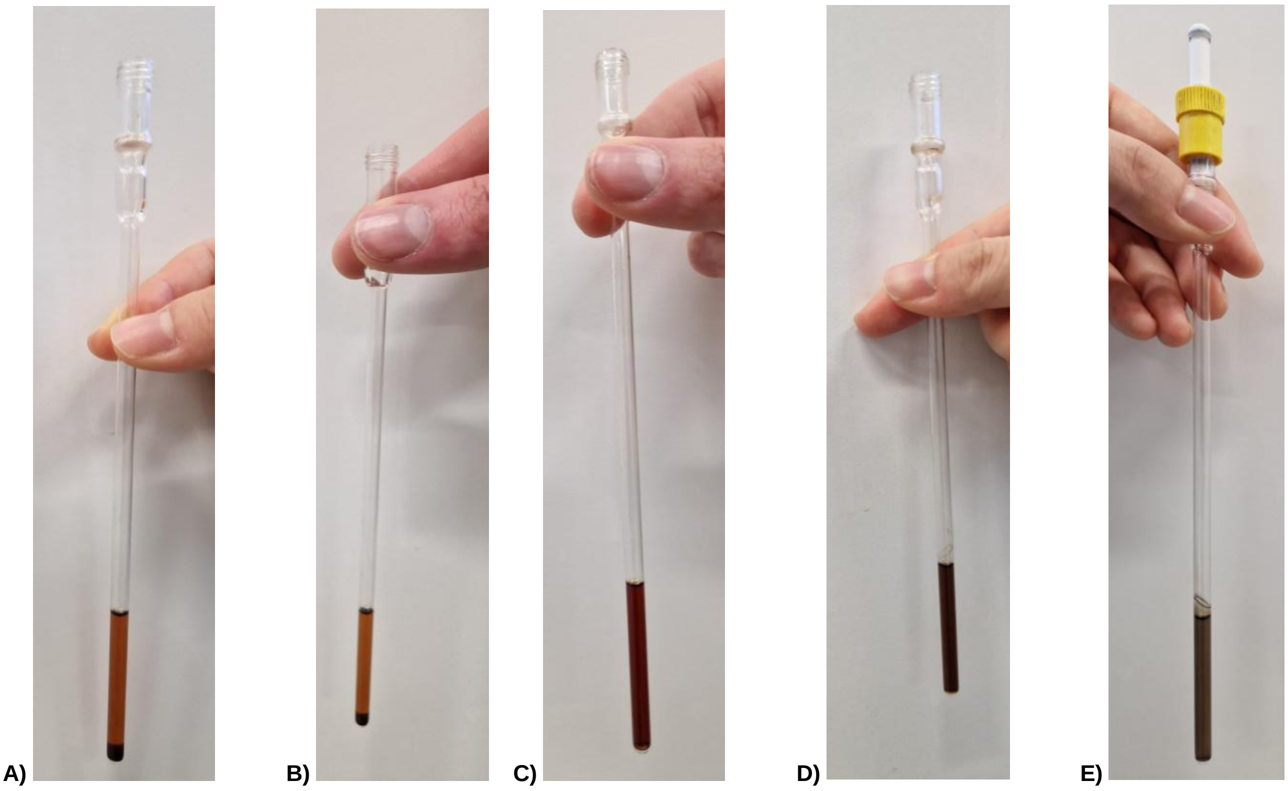

A) B) C) D) E)

Figure S15. Picture of a sample of **1** in C_6D_6 after 4 hours on air and with a red-brown precipitate of **1** (A), after 8 h on air (B), after redissolution of the precipitate through heating after 12 h (C), after further 3 days at room temperature and heating to reflux for 2 hours (D), and after heating to reflux for another 24 hours (E).

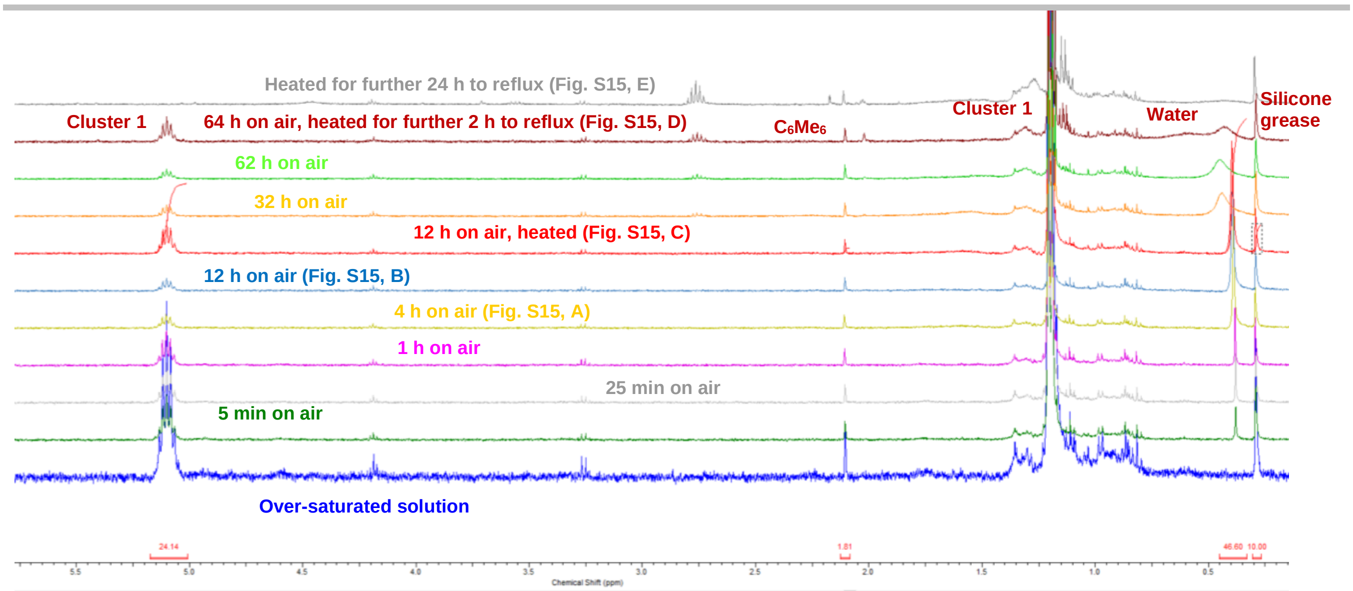

Figure S16. Precipitation/decay of **1** in C_6D_6 on air over the course of 12 h (and additional 50 hours + 2 h heating + 24 h heating) as obtained by ^{1}H NMR (400 MHz, C_6D_6).

3. Synthesis and Characterization of 4 [$Pd_8(PDip)_6(CNoXyl)_4$]

$$Pd_8(PDip)_6 + 4\ CN\textit{o}Xyl \xrightarrow[C_6D_6]{rt} Pd_8(PDip)_6(CN\textit{o}Xyl)_4$$

In a nitrogen-filled glovebox, **1** (6 mg, 0.003 mmol, 1 equiv.) was dissolved in 1 mL of C_6D_6. A stock solution of 2,6-dimethylphenylisocyanide (9 mg dissolved in 1 mL of C_6D_6) was prepared and 210 µL (2.36 mg, 0.018 mmol, 6 equiv.) were added. ^{31}P and ^{1}H NMR spectra were recorded, indicating the clean and quantitative formation of the desired product. The addition of 10 ml of hexanes precipitated the dark-brown product. The suspension was filtered over celite, which was washed with 10 ml of hexanes. The product was then recovered by rinsing with 2 mL of C_6H_6. Volatiles were removed *in vacuo* to afford a dark-brown solid. Single crystals suitable for X-ray diffractometry were obtained by vapor diffusion of pentane into a solution in C_6H_6. Yield: 6 mg (80 %).

^{1}H NMR (400 MHz, C_6D_6): δ = 1.46 (d, 72H, J = 6.12 Hz) ppm, 1.63 (s, 24H) ppm, 5.17 (q, 12H, J = 6.44 Hz) ppm, 6.59 (d, 8H, J = 7.50 Hz) ppm, 6.71 (t, 4H, J = 7.81 Hz), 7.18 (d, 12H, J = 8.20 Hz) ppm, 7.29 (t, 6H, J = 7.80 Hz) ppm.

^{31}P NMR (162 MHz, C_6D_6): δ = 519.35 (s) ppm.

^{13}C NMR (100 MHz, C_6D_6): δ = 18.17 (*i*Pr; CH_3), 25.97 (Ph-CH_3), 30.95 (*i*Pr; CH), 123.00 (Dip-CH^m), 128 (Ph-$CH^{m/p}$, superimposed with C_6D_6 signal, HMQC), 127.23 (Ph-C^o), 128.77 (Dip-C^o), 130.09 (Dip-CH^p), 134.98 (CN-C), 140.65 (C-P), 150.30 (C≡N) ppm.

Melting point: 280 °C (decomposition).

UV-Vis (C_6H_6, 0.62 × 10^{-5} M): 283 nm (197,200 M^{-1} cm^{-1}), 297 nm (173,500 M^{-1} cm^{-1}), 431 nm (88,800 M^{-1} cm^{-1}).

IR (nujol): $\tilde{\nu}$ = 2945 cm^{-1} (w), 2078 cm^{-1} (s), 1648 cm^{-1} (m), 1589 cm^{-1} (m), 1446 cm^{-1} (m).

APPI-HRMS: Despite various attempts, only the starting material **1** was detected with appropriate intensity. CN*o*Xyl ligands are likely removed during the ionization process.

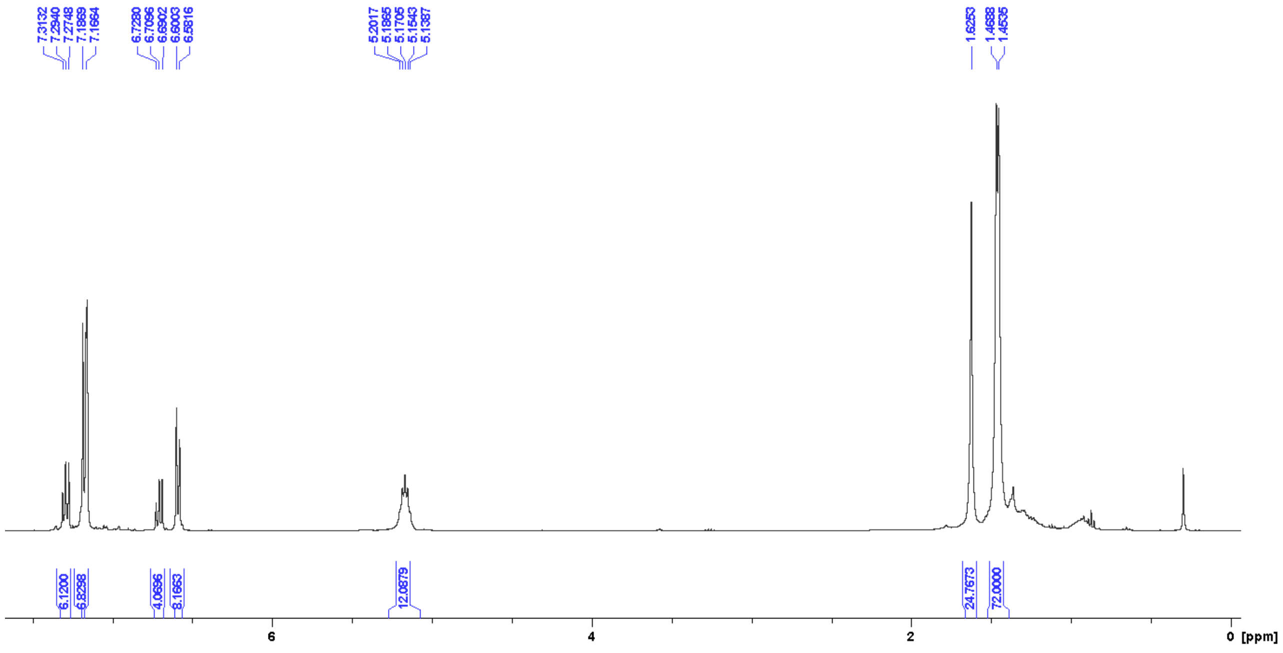

Figure S17. ^{1}H NMR (400 MHz, C_6D_6) spectrum of **4**.

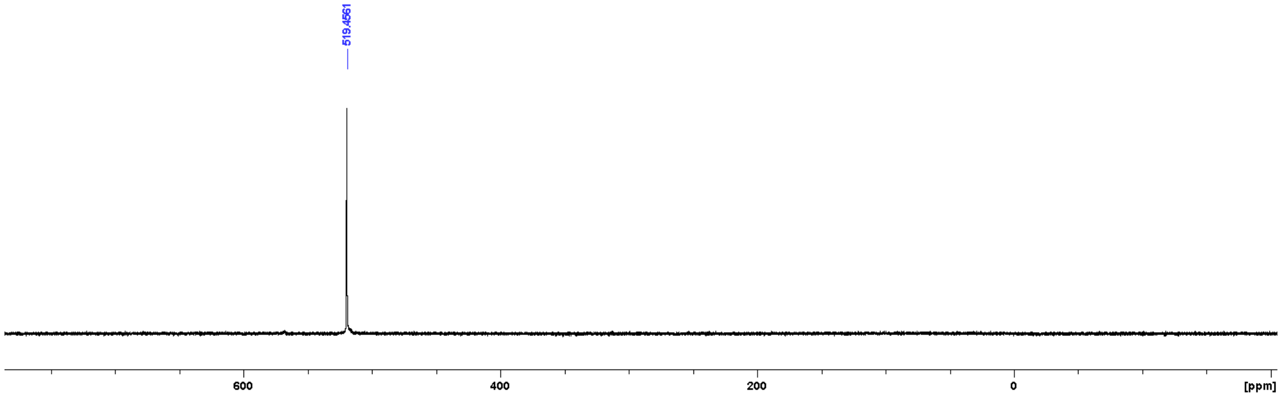

Figure S18. $^{31}P\{^{1}H\}$ NMR (162 MHz, C_6D_6) spectrum of **4**.

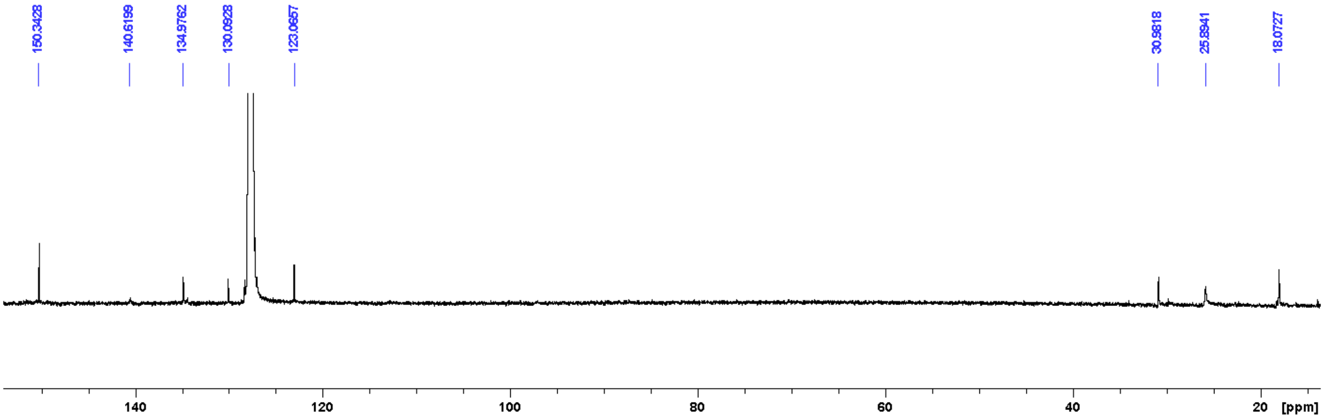

Figure S19. $^{13}C\{^{1}H\}$ NMR (101 MHz, C_6D_6) spectrum of **4**.

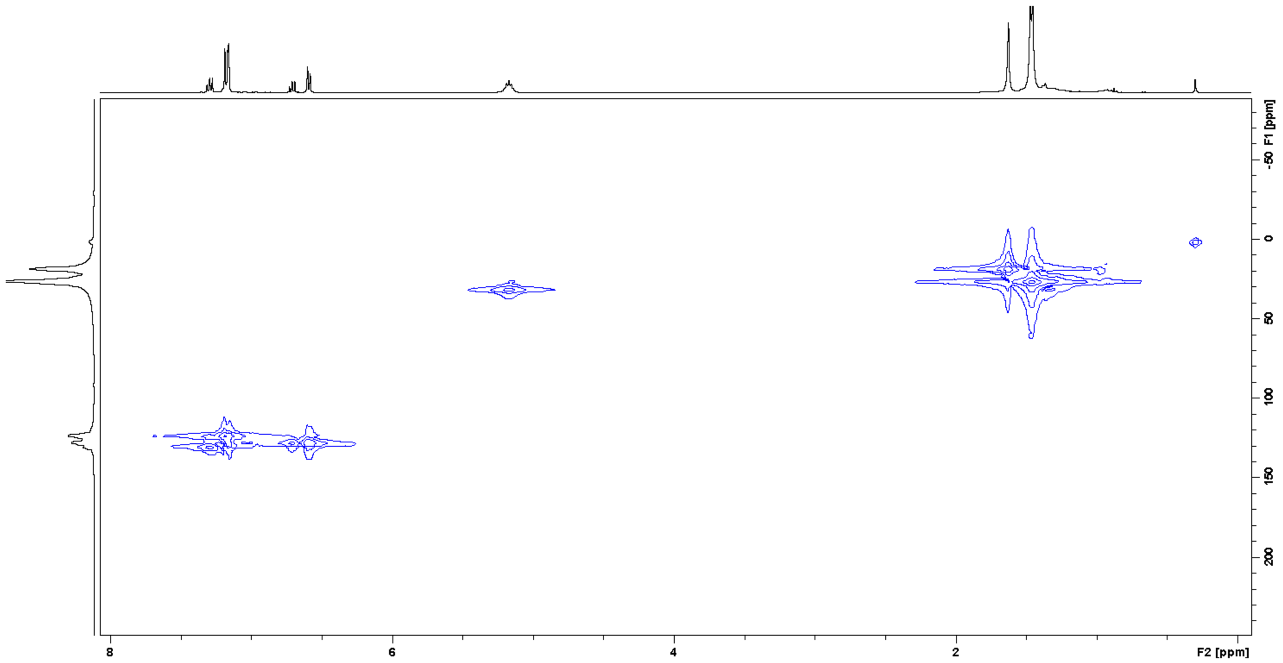

Figure S20. HMQC(^{1}H,^{13}C) NMR (C_6D_6) spectrum of **4**.

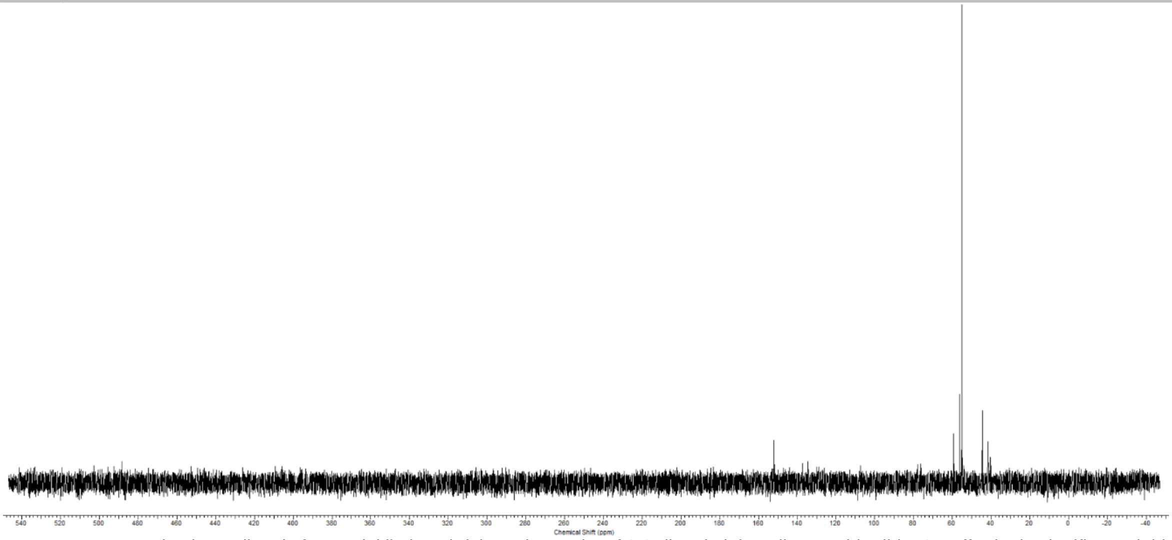

Figure S21. Attempts to synthesize **4** directly from $Pd_2(dba)_3$, P(Dip)$_3$ and 4 equiv. of 2,6-dimethylphenylisocyanide did NOT afford **4** in significant yield according to the ^{31}P NMR spectrum (C_6D_6) of the crude reaction mixture.

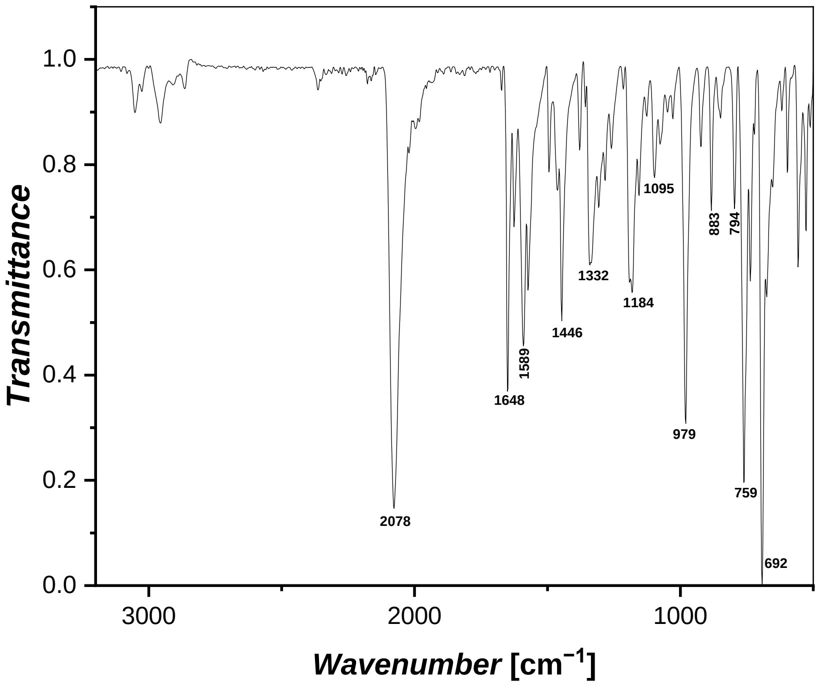

Figure S22. IR spectrum of **4**. The weak shoulder at the lower-energy side of the T_2 stretch at 2078 cm^{-1} is assigned to the A_1 stretch.

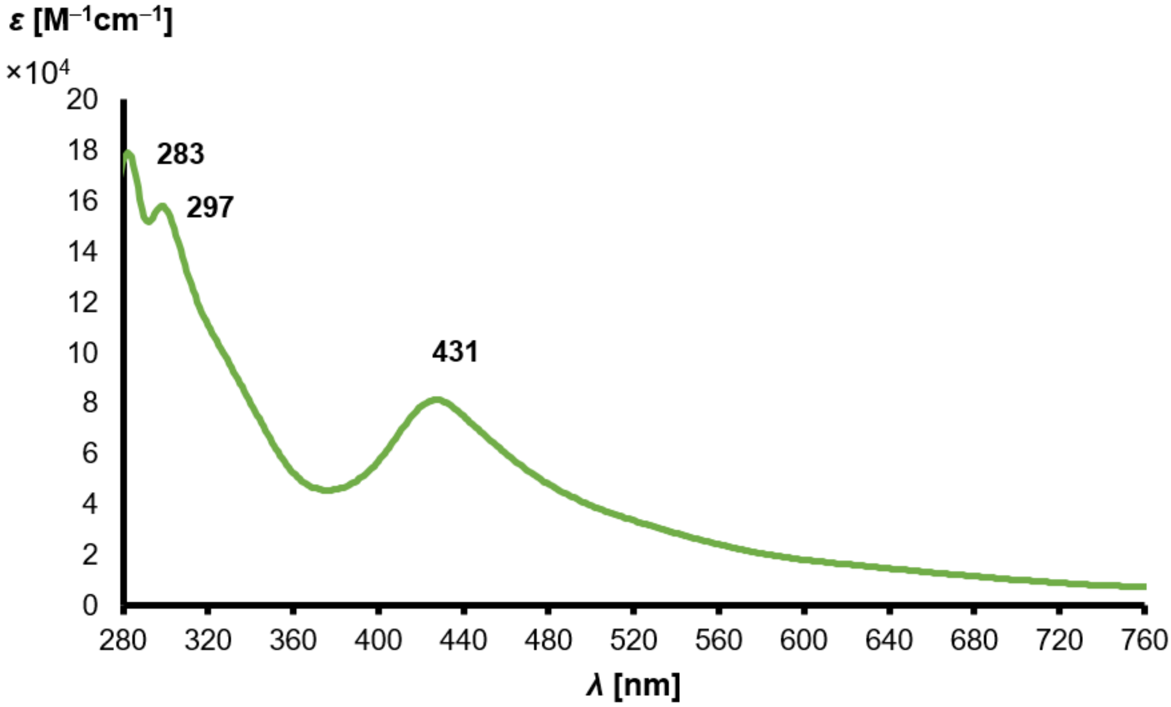

Figure S23. UV-Vis electronic absorption spectrum of **4** dissolved in C_6H_6, concentration 0.6×10^{-5} M.

4. Interaction of 1 with Ethylene

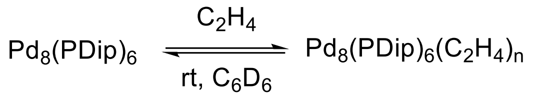

In a nitrogen-filled glovebox, **1** (3 mg, 0.0012 mmol) was dissolved in 0.5 mL of C_6D_6 and loaded in an NMR tube equipped with a J-Young valve. Using a Schlenk-line, the solution was saturated with ethylene gas and analyzed by NMR spectroscopy. In the ^{31}P spectrum, a shift of 4 ppm in respect to the starting material **1** was obtained (Fig. S24). In the ^{1}H spectrum, a slight shift of 0.2 ppm was observed for the CH signal of the *i*Pr groups (Fig. S25). Upon removal of volatiles *in vacuo* and redissolution in C_6D_6, the clean regeneration of **1** was obtained.

^{1}H NMR (400 MHz, C_6D_6): δ = 1.21 (d, 72H, ^3J = 6.82 Hz) ppm, 5.09 (q, 12H, ^3J = 6.61 Hz) ppm, 5.25 (s, excess of C_2H_4), ^3J = 6.61 Hz) ppm 7.12 (d, 12H, ^3J = 7.65 Hz) ppm, 7.27 (t, 6H, J = 7.70 Hz) ppm.

^{31}P NMR (162 MHz, C_6D_6): δ = 537.5 (s) ppm.

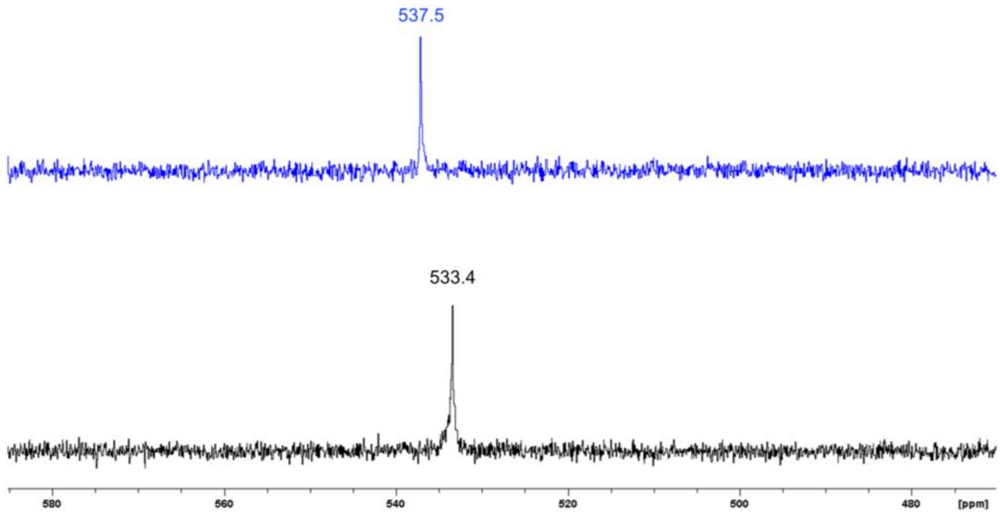

Figure S24. $^{31}P\{^{1}H\}$ NMR (400 MHz, C_6D_6) spectrum of **1** in the presence of ethylene (blue, top), stacked with the spectrum of **1** prior to the addition of ethylene (black, bottom).

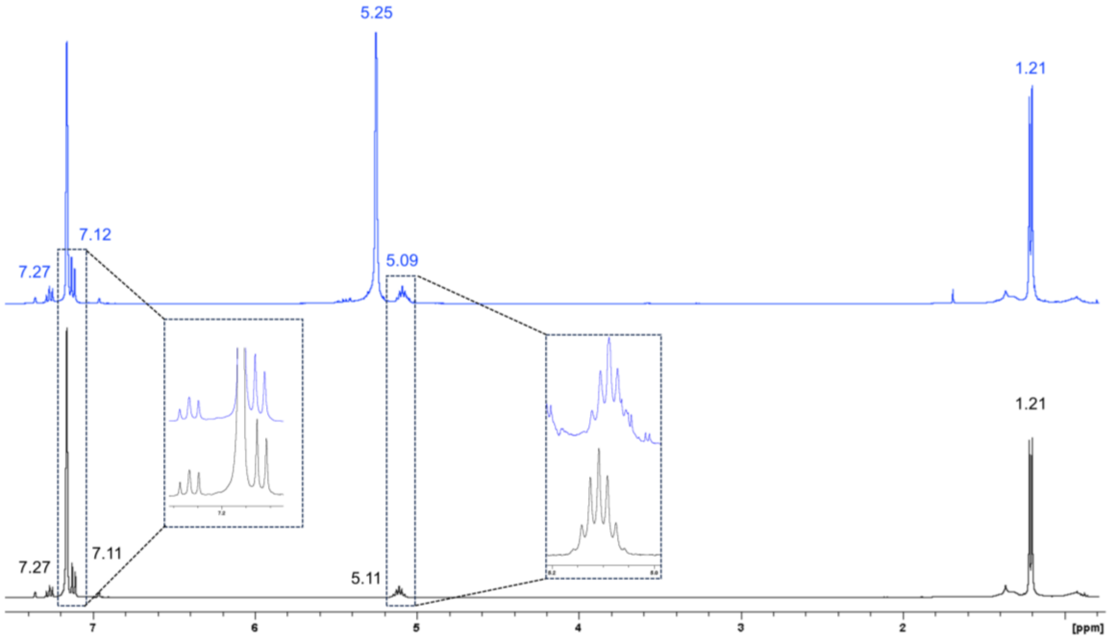

Figure S25. ^{1}H NMR (400 MHz, C_6D_6) spectrum of **1** in the presence of ethylene (blue, top), stacked with the spectrum of **1** prior to the addition of ethylene (black, bottom).

5. Titration of 1 with 2,3-Dimethylphenylisocyanide

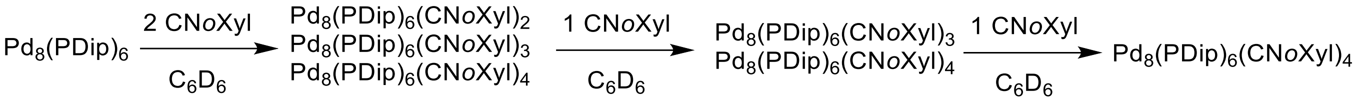

In a nitrogen-filled glovebox, **1** (6 mg, 0.003 mmol, 1 equiv.) was dissolved in 1 mL of C_6D_6. A stock solution of 2,6-dimethylphenyl-isocyanide (CN*o*Xyl; 9 mg, 6.9 mmol, dissolved in 1 mL of C_6D_6) was prepared and aliquots of 90 µL (2 equiv.), 135 µL (overall 3 equiv.), 180 µL (overall 4 equiv.) and 270 µL (overall 6 equiv.) were added to the cluster suspension consecutively. For each addition step, it the mixture was shaken vigorously, and ^{31}P- and ^{1}H-NMR spectra were recorded. Note that the starting cluster **1** is not well soluble in benzene. The number of coordinated isocyanides was determined using the integration value in the ^{1}H spectra as well as the number of signals in the ^{31}P NMR spectrum, where mono- and tris-coordinated species are expected to lead to two signals. Upon the addition of 2 equivalents, a mixture of three compounds was obtained, which we assign to the di-, tri-, and tetraisocyanide clusters. Addition of another equivalent leaves two clusters, which relate to $Pd_8(PDip)_6(CN\textit{o}Xyl)_3$ and $Pd_8(PDip)_6(CN\textit{o}Xyl)_4$. Upon the addition of overall 4 equiv., only $Pd_8(PDip)_6(CN\textit{o}Xyl)_4$ remains in solution. Adding more equivalents of isocyanide did not lead to further higher-coordinated species.

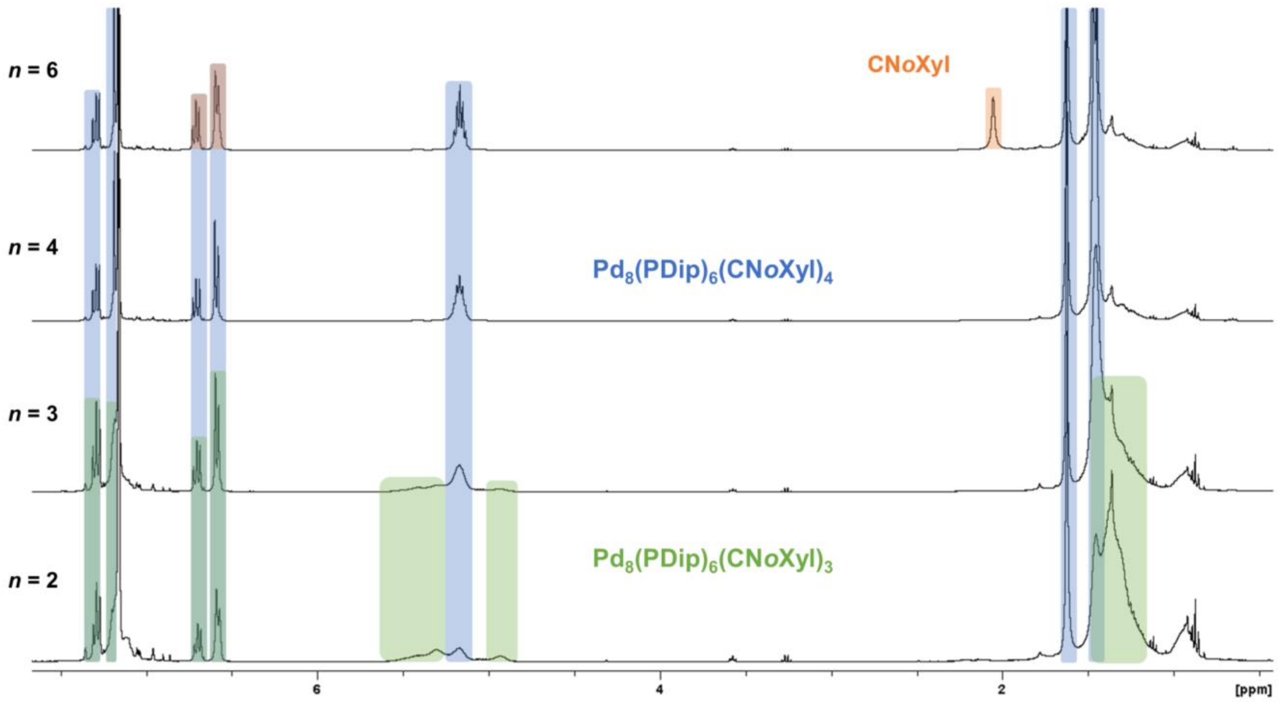

Figure S26. ^{1}H NMR (400 MHz, C_6D_6) spectra of **1** titrated with *n*-equivalents of 2,6-dimethylphenylisocyanide (CN*o*Xyl). $Pd_8(PDip)_6(CNoXyl)_3$, $Pd_8(PDip)_6(CN\textit{o}Xyl)_4$ (**4**) and uncoordinated CN*o*Xyl are highlighted in green, blue and orange color, respectively.

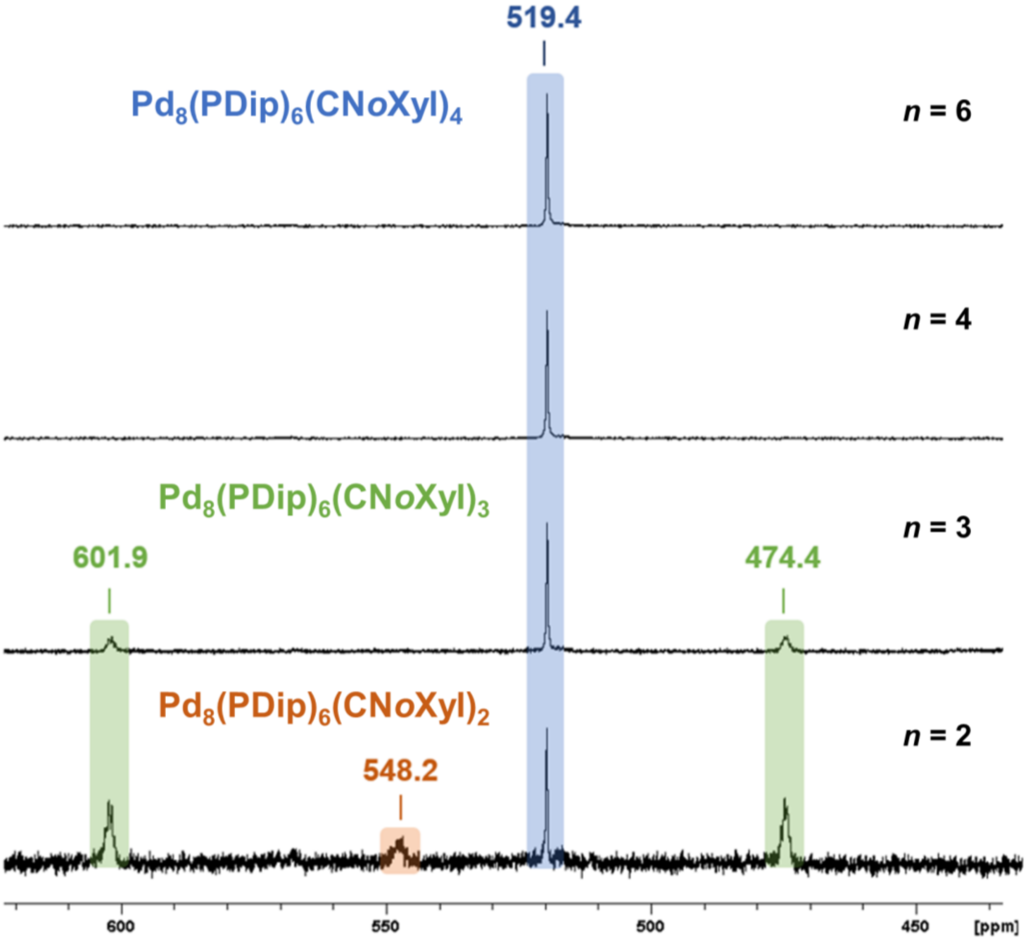

Figure S27. $^{31}P\{^1H\}$ NMR (162 MHz, C_6D_6) spectra of **1** titrated with *n*-equivalents of 2,6-dimethylphenylisocyanide (CN*o*Xyl). $Pd_8(PDip)_6(CNoXyl)_2$, $Pd_8(PDip)_6(CNoXyl)_3$ and $Pd_8(PDip)_6(CNoXyl)_4$ (**4**) are highlighted in orange, green and blue color, respectively.

6. Single Crystal Structure Elucidation and Refinement (SC-XRD)

X-ray Structure Determination: X-ray quality crystals were selected in Fomblin YR-1800 perfluoroether (Alfa Aesar) at ambient temperature. The samples were cooled to 150(2) K (except compound **4**: 143(2)K) during measurement. The data were collected on a Bruker D8 Venture diffractometer using monochromated MoKα (λ = 0.71073 Å; compound **4**) or CuK_{α} radiation (λ = 1.54178 Å; compounds **1**–**3**), respectively, and a Photon II detector. The structures were solved by intrinsic phasing (SHELXT)[4] and refined by full matrix least squares procedures (SHELXL) with the Olex2[5] or ShelXle[6] platform, respectively. Semi-empirical absorption corrections (multiscan and additional spherical absorption correction) were applied to the diffraction data recorded using the SADABS application within the APEX4 platform.[7] All non-hydrogen atoms were refined anisotropically, hydrogen atoms were included in the refinement at calculated positions using a riding model. All special refinement details (if required) for disordered or twinned structures as well as molecular structure representations are summarized below. A summary on standard crystallographic parameters as well as the CSD entry numbers within the Cambridge Crystallographic Data Centre (CCDC) is subsequently provided in Table S1.

Special Refinement Details:

*Compound **3**, disorder*: One of the Dip groups was found to be disordered, and a disorder model was described for tilting of the whole fragment containing C33, C32, C31, C71, C72 and C73. Two respective split positions have been refined according to the free variable 2 (FVAR2), which refined into occupancies of 0.57 and 0.43. To fix the refinement, soft DELU and ISOR restraints were embedded to the refinement reaching more reasonable displacement.

*Compound **4**, disorder*: One of the *o*Xylyl substituents is split over two positions according to the free variable 4 (FVAR4) (0.53:0.47). Two benzene solvent molecules share their position with two *n*-pentane solvent molecules. Their occupancy factors refined to 0.42:0.58 and 0.48:0.52, according to FVAR 2 and 3 respectively. For the refinement of the disorder restraints (RIGU, SADI, FLAT, SIMU, DFIX) were applied for the solvent molecules. *Twinning:* The structure was refined as a non-merohedral two-component twin. The twin matrix was found to be (1.000 -0.606 -0.124, 0.000 -1.000 0, -0.007 0.000 -1.000) from the DOMAIN routine of Apex4. The structure was solved using the hklf4 file and then refined to convergence using the hklf5 routine of SHELXL. The BASF value refined to 0.3964(5).

Molecular Structure Representations: All molecular structure representations in the ESI as well as the main article have been prepared with the Diamond software package,[8] mixed representation of ellipsoid plots as well as wires/sticks was chosen for clarity. All ellipsoids are represented at the 50% probability level (unless stated otherwise).

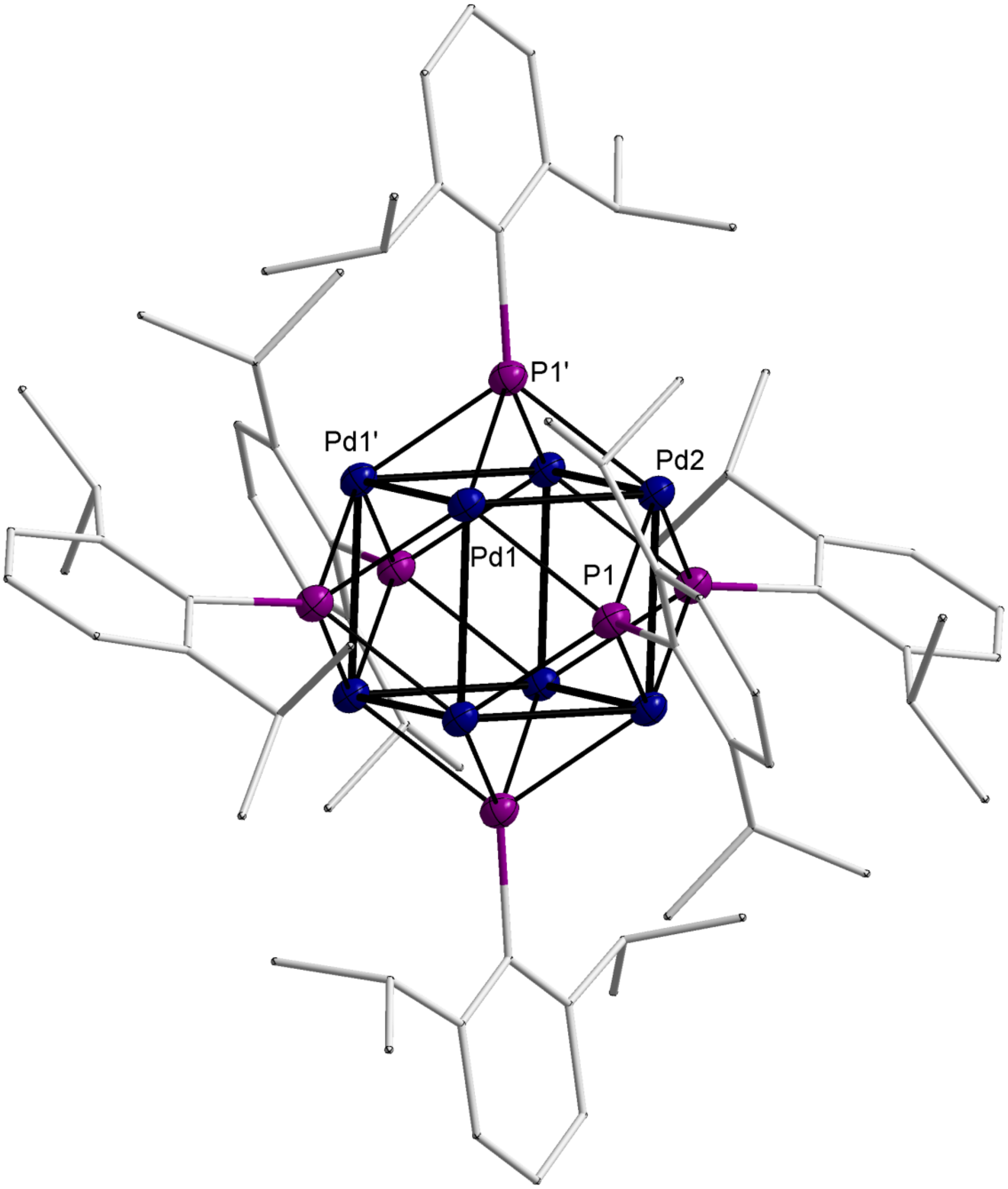

Figure S28. Molecular structure representation of **1**. Pd1' and P1' are symmetry generated over −1/3+y, 1/3−x+y, 1/3−z and −x+y, 1−x, z, respectively (arbitrarily chosen examples). Selected atom distances [Å] and angles [°]: Pd1–Pd2, 2.6997(6); Pd1–Pd1' 2.6867(6); Pd1–P1 2.332(2); Pd1'–P1' 2.341(2); Pd2–P1 2.3580(15); Pd1-P1-Pd2, 70.29(6); P1-Pd1-Pd1' 125.83(4).

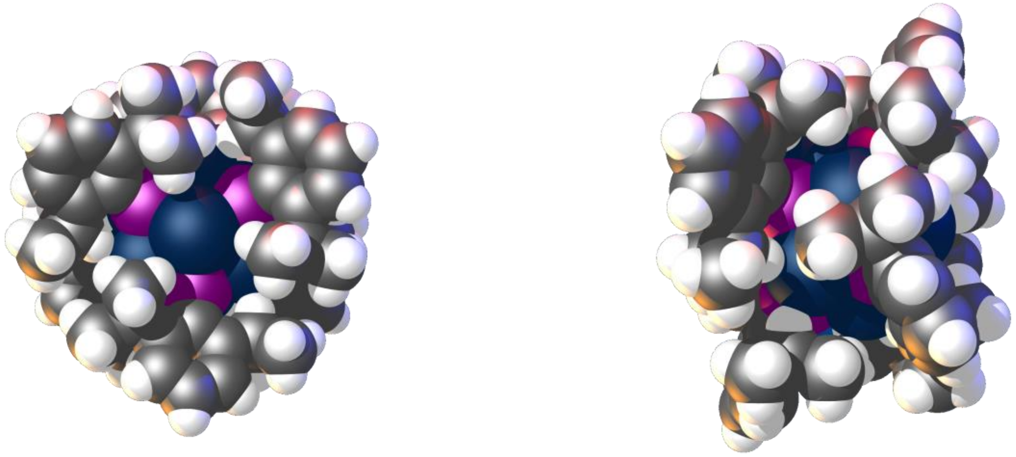

Figure S29. The space filling model of **1** illustrates that the symmetry associated with the $R\bar{3}$ space group is due to the Dip-substituents (cf. Figure S33).

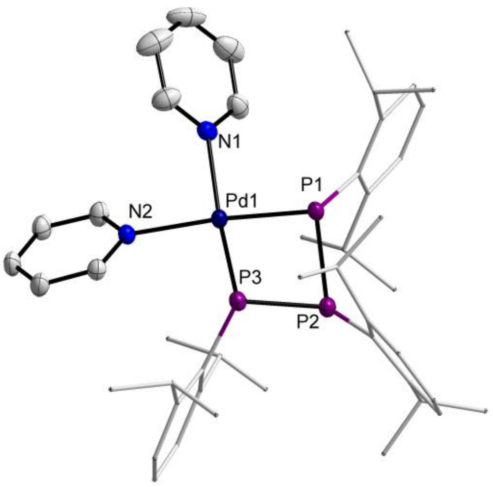

Figure S30. Molecular structure representation of **2**. Selected atom distances [Å] and angles [°]: Pd1–P1 2.3047(9), Pd1–P3 2.295(1), Pd1···Pd2 3.248(1), Pd1–N1 2.186(3), Pd1–N2 2.183(3), Pd1-Pd3-Pd2 92.39(4), Pd1-P1-P2 91.47(4), P1-P2-P3 79.01(4), N2-Pd1-N1 88.8(1).

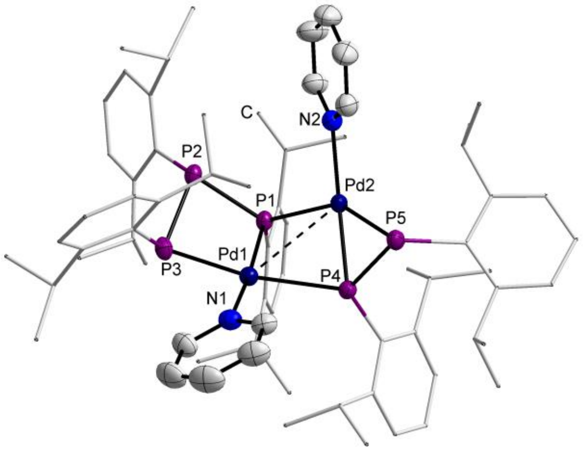

Figure S31. Molecular structure representations of **3**. Selected atom distances [Å] and angles [°]: P1–P2 2.2070(7), P2–P3 2.2124(8), P4–P5 2.1545(9), Pd1–P1 2.2762(7), Pd1–P3 2.3328(7), Pd1···Pd2 2.9940(5), Pd1–N1 2.174(2), Pd1–P4 2.5503(6), Pd2–P4 2.2639(6), Pd2–P5 2.399(8), Pd2–N2 2.205(2). P1-P2-P3 84.00(3), P1-Pd1-P3 79.82(2), P1-Pd2-P5 143.44(3), Pd1-P4-P5 119.67(3), P2-P1-Pd2 117.46(3), Pd2-P5-P4 59.32(3), Pd2-P4-P5 65.74(3).

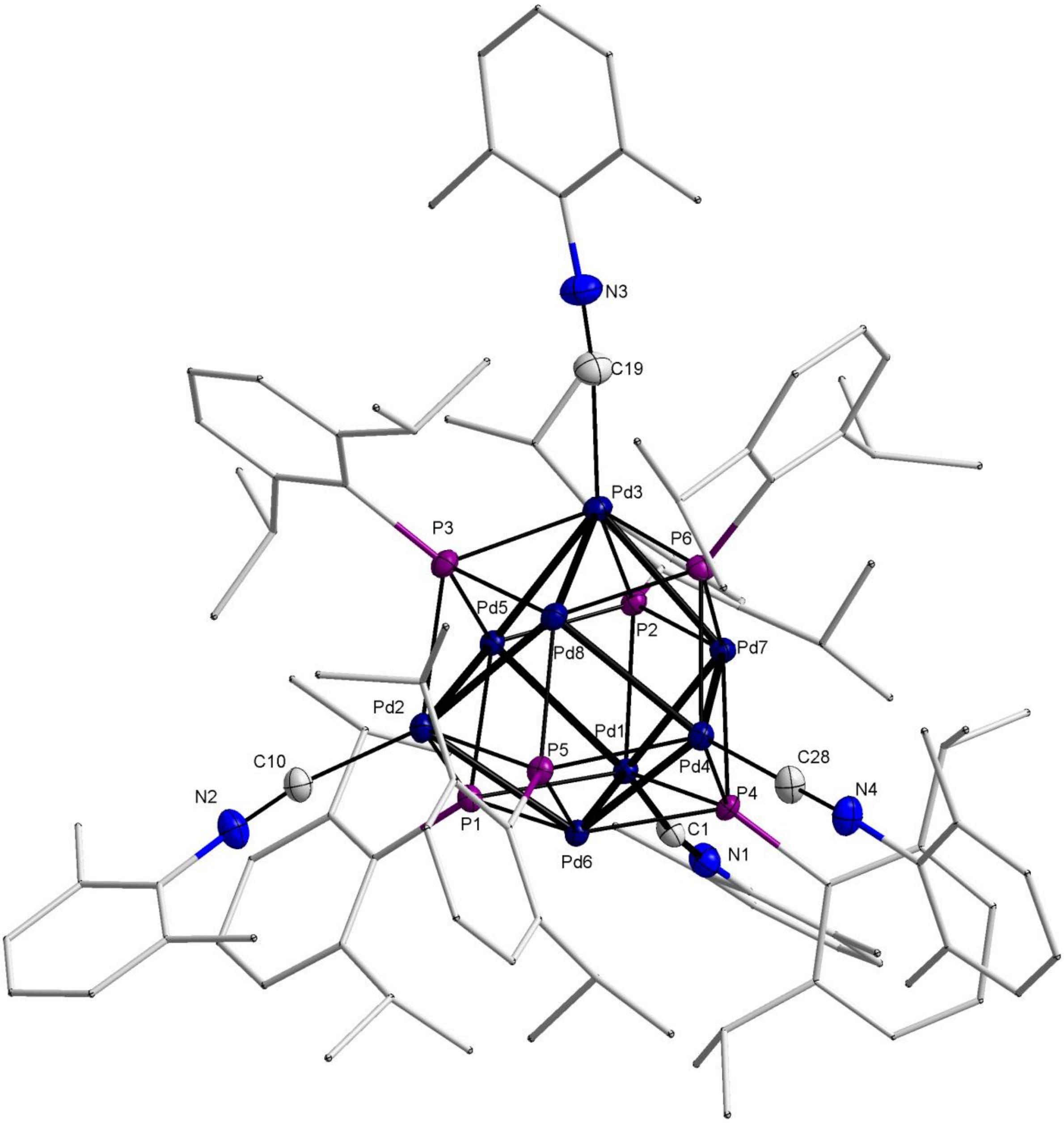

Figure S32. Molecular structure representation of **4**. Selected atom distances [Å]: Pd1–P1 2.4268(15), Pd1–P2 2.4373(15), Pd1–P4 2.4303(15), Pd1–Pd5 2.7591(6), Pd1–Pd6 2.7256(6), Pd1–Pd7 2.7557(6), Pd2–C10 2.011(6), Pd2–P3 2.423(2), Pd2–P5 2.427(2), Pd2–P1 2.4308(2), Pd2–Pd5 2.7417(6), Pd2–Pd6 2.7423(6), Pd2–Pd8 2.7284(6), Pd3–C19 2.028(7), Pd3–P2 2.468(2), Pd3–P3 2.459(2), Pd3–P6 2.424(2), Pd3–Pd5 2.7222(6), Pd3–Pd7 2.7425(6), Pd3–Pd8 2.7565(6), Pd4–C28 2.014(6), Pd4–P4 2.430(2), Pd4–P5 2.430(2), Pd4–P6 2.477(2), Pd4–Pd7 2.7357(6), Pd4–Pd8 2.7364(6), Pd4–Pd6 2.7518(6), Pd5–P1 2.271(2), Pd5–P2 2.285(2), Pd5–P3 2.297(2), Pd6–P4 2.300(2), Pd6–P5 2.300(2), Pd6–P1 2.318(2), Pd7–P2 2.283(2), Pd7–P4 2.290(2), Pd7–P6 2.291(2), Pd8–P3 2.283(2), Pd8–P6 2.286(2), Pd8–P5 2.292(2), N1–C1 1.165(8), N2–C10 1.154(8), N4–C28 1.162(8), C19–N3 1.152(9).

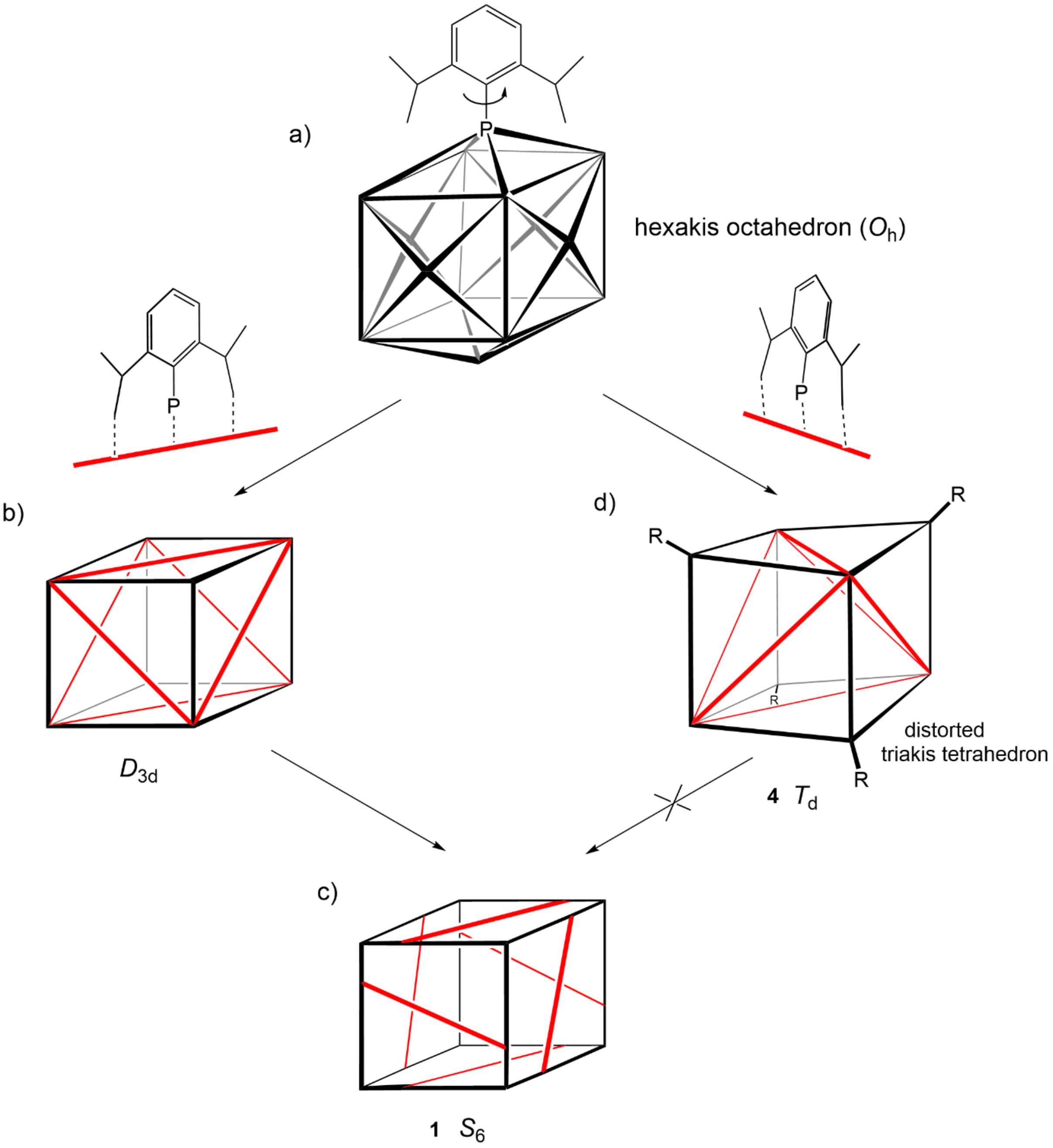

Figure S33. Symmetric relationship of cubic Pd_8 clusters in **1** and **4** with the inclusion of the orientation of the Dip-substituents. a) Rotation of the Dip groups in solution averages to O_h symmetry; b) D_{3d} point group for equal and diagonal orientation of all Dip groups; c) the subgroup S_6, as observed in the solid state for **1**, results by non-diagonal orientation of the Dip groups, which removes one mirror plane (*cf.* Figure S29); d) the two symmetry-unrelated tetrahedra in **4** afford a triakistetraeder, S_6 is not a subgroup.

Summary of X-ray Crystallographic Refinement:

Table S1. Crystallographic details for **1**–**4**.

Compound	1	2	3	4
Empirical formula	$C_{72}H_{102}P_6Pd_8$	$C_{46}H_{61}N_2P_3Pd$	$C_{70}H_{95}N_2P_5Pd$	$C_{118.91}H_{156.55}N_4P_6Pd_8$
Formula weight	2004.55	841.27	1332.12	2678.94
Temperature/K	150(2)	150(2)	150(2)	143(2)
Crystal system	trigonal	monoclinic	monoclinic	triclinic
Space group	*R*–3	*P*2₁/*c*	*P*2₁/*c*	*P*–1
a/Å	24.4510(8)	10.9572(2)	16.3306(3)	15.5015(6)
b/Å	24.4510(8)	16.0580(3)	14.3551(3)	16.3071(6)
c/Å	10.4546(6)	24.8673(5)	33.8651(6)	24.4515(8)
α/°	90	90	90	84.533(2)
β/°	90	102.3760(10)	90.4070(10)	82.6620(10)
γ/°	120	90	90	70.8390(10)
Volume/Å^3	5412.9(5)	4273.74(14)	7938.7(3)	5781.3(4)
Z	3	4	4	2
ρ_{calc}g/cm^3	1.845	1.307	1.115	1.539
μ/mm^{-1}	17.260	4.807	4.862	2712.0
F(000)	2976.0	1768.0	2784.0	2712.0
Crystal size/mm^3	0.21 × 0.08 × 0.05	0.24 × 0.22 × 0.05	0.26 × 0.24 × 0.19	0.260 × 0.200 × 0.100
Radiation	CuKα (λ = 1.54178)	CuKα (λ = 1.54178)	CuKα (λ = 1.54178)	MoKα (λ = 0.71073)
2*Θ* range for data collection/°	7.23 to 133.21	6.598 to 133.36	5.218 to 133.298	3.794 to 56.646
Index ranges	-28 ≤ h ≤ 29, -29 ≤ k ≤ 29, -12 ≤ l ≤ 12	-12 ≤ h ≤ 13, -19 ≤ k ≤ 19, -29 ≤ l ≤ 29	-19 ≤ h ≤ 19, -17 ≤ k ≤ 17, -40 ≤ l ≤ 40	-20 ≤ h ≤ 20, -21 ≤ k ≤ 21, 0 ≤ l ≤ 32
Reflections collected	20219	35130	106743	28765
Independent reflections	2140 [R_{int} = 0.0873, R_{sigma} = 0.0374]	7440 [R_{int} = 0.0790, R_{sigma} = 0.0499]	14020 [R_{int} = 0.0550, R_{sigma} = 0.0277]	28765 [R_{int} = *, R_{sigma} = 0.0413]
Data/restraints/parameters	2140/0/134	7440/0/481	14020/43/762	28765/884/1434
Goodness-of-fit on F^2	1.088	1.042	1.044	1.194
Final R indexes [*I*>=2σ (I)]	R_1 = 0.0399, wR_2 = 0.0882	R_1 = 0.0413, wR_2 = 0.0822	R_1 = 0.0278, wR_2 = 0.0659	R_1 = 0.0441, wR_2 = 0.0917
Final R indexes [all data]	R_1 = 0.0555, wR_2 = 0.0979	R_1 = 0.0635, wR_2 = 0.0925	R_1 = 0.0342, wR_2 = 0.0684	R_1 = 0.0664, wR_2 = 0.1040
Largest diff. peak/hole / e Å^{-3}	3.70/-0.90	0.69/-0.76	1.17/-0.86	1.50/-0.86
Absolute structure parameter	-	-	-	-
CCDC #	2287222	2287223	2287224	2287552

* refined as a two-component twin.

7. X-Ray Photoelectron Spectroscopy (XPS)

Experimental Details: The XPS experiments were performed with a VG ESCALab MkII spectrometer which was upgraded with a hemispherical 180° type analyzer and a multichannel plate detector system (PreVac EA 15). For excitation, the Al-K_α radiation (photon energy 1486.6 eV) of an Al/Mg twin anode was used. For survey spectra, the pass energy was set to 50 eV, while for detailed spectra of C1*s*, Pd3*d* and P2*p* the pass energy was set to 20 eV. All spectra were recorded in normal emission mode. For quantitative analysis of the intensity distribution of **1** (Pd *vs.* P), a Shirley background was applied[9] and the intensities were scaled with the photoemission cross sections by Yeh and Lindau.[10] From the Pd3*d* data (Fig. S35) and the P2*p* data (Fig. S36, black) a Pd : P ratio of 8.00 : 5.99 is achieved, which is in excellent agreement with the theoretical ratio in **1**. The samples were prepared as thin powder-films glued on conductive carbon pads. Due to the charging of the powder samples, the binding energies were rescaled *via* the C1*s* peak from the aromatic ligands in reference to the C1*s* peak from an HOPG sample (C1*s* at 284.3 eV), see Fig. S34. Due to a strong degassing of the powder samples, the pressure in the analysis chamber increased by about two orders of magnitude to approximately 5×10^{-8} mbar.

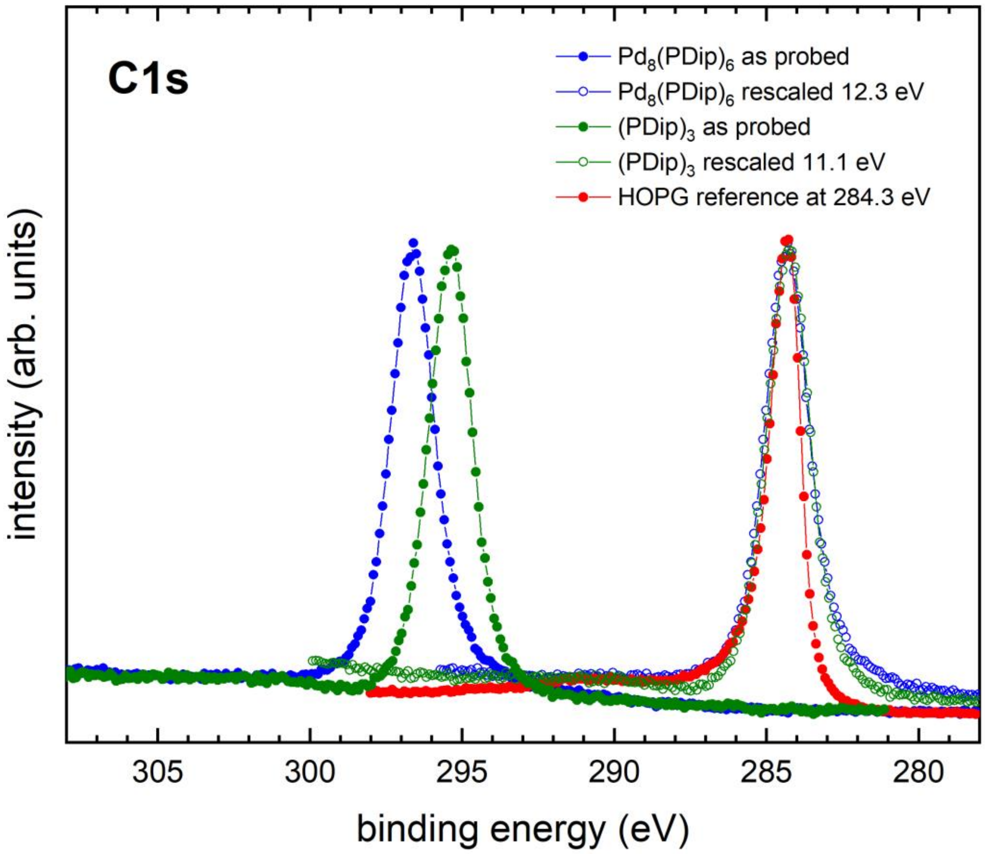

Figure S34. Applied calibration of energy based on the C 1*s* peak of an HOPG reference sample at 284.3 eV. The overall shift is 12.3 eV for $Pd_8(PDip)_6$ (**1**), and 11.1 eV for $(PDip)_3$.

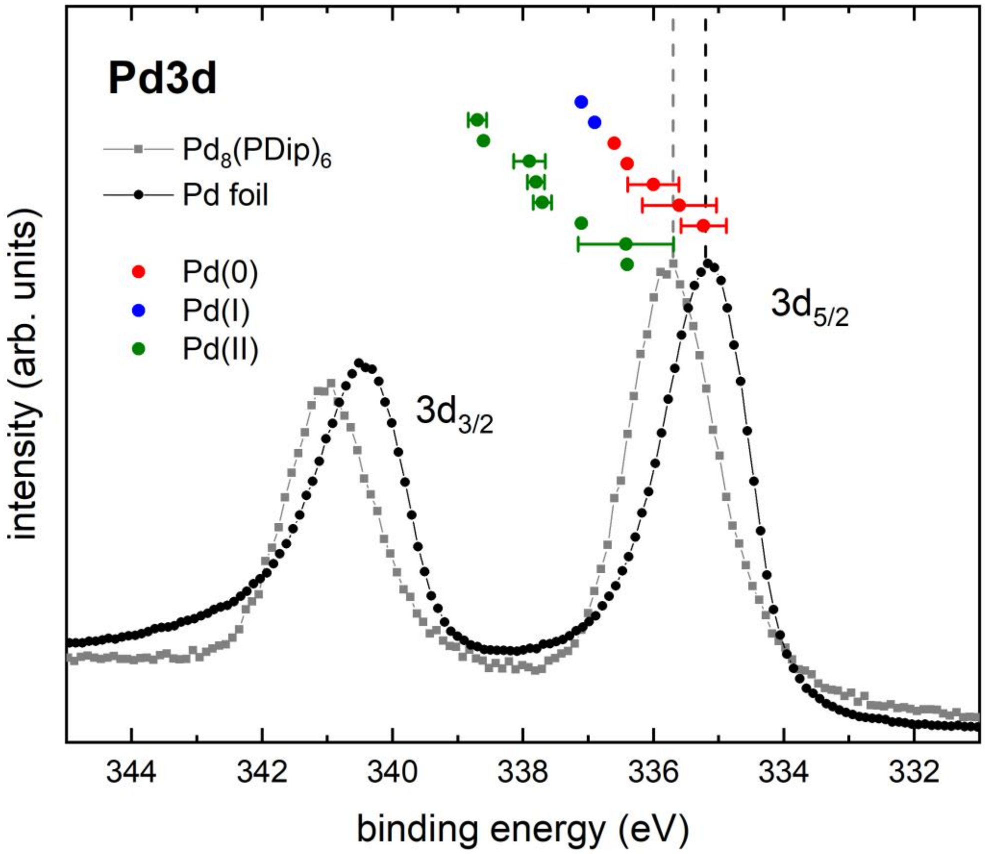

Figure S35. XPSpectrum of Pd3*d* of **1** after rescaling by the C1*s* shift (12.3 eV), in reference to the Pd3*d* spectrum from a Pd foil. The colored dots represent the mean values of Pd3$d_{5/2}$ binding energies (with standard deviation) for the Pd(0), Pd(I) and Pd(II) reference compounds listed in Table S2.

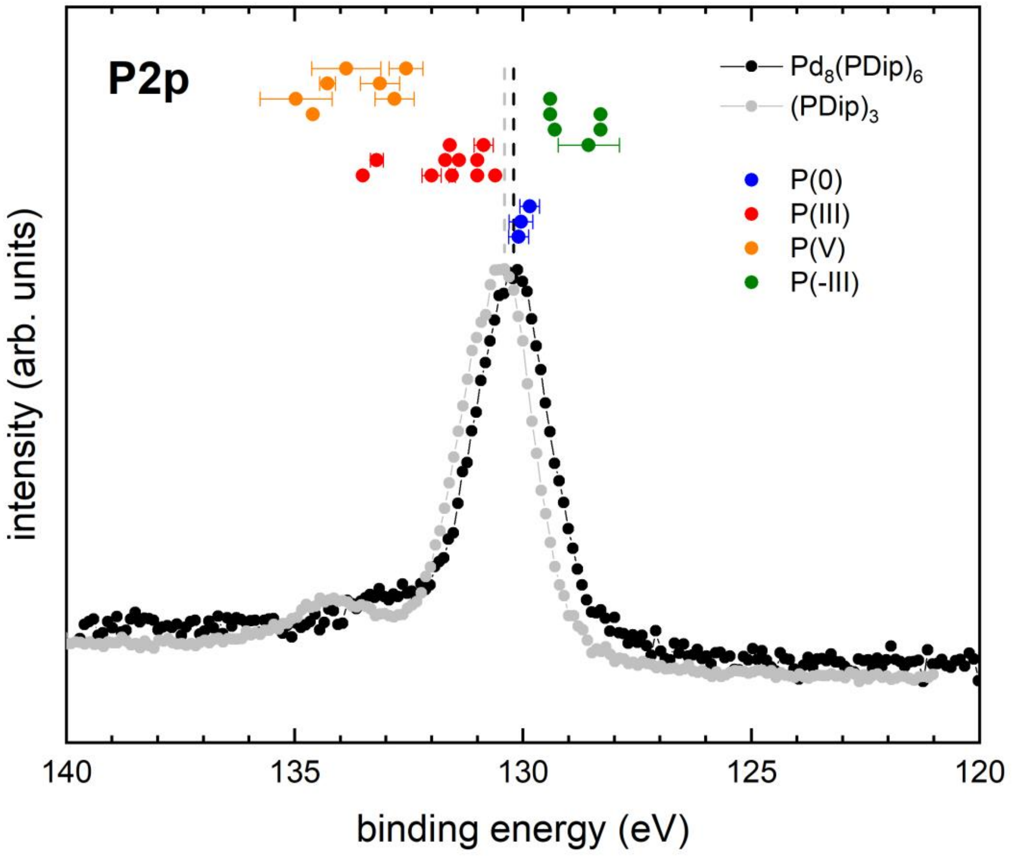

Figure S36. XPSpectra of P2*p* of **Pd$_8$(PDip)$_6$** (**1**) and **(PDip)$_3$**. The colored dots represent the mean values of P2*p* binding energies (with standard deviation) for the P(−III), P(0), P(III) and P(V) compounds listed in Table S3.

Table S2. Literature survey based on the NIST X-ray Photoelectron Database (SRD 20), Version 5.0,[11] for energy of 3$d_{5/2}$ peak for formal palladium(II), palladium(I) and palladium(0) complexes as well as the root mean square deviation RMSD and sample standard deviation (in parenthesis) in case of multiple reports. Values are given in [eV].

**Palladium(II)**											**Average**	**RMSD (sample standard deviation)**
PdCl$_2$(PPh$_3$)$_2$	337.80	337.70	338.00	337.80							337.83	0.11 (0.13)
PdI$_2$	336.40										336.40	n.a.
PdBr$_2$	337.10										337.10	n.a.
PdCl$_2$	338.00	338.40	337.80	337.80	337.80	337.70	337.80				337.90	0.22 (0.24)
PdF$_2$	337.70	337.50									337.60	0.10 (0.14)
Pd(OAc)$_2$	338.80	338.60									338.70	0.10 (0.14)
Pd(OTf)$_2$	338.60										338.60	n.a.
PdO	335.90	337.20	336.90	335.90	337.10	337.10	337.00	336.30	335.60	335.20	336.42	0.69 (0.73)
**Palladium(I)**											**Average**	**RMSD (sample standard deviation)**
Pd$_2$(OAc)$_2$(PPh$_3$)$_2$	336.90										336.90	n.a.
Pd$_2$Cl$_2$(dppm)	337.10										337.10	n.a.
**Palladium(0)**											**Average**	**RMSD (sample standard deviation)**
Pd foil	335.20	335.20	335.10	335.30	335.20	335.10	335.60	335.20	335.50	335.20	335.26	0.16
Pd/C	336.40	336.30	335.60	336.30	335.60	335.50	336.00				335.96	0.36 (0.39)
Pd$_2$(PPh$_3$)$_2$											336.60	n.a.
Pd$_2$(dba)$_3$*CHCl$_3$											336.40	n.a.
Pd(PPh$_3$)$_4$	335.20	336.00									335.60	0.40 (0.57)

Table S3. Literature survey based on the NIST X-ray Photoelectron Database (SRD 20), Version 5.0,[11] for energy of 2$p_{3/2}$ (2p, respectively in case of insufficient resolution) peaks for various pertinent phosphorus compounds and complexes as well as the root mean square deviation RMSD and sample standard deviation (in parenthesis) in case of multiple reports. Values are given in [eV].

**Phosphorus(V)**											**Average**	**RMSD (sample standard deviation)**
[Ph_4P]Br	132.7	132.8	133.5	133.5							133.13	0.38 (0.43)
KH_2PO_4	133.7	134.7	133.2								133.87	0.62 (0.76)
$NaPO_3$	134.3	134.2	134.5	134.1							134.28	0.15 (0.17)
P_2O_5	133.5	134.7	135.6	135.2	135.2	135.6					134.97	0.72 (0.79)
$OPPh_3$	132.2	132.3	132.3	132.4	132.5	132.5	132.6	132.6	132.7	133.5	132.56	0.35 (0.37)
Na_3PO_4	133.0	133.6	132.8	132.3	132.5	132.9	132.55				132.81	0.39 (0.43)
[$NPCl_2$]$_3$	134.6										134.60	n.a.
**Phosphorus(III)**											**Average**	**RMSD (sample standard deviation)**
[Au(PPh_3)$_4$]ClO_4	131.0										131.0	n.a.
[Au(PPh_3)Cl]	132.0										132.0	n.a.
[Pd(P(C_6H_5)$_3$)$_4$]	130.6										130.6	n.a.
[Pd(CN)$_2$(PPh_3)$_2$]	131.7										131.7	n.a.
[Pd(SCN)$_4$(PPh_3)$_2$]	133.5										133.5	n.a.
[$PdCl_2$(PPh_3)$_2$]	131.5	131.6									131.55	0.05 (0.07)
[$PdBr_2$(PPh_3)$_2$]	131.6										131.6	n.a.
[PdI_2(P(C_2H_5)$_3$)$_2$]	131.4										131.4	n.a.
$Ph_2PCHCHPPh_2$	131.0	131.0									131.0	0
PPh_3	130.9	130.9	130.7	130.7	130.4	131.1	130.9	131.1	130.9	131.0	130.86	0.20 (0.21)
PCl_3	133.1	133.3									133.2	0.10 (0.14)
**Phosphorus(0)**											**Average**	**RMSD (sample standard deviation)**
P(red)	129.7	130.0										0.15 (0.21)
P(black)	130.25	129.94										0.16 (0.22)
P Not specified	130.45	129.98	129.44	130.1	129.9	129.96	130.2	129.9	130	130.45	130.04	0.25 (0.26)
**Phosphorus with Oxidation State <0 (arguably –III)**											**Average**	**RMSD (sample standard deviation)**
$Ni_{97}P_3$	129.4										129.4	n.a.
$Ni_{96}P_4$	129.3										129.3	n.a.
$Ni_{79}P_{21}$	129.4										129.4	n.a.
Zn_3P_2	128.3										128.3	n.a.
InP	127.8	127.8	127.8	128.35	128.4	129.1	129.1	129.3	129.4		128.56	0.64 (0.67)
PH_3	128.3	128.3									128.3	0

8. Computational Details

All computations were carried out with ORCA v.5.0.4.[12] Structure optimizations without constraints were conducted with tighter-than default scf ("*tightscf*") and optimization ("*tightopt*") criteria using the r²SCAN-3c composite method[13], and the PBE[14] and PBE0 functionals[15] using the RI (RIJCOSX, respectively) approximation in combination with the "Zeroth Order Regular Approximation" (ZORA) accounting for scalar relativistic effects.[16] For the latter, the ZORA-def2-SVP basis set was used for all light elements, whereas the SARC-ZORA-TZVP basis set was used for Pd.[17] Additionally, the related auxiliary basis set SARC/J[17b] and the D3-dispersion correction with Becke-Johnson (BJ) damping[18] were applied. For benchmarking purposes, the B3LYP functional[19] was further evaluated. Infrared spectra were computed from structures optimized with the r²SCAN-3c composite method. Localized orbitals (IBOs)[20] were computed using the PBE, PBE0 and r²SCAN-3c functionals as described above, as well as the TPSSh functional[21] using the parameters of the solid-state structure with optimized positions of all hydrogen atoms ("*optimizehydrogens true*"). Indicating a high degree of electron delocalization, the r²SCAN-3c method struggles to properly localize the Pd *d*-orbitals and the P *s*- and *p*-orbitals, while the other functionals struggle to localize the P *p*-orbitals. For the plotted canonical molecular orbitals, the Dip substituents were additionally truncated for clarity by replacing the aryl groups with hydrogen atoms. In case of the canonical orbitals, the structure of the cluster was constained to O_h symmetry, although the shapes of the orbitals is essentially equivalent without symmetry constraint. Absorption spectra were computed *via* TD-DFT (maxdim 5, nroots 200) with Tamm-Dancoff approximation[22] from the r²-SCAN-3c optimized structure using the PBE functional as described above. ^{31}P NMR data was computed with the PBE0 functional as described above using the r²-SCAN-3c optimized structure and Gauge-Independent Atomic Orbitals (GIAOs).[23] Triphenylphosphane was chosen as a reference for chemical shifts with a literature shift of −4.96 ppm.[24] Projections of the electron localization function (ELF)[25] in planes were plotted with Multiwfn 3.8 using the Orca-computed PBE0-wavefunctions of the solid-state structures with optimized positions of all hydrogen atoms.[26]

Table S4. Optimization benchmark for **1**. Bond lengths are given in [Å] and angles are given in [°].

	XRD	r²SCAN-3c	PBE	PBE0	B3LYP
0Pd–1Pd	2.6997	2.7322	2.7594	2.7323	2.7705
0Pd–2P	2.3317	2.3537	2.3559	2.3299	2.3513
0Pd–64P	2.3333	2.3625	2.3632	2.3353	2.3576
0Pd–127P	2.3420	2.3636	2.3620	2.3392	2.3598
1Pd–2P	2.3580	2.3675	2.3606	2.3480	2.3707
1Pd–32Pd	2.6997	2.7297	2.7565	2.7282	2.7671
1Pd–33P	2.3580	2.3683	2.3641	2.3494	2.3720
1Pd–63Pd	2.6997	2.7325	2.7580	2.7326	2.7708
1Pd–64P	2.3580	2.3641	2.3603	2.3473	2.3700
2P–3C	1.4106	1.8391	1.8383	1.8301	1.8359
2P–32Pd	2.3333	2.3571	2.3597	2.3319	2.3537
2P–157Pd	2.3420	2.3650	2.3613	2.3471	2.3654
32Pd–33P	2.3317	2.3573	2.3595	2.3359	2.3562
32Pd–158P	2.3420	2.3612	2.3625	2.3390	2.3586
33P–34C	1.8483	1.8396	1.8399	1.8309	1.8366
33P–63Pd	2.3333	2.3605	2.3616	2.3356	2.3565
33P–94Pd	2.3420	2.3636	2.3620	2.3392	2.3598
63Pd–64P	2.3317	2.3590	2.3577	2.3368	2.3569
63Pd–96P	2.3420	2.3650	2.3613	2.3471	2.3654
64P–65C	1.8483	1.8394	1.8399	1.8313	1.8369
64P–126Pd	2.3420	2.3612	2.3625	2.3390	2.3586
94Pd–95Pd	2.6997	2.7322	2.7594	2.7323	2.7705
94Pd–96P	2.3317	2.3537	2.3560	2.3299	2.3513
94Pd–158P	2.3333	2.3625	2.3632	2.3353	2.3576
95Pd–96P	2.3580	2.3674	2.3606	2.3480	2.3706
95Pd–126Pd	2.6997	2.7298	2.7565	2.7282	2.7671
95Pd–127P	2.3580	2.3683	2.3641	2.3494	2.372
95Pd–157Pd	2.6997	2.7325	2.7580	2.7326	2.7707
95Pd–158P	2.3580	2.3641	2.3603	2.3472	2.3700
96P–97C	1.8483	1.8391	1.8383	1.8301	1.8359
96P–126Pd	2.3333	2.3571	2.3597	2.3319	2.3537
126Pd–127P	2.3317	2.3573	2.3595	2.336	2.3562
127P–128C	1.8483	1.8396	1.8399	1.8309	1.8366
127P–157Pd	2.3333	2.3605	2.3616	2.3356	2.3565
157Pd–158P	2.3317	2.3590	2.3576	2.3368	2.3569
158P–159C	1.8483	1.8394	1.8399	1.8313	1.8369
RMSD		0.0748	0.0773	0.0715	0.0782
2P-0Pd-64P	110.61	109.59	108.46	109.06	108.69
2P-0Pd-127P	109.85	109.88	108.94	109.23	108.84
64P-0Pd-127P	109.79	109.64	108.79	109.03	108.64
2P-1Pd-33P	108.84	109.06	108.46	108.21	107.74
2P-1Pd-64P	108.84	109.06	108.40	108.03	107.63
33P-1Pd-64P	108.84	109.13	108.42	108.19	107.72
0Pd-2P-3C	124.67	125.05	124.21	124.22	123.88
1Pd-2P-3C	129.78	126.04	124.85	125.9	125.77
3C-2P-32Pd	126.71	126.03	124.85	125.54	125.16
3C-2P-157Pd	120.85	124.21	123.82	122.79	122.17
0Pd-2P-1Pd	70.29	70.72	71.61	71.48	71.85
0Pd-2P-157Pd	70.18	70.31	71.18	70.98	71.32
1Pd-2P-32Pd	70.26	70.59	71.46	71.32	71.71

32Pd-2P-157Pd	70.15	70.21	71.15	70.89	71.22
2P-32Pd-33P	110.61	109.78	108.64	109.22	108.84
2P-32Pd-158P	109.79	108.87	108.77	109.30	108.90
33P-32Pd-158P	109.85	109.90	108.89	109.02	108.73
1Pd-33P-34C	129.78	126.51	124.35	127.13	126.69
32Pd-33P-34C	124.67	125.17	125.08	124.02	123.67
34C-33P-63Pd	126.71	125.94	124.20	125.59	125.27
34C-33P-94Pd	120.85	123.89	124.46	121.74	121.39
1Pd-33P-32Pd	70.29	70.57	71.40	71.22	71.64
1Pd-33P-63Pd	70.26	70.60	71.41	71.36	71.74
32Pd-33P-94Pd	70.18	70.25	71.18	70.97	71.30
63Pd-33P-94Pd	70.15	70.22	71.07	71.02	71.33
33P-63Pd-64P	110.61	109.58	108.59	109.02	108.67
33P-63Pd-96P	109.79	109.59	108.78	108.76	108.48
64P-63Pd-96P	109.85	109.67	124.62	108.86	108.56
0Pd-64P-65C	126.71	125.68	124.25	124.70	124.57
1Pd-64P-65C	129.78	126.19	123.58	126.24	125.96
63Pd-64P-65C	124.67	125.22	124.85	124.79	124.24
65C-64P-126Pd	120.85	124.29	125.16	122.69	122.19
0Pd-64P-1Pd	70.26	70.63	71.49	71.40	71.75
0Pd-64P-126Pd	70.15	70.20	71.11	70.98	71.30
1Pd-64P-63Pd	70.29	70.69	71.55	71.38	71.77
63Pd-64P-126Pd	70.18	70.24	71.16	70.94	71.29
33P-94Pd-96P	109.85	109.88	108.95	109.23	108.84
33P-94Pd-158P	109.79	109.64	108.79	109.03	108.64
96P-94Pd-158P	110.61	109.59	108.46	109.06	108.69
96P-95Pd-127P	108.84	109.06	108.46	108.21	107.74
96P-95Pd-158P	108.84	109.06	108.40	108.03	107.63
127P-95Pd-158P	108.84	109.13	108.42	108.19	107.72
63Pd-96P-97C	120.85	124.21	123.82	122.78	122.17
94Pd-96P-97C	124.67	125.05	124.21	124.22	123.88
95Pd-96P-97C	129.78	126.04	124.85	125.91	125.77
97C-96P-126Pd	126.71	126.03	124.85	125.54	125.16
63Pd-96P-94Pd	70.18	70.31	71.18	70.98	71.32
63Pd-96P-126Pd	70.15	70.21	71.15	70.89	71.22
94Pd-96P-95Pd	70.29	70.72	71.61	71.48	71.85
95Pd-96P-126Pd	70.26	70.59	71.46	71.32	71.71
64P-126Pd-96P	109.79	109.87	108.77	109.30	108.90
64P-126Pd-127P	109.85	109.90	108.89	109.02	108.73
96P-126Pd-127P	110.61	109.78	108.64	109.22	108.84
0Pd-127P-128C	120.85	123.89	124.46	121.74	121.38
95Pd-127P-128C	129.78	126.51	124.35	127.13	126.69
126Pd-127P-128C	124.67	125.17	125.08	124.02	123.67
128C-127P-157Pd	126.71	125.94	124.20	125.59	125.27
0Pd-127P-126Pd	70.18	70.25	71.18	70.96	71.30
0Pd-127P-157Pd	70.15	70.22	71.07	71.02	71.33
95Pd-127P-126Pd	70.29	70.57	71.40	71.22	71.64
95Pd-127P-157Pd	70.26	70.60	71.41	71.36	71.74
2P-157Pd-127P	109.79	109.59	108.78	108.76	108.48
2P-157Pd-158P	109.85	109.67	108.89	108.86	108.56
127P-157Pd-158P	110.61	109.58	108.59	109.02	108.67
32Pd-158P-159C	120.85	124.29	125.16	122.69	122.19
94Pd-158P-159C	129.78	125.68	124.25	124.70	124.57
95Pd-158P-159C	120.85	126.19	123.58	126.24	125.96
157Pd-158P-159C	124.67	125.22	124.85	124.79	124.24
32Pd-158P-94Pd	70.15	70.20	71.11	70.98	71.30
32Pd-158P-157Pd	70.18	70.24	71.16	70.94	71.29
94Pd-158P-95Pd	70.26	70.63	71.49	71.40	71.75
95Pd-158P-157Pd	70.29	70.69	71.55	71.38	71.77
RMSD		1.61	2.83	1.60	1.79

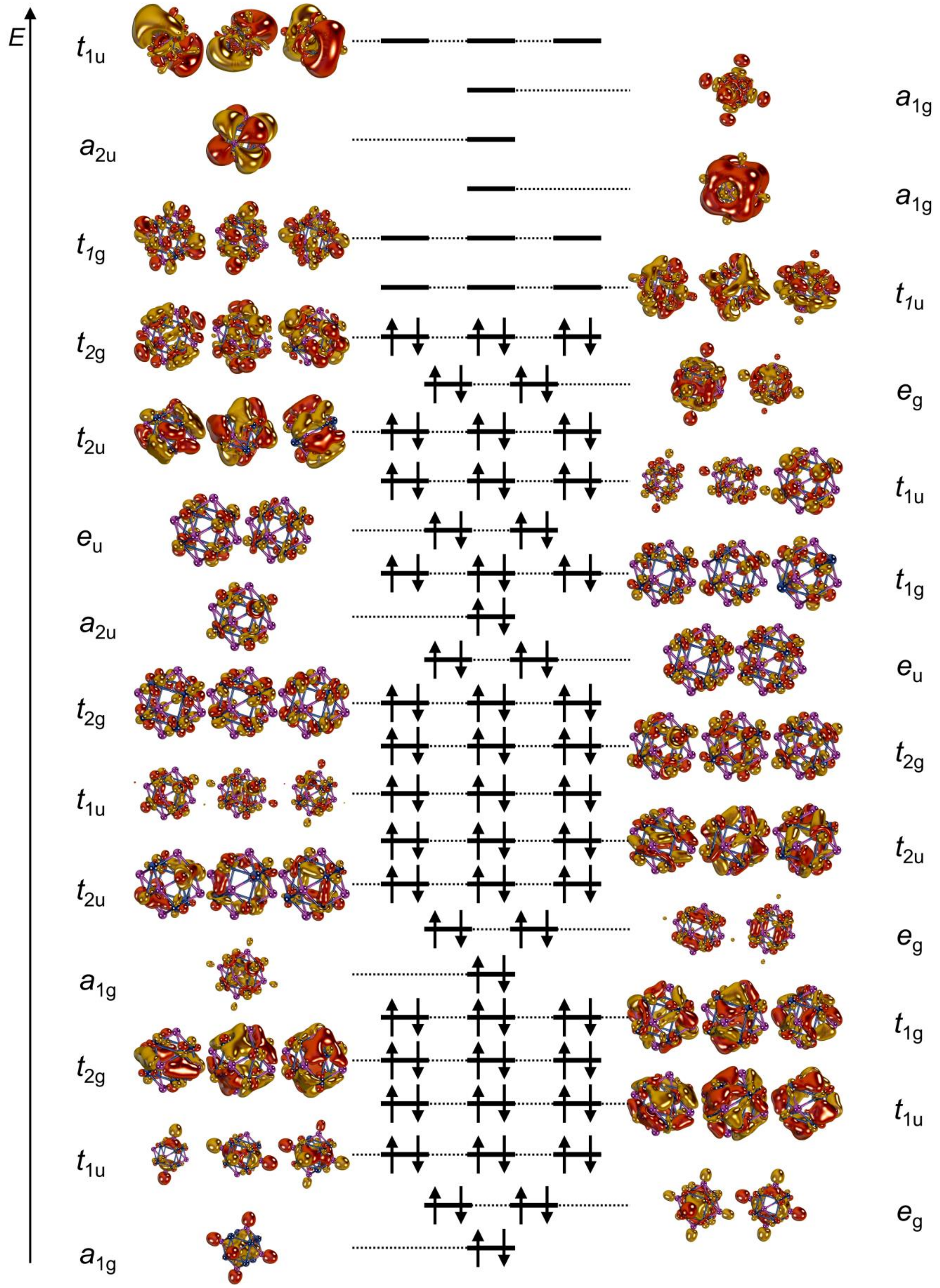

Figure S37. MO diagram for the 104 cluster electrons in **1** (PBE0/def2-TZVPP//HOpt).

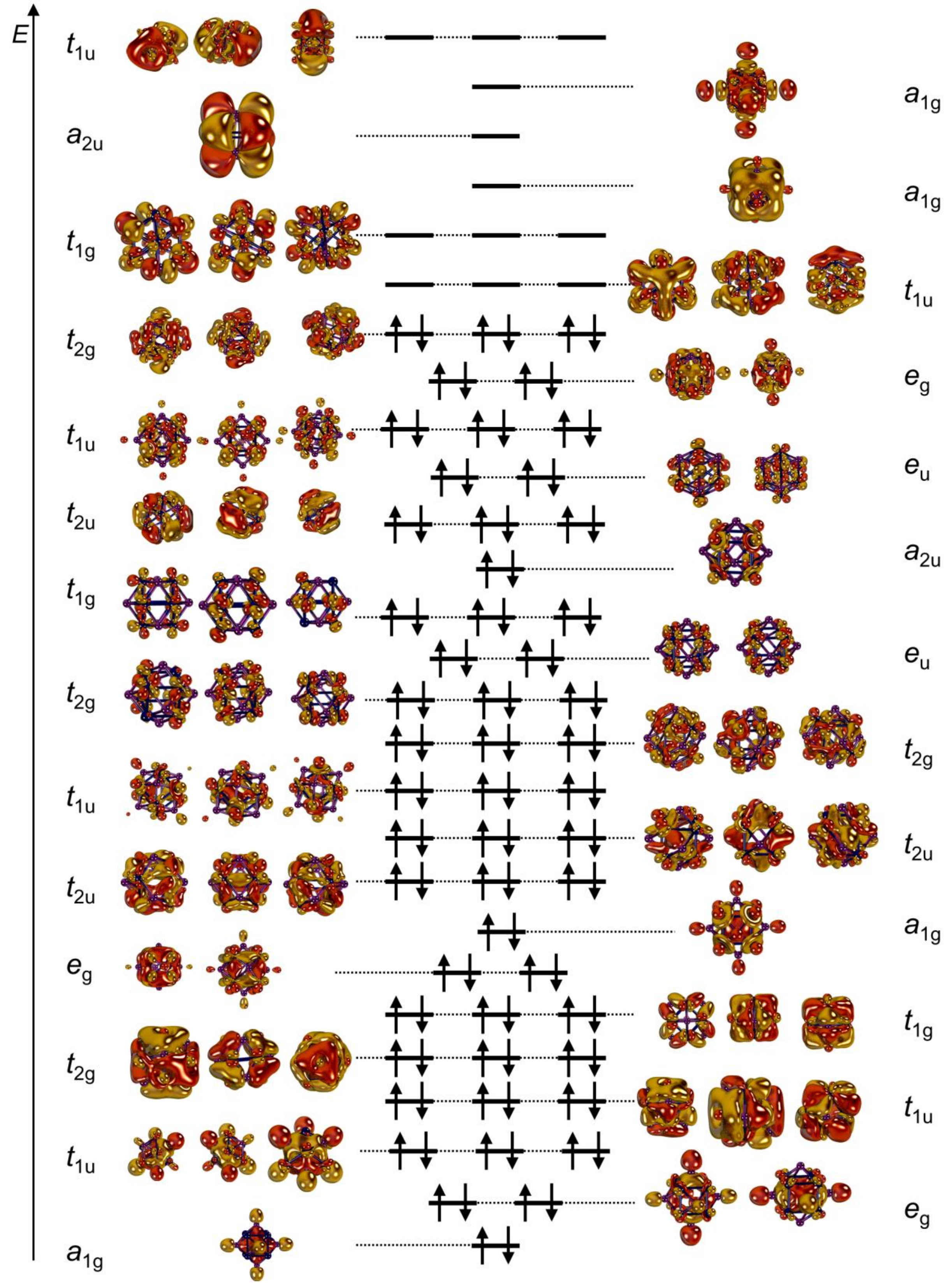

Figure S38. MO diagram for the 104 cluster electrons in **1** (PBE/def2-TZVPP//HOpt).

Table S5. Selected IBOs for **1**. Aryl substituents are truncated by hydrogen atoms for clarity, yet were included in the calculations. All orbitals were plotted with an isovalue of 0.70 (PBE/def2-TZVPP//HOpt, TPSSh/def2-TZVPP//HOpt, PBE0/def2-TZVPP//HOpt). Composite method r²SCAN-3c//HOpt fails to properly localize.

	**PBE**	**TPSSh**	**PBE0**
**Pd** $\boldsymbol{d_{x^2-y^2}}$	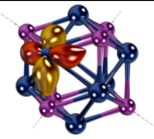 Pd 0.80	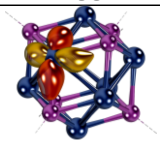 Pd 0.79	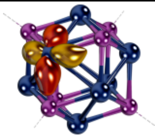 Pd 0.80
**Pd** $\boldsymbol{d_{xy}}$	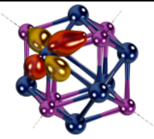 Pd 0.79	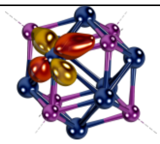 Pd 0.79	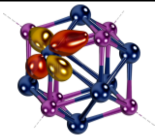 Pd 0.80
**Pd** $\boldsymbol{d_{xz}}$	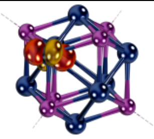 Pd 0.95	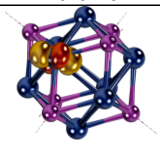 Pd 0.96	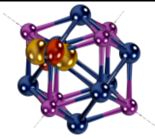 Pd 0.96
**Pd** $\boldsymbol{d_{yz}}$	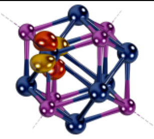 Pd 0.96	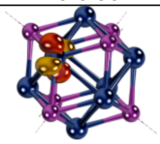 Pd 0.96	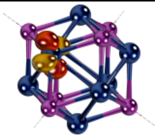 Pd 0.96
**Pd** $\boldsymbol{d_{z^2}}$	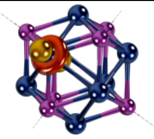 Pd 0.95	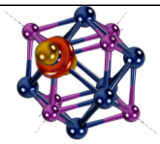 Pd 0.95	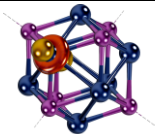 Pd 0.96
**P *s***	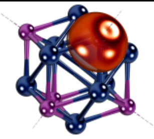 P 0.85	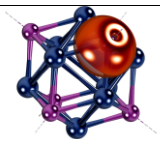 P 0.85	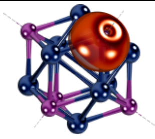 P 0.85
**P *p* deloc.**	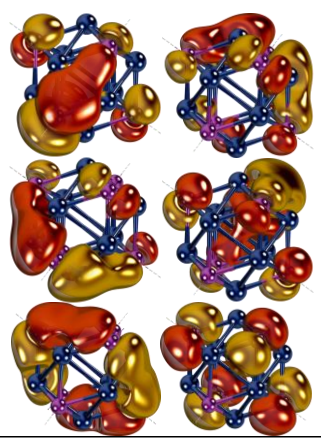	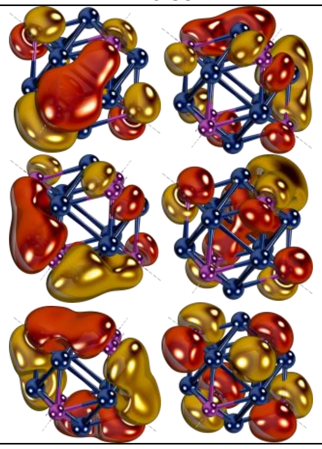	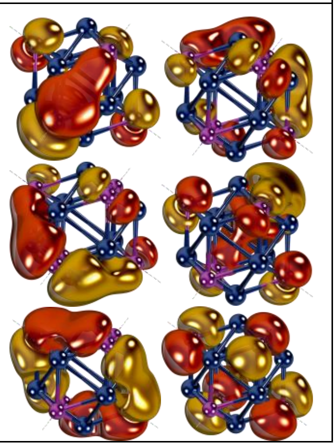

Table S6. Selected IBOs for **4**. Aryl substituents are truncated by hydrogen atoms for clarity, yet were included in the calculations. All orbitals were plotted with an isovalue of 0.70 (PBE/def2-TZVPP//HOpt, TPSSh/def2-TZVPP//HOpt, PBE0/def2-TZVPP//HOpt). For Pd orbitals, the IBOs for Pd with and without isocyanide ligand are shown. Composite method r²SCAN-3c//HOpt fails to properly localize.

	PBE	TPSSh	PBE0
**Pd *d*$_{x^2-y^2}$**	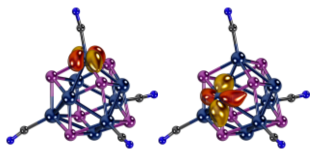 Pd 0.86 Pd 0.81	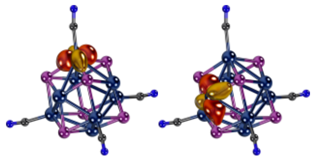 Pd 0.86 Pd 0.80	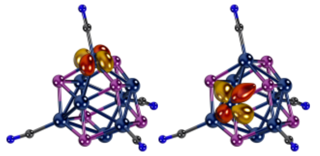 Pd 0.86 Pd 0.81
**Pd *d*$_{xy}$**	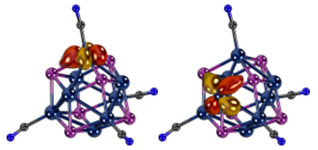 Pd 0.85 Pd 0.80	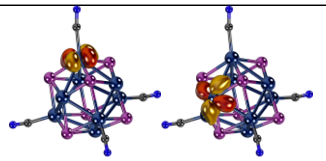 Pd 0.86 Pd 0.80	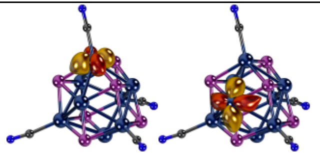 Pd 0.87 Pd 0.81
**Pd *d*$_{xz}$**	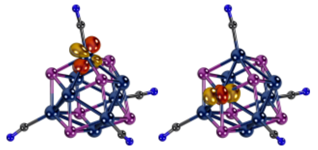 Pd 0.95 Pd 0.97	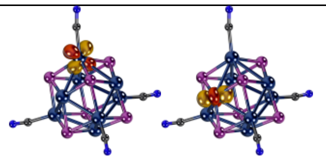 Pd 0.95 Pd 0.98	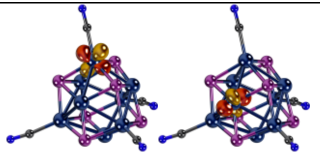 Pd 0.96 Pd 0.98
**Pd *d*$_{yz}$**	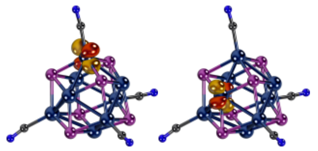 Pd 0.95 Pd 0.97	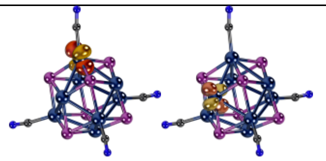 Pd 0.95 Pd 0.98	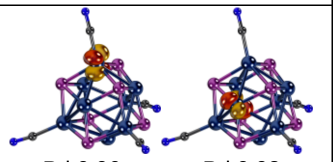 Pd 0.96 Pd 0.98
**Pd *d*$_{z^2}$**	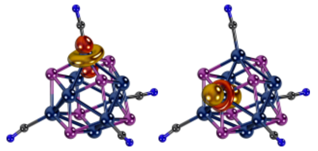 Pd 0.90 Pd 0.98	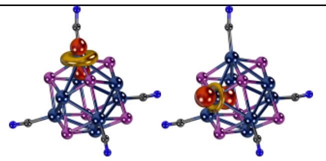 Pd 0.90 Pd 0.98	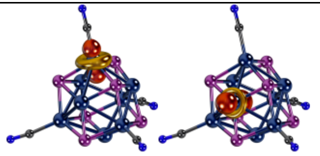 Pd 0.91 Pd 0.99
**P *s***	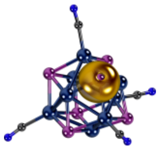 P 0.92	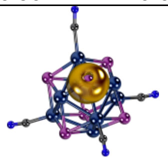 P 0.93	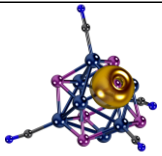 P 0.93
**P *p* deloc.**	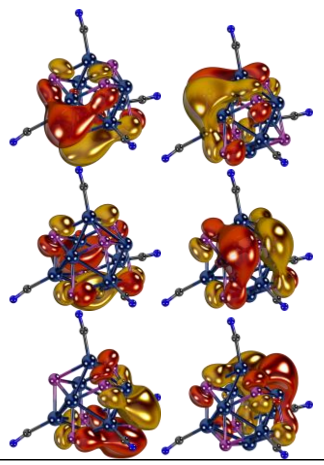	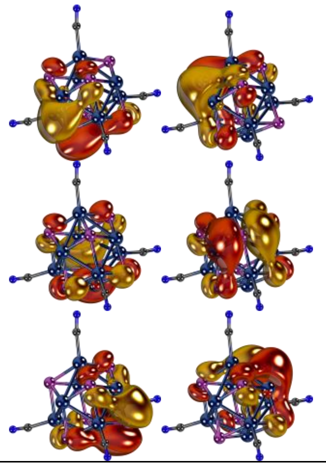	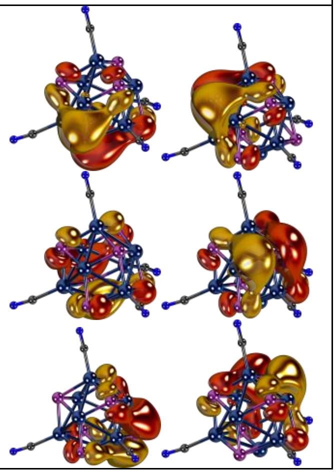

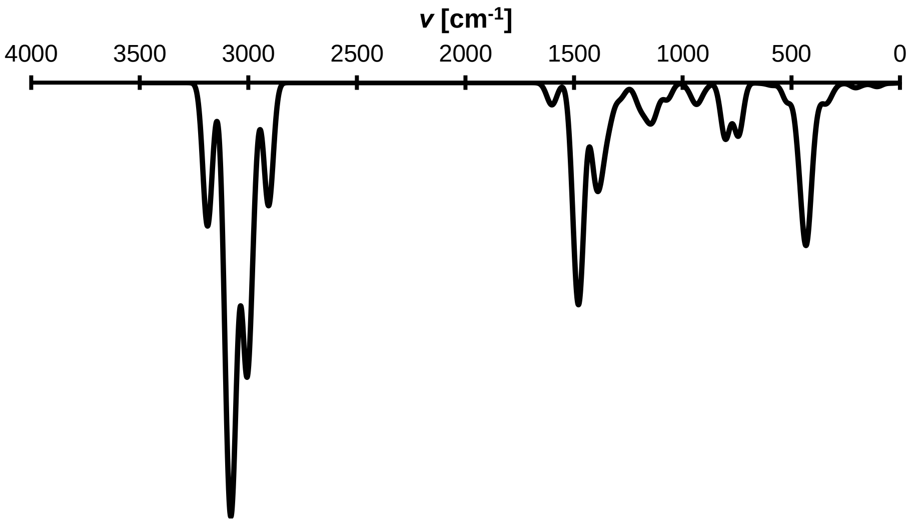

Figure S39. Computed IR absorption spectrum for **1** (r^2SCAN-3c; Gaussian broadening with a band width of 50 at ½ height).

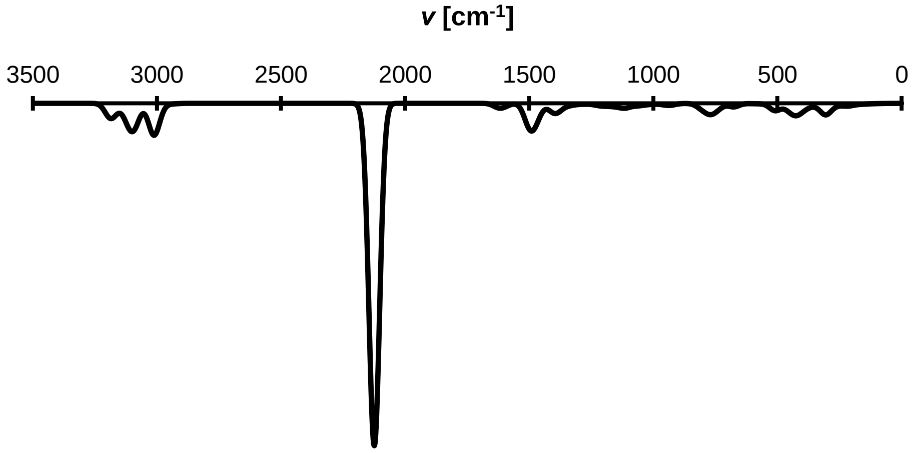

Figure S40. Computed IR absorption spectrum for **4** (r^2SCAN-3c; Gaussian broadening with a band width of 50 at ½ height).

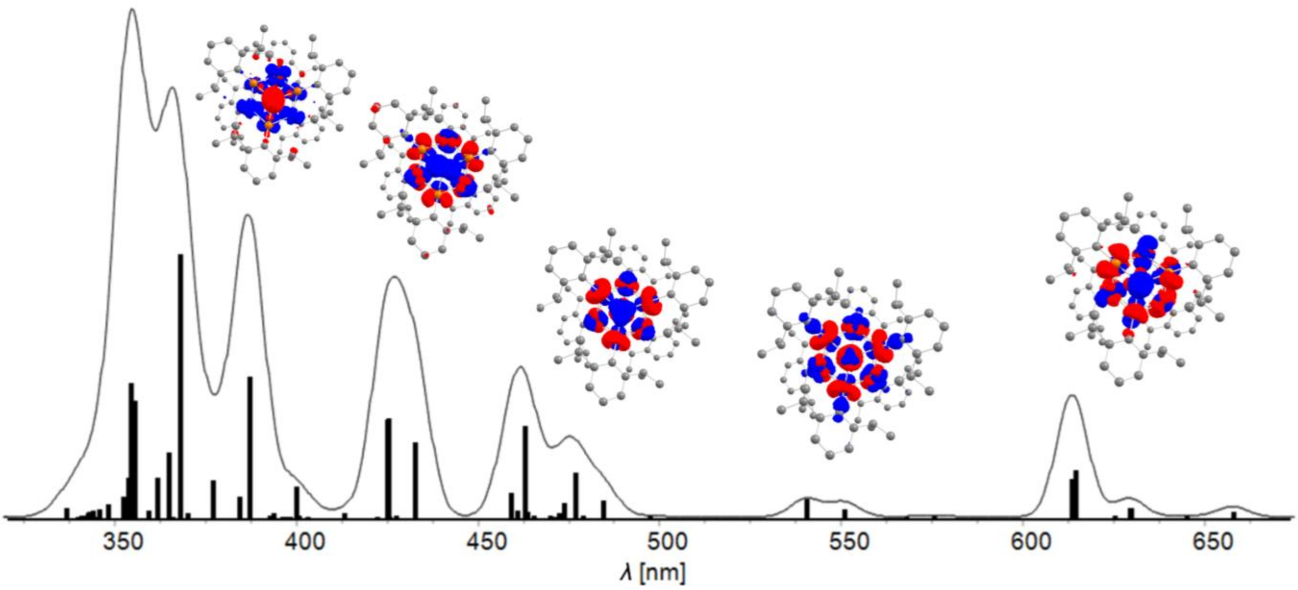

Figure S41. UV-Vis electronic absorption spectrum and transition difference densities for **1** as obtained at the TD-DFT ZORA-PBE/def2-TZVPP//HOpt level of theory (200 roots).

Table S7. Computed ^{31}P NMR parameters for **1** and **4** at the PBE0/def2-TZVPP//r²SCAN-3c level. For the "average" data, the isotropic shift and anisotropy are calculated from the averaged tensor elements. The calculations struggle to accurately reproduce the experimental values and suffer from symmetry breaking, yet indicate a phosphinidene electronic structure.

Compound	Atom	δ_{11} [ppm]	δ_{22} [ppm]	δ_{33} [ppm]	δ_{iso} [ppm]	$\delta_{iso,ref}$ [ppm]	Anisotropy [ppm]
**1**	2P	−517.13	+890.54	−1753.31	−459.96	+771.31	−1940.02
	33P	−350.40	+2561.52	−6094.89	−1294.59	+1605.93	−7200.45
	64P	+26.118	−2558.99	−1435.68	−1222.85	+1534.20	−319.25
	96P	−317.17	−938.78	+4380.19	+1041.42	−730.07	+5008.17
	127P	−175.31	−380.54	+565.36	+3.17	+308.18	+843.28
	158P	−275.71	−513.48	−918.29	−569.16	+880.51	−523.70
	average	−268.27	−156.62	−876.10	−433.66	+745.01	−663.66
**4**	2P	+241.49	−3589.39	−2606.15	−1984.68	+2296.03	−932.20
	33P	+266.21	−892.18	−2836.31	−1154.09	+1465.44	−2523.32
	64P	−10.53	−809.03	+2251.49	+477.31	−165.97	+2661.28
	96P	−133.76	−225.57	−1258.16	−539.16	+850.51	−1078.49
	127P	−4.70	−342.88	+5992.93	+1881.79	−1570.44	+6166.72
	158P	+828.54	+1515.74	+2964.92	+1769.73	−1458.39	+1792.78
	average	+197.88	−723.89	+751.457	+75.15	+236.20	+1014.46
**PPh_3**	33P	+299.49	+312.91	+336.52	+316.31	−4.96	+30.32

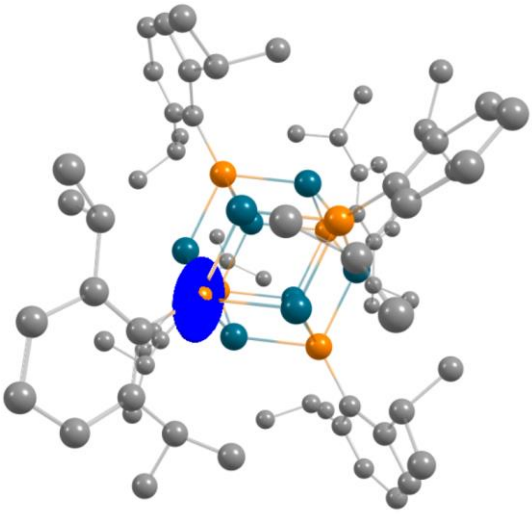

Figure S42. Visualization of the computed (PBE0/def2-TZVPP// r²SCAN-3c) representative ^{31}P NMR tensor for 158P in **1**. Hydrogen atoms are omitted for clarity.

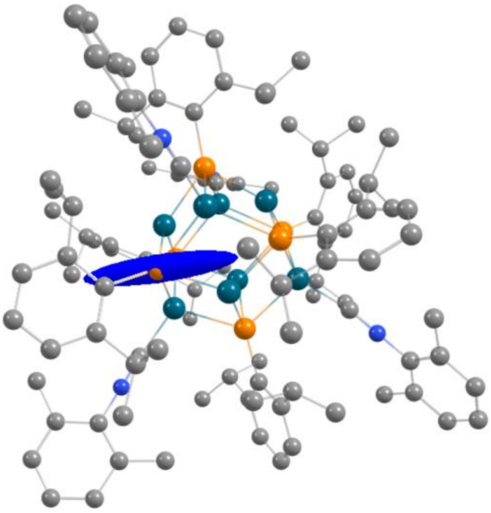

Figure S43. Visualization of the computed (PBE0/def2-TZVPP//r²SCAN-3c) representative ^{31}P NMR tensor for 96P in **4**. Hydrogen atoms are omitted for clarity.

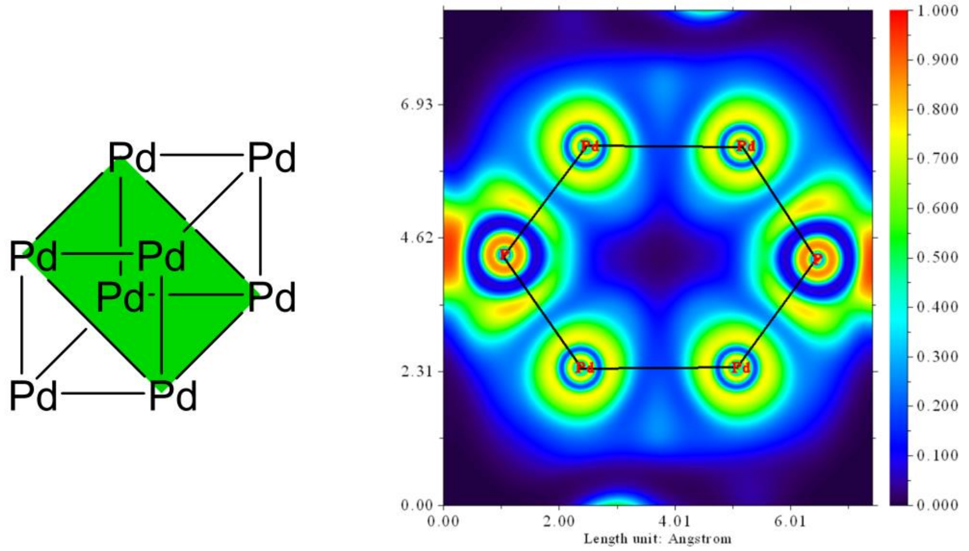

Figure S44. ELF plot for **1** in the diagonal plane (PBE/def2-TZVPP//Hopt).

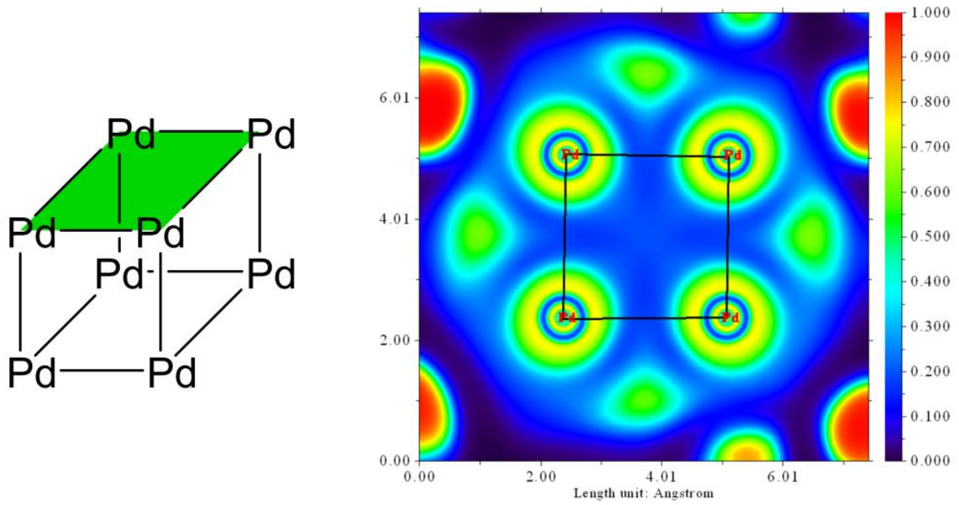

Figure S45. ELF plot for **1** on the face of the Pd_8 cube (PBE/def2-TZVPP//Hopt).

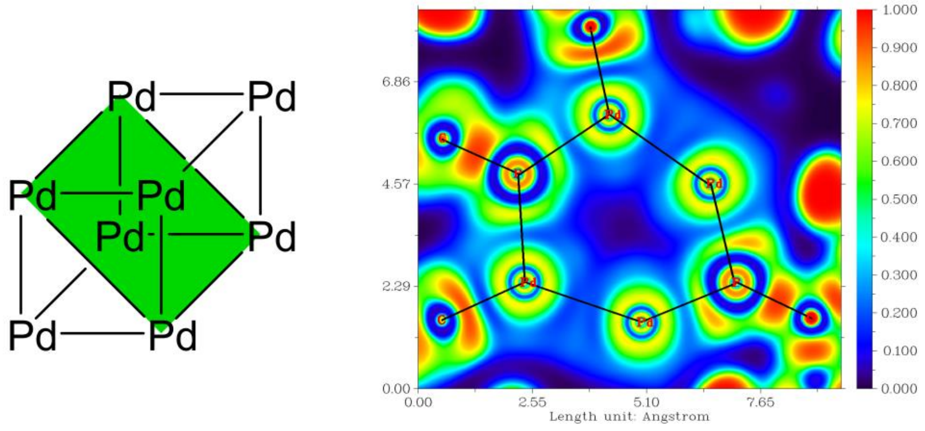

Figure S46. ELF plot for **4** in the diagonal plane of the Pd_8 cube (PBE/def2-TZVPP//Hopt).

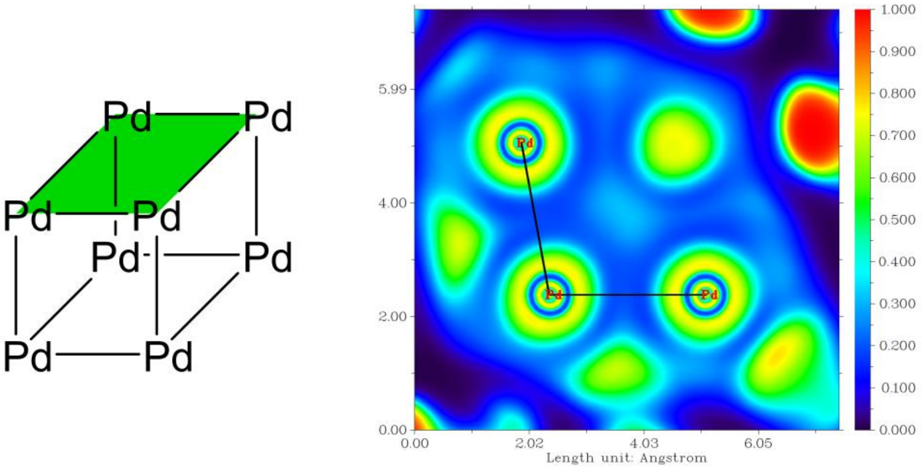

Figure S47. ELF plot for **4** on the face of the distorted Pd_8 cube (PBE/def2-TZVPP//Hopt).

Table S8. Topological analysis of the electronic structure of **1** and **4**. CCP, cage critical point; BCP, bond critical point.

	**1**		**4**	
	**ELF (Electron localization function)**	**Electron density in [e$^-$ au^{-3}]**	**ELF (Electron localization function)**	**Electron density in [e$^-$ au^{-3}]**
**CCP (+3,+3)**	0.017	0.005	0.028	0.007
**BCP (Pd–Pd) (+3,−1)**	0.184	0.050	0.195	0.046
**BCP (Pd^{naked}–P) (+3,−1)**	0.430	0.089	0.446	0.098
**BCP (Pd^{CNXyl}–Pd) (+3,−1)**	–	–	0.369	0.072

XYZ Coordinates

188

Pd8(PDip)6 (HOpt)

Pd	-1.894520	13.030426	2.524843
Pd	-0.000039	14.116742	4.112003
P	-0.007564	11.902323	3.301720
C	-0.045796	10.303779	4.228795
C	-1.243827	9.892749	4.849775
C	-1.266834	8.628572	5.450884
H	-2.178450	8.294207	5.941268
C	-0.170057	7.800821	5.440423
H	-0.218399	6.818085	5.907567
C	0.987630	8.218196	4.816306
H	1.850134	7.554403	4.805561
C	1.092548	9.476035	4.229834
C	2.414069	9.825680	3.569126
H	2.452812	10.925079	3.471523
C	2.511993	9.249766	2.154607
H	2.497776	8.152155	2.178795
H	3.436778	9.577709	1.663949
H	1.670500	9.589167	1.538910
C	3.622014	9.410845	4.404453
H	3.552390	9.787270	5.431893
H	4.539119	9.809913	3.954208
H	3.739875	8.321323	4.452027
C	-2.482432	10.779763	4.949101
H	-2.534213	11.369879	4.015537
C	-2.353033	11.762288	6.104359
H	-2.299091	11.229746	7.062688
H	-3.212751	12.443159	6.133052
H	-1.450103	12.377878	6.003961
C	-3.794088	9.998159	5.058841
H	-3.884032	9.240447	4.271629
H	-4.640305	10.687646	4.966275
H	-3.893466	9.498202	6.030469
Pd	1.888052	13.019252	2.524885
P	1.921509	15.217436	3.301820
C	3.324970	15.983574	4.228968
C	4.279926	15.151547	4.849962
C	5.386215	15.763695	5.451128
H	6.131957	15.141161	5.940704
C	5.554681	17.127408	5.440708
H	6.430094	17.576747	5.907746
C	4.614403	17.921323	4.816576
H	4.758047	19.000080	4.803633
C	3.472646	17.383280	4.230047
C	2.509108	18.352946	3.569328
H	1.538362	17.835843	3.470615
C	2.958956	18.725743	2.154835
H	3.912496	19.269809	2.179447
H	2.209608	19.355510	1.659486
H	3.096078	17.826566	1.542155
C	2.264362	19.606453	4.404678
H	1.976442	19.358290	5.433067
H	1.457614	20.198192	3.955263
H	3.147519	20.255426	4.448835
C	4.131047	13.635374	4.949243
H	3.645482	13.294388	4.016337
C	3.215413	13.256145	6.104457
H	3.652775	13.565954	7.062504
H	3.053649	12.171265	6.132270
H	2.231252	13.731934	6.008780
C	5.463760	12.890246	5.059014
H	6.165291	13.194422	4.273421
H	5.290861	11.812879	4.961718
H	5.945806	13.050120	6.031723
Pd	0.006442	16.300642	2.524942
P	-1.913999	15.230506	3.301777
C	-3.279259	16.062845	4.228895
C	-3.036204	17.305858	4.849930
C	-4.119508	17.957844	5.451072
H	-3.953270	18.914685	5.941058
C	-5.384750	17.421883	5.440591
H	-6.211710	17.955103	5.907679
C	-5.602138	16.210638	4.816420
H	-6.608273	15.796000	4.803703
C	-4.565279	15.490883	4.229912
C	-4.923240	14.171620	3.569146
H	-3.991109	13.587354	3.472680
C	-5.470962	14.374836	2.154637
H	-6.419574	14.927359	2.179822
H	-5.641922	13.410847	1.659419
H	-4.761240	14.942620	1.541157
C	-5.886467	13.332888	4.404438
H	-5.527111	13.205947	5.432471
H	-5.998789	12.339096	3.954238
H	-6.889509	13.774411	4.450255
C	-1.648724	17.935010	4.949279
H	-1.110782	17.685062	4.016098
C	-0.862529	17.331632	6.104507
H	-1.339103	17.571870	7.063795
H	0.163964	17.718359	6.119787
H	-0.800140	16.239486	6.019224
C	-1.669785	19.461735	5.059088
H	-2.280173	19.917500	4.270767
H	-0.649303	19.849285	4.966666
H	-2.054205	19.798276	6.030120
Pd	1.894520	15.203157	0.960023
Pd	0.000039	14.116840	-0.627137
P	0.007564	16.331260	0.183147
C	0.045796	17.929804	-0.743929
C	1.243827	18.340833	-1.364908
C	1.266834	19.605011	-1.966018
H	2.178451	19.939375	-2.456402
C	0.170057	20.432762	-1.955556
H	0.218399	21.415497	-2.422700
C	-0.987630	20.015387	-1.331440
H	-1.850134	20.679179	-1.320696
C	-1.092548	18.757548	-0.744967
C	-2.414069	18.407903	-0.084260
H	-2.452811	17.308504	0.013342
C	-2.511993	18.983817	1.330260
H	-2.497791	20.081428	1.306070
H	-3.436770	18.655862	1.820924
H	-1.670491	18.644429	1.945952
C	-3.622014	18.822738	-0.919586
H	-3.552392	18.446312	-1.947025
H	-4.539119	18.423671	-0.469340
H	-3.739874	19.912260	-0.967160
C	2.482432	17.453820	-1.464234
H	2.534214	16.863706	-0.530669
C	2.353033	16.471294	-2.619492
H	2.299092	17.003836	-3.577821
H	3.212751	15.790422	-2.648185
H	1.450102	15.855705	-2.519094
C	3.794088	18.235423	-1.573974
H	3.884032	18.993136	-0.786763
H	4.640305	17.545935	-1.481408
H	3.893467	18.735379	-2.545602
Pd	-1.888052	15.214331	0.959981
P	-1.921509	13.016147	0.183047
C	-3.324970	12.250009	-0.744101
C	-4.279926	13.082035	-1.365095
C	-5.386215	12.469888	-1.966262
H	-6.131957	13.092421	-2.455839
C	-5.554681	11.106174	-1.955841
H	-6.430094	10.656835	-2.422880
C	-4.614403	10.312260	-1.331710
H	-4.758047	9.233503	-1.318767
C	-3.472646	10.850303	-0.745180
C	-2.509108	9.880637	-0.084462

H	-1.538363	10.397741	0.014251
C	-2.958956	9.507840	1.330032
H	-3.912496	8.963774	1.305420
H	-2.209608	8.878074	1.825382
H	-3.096079	10.407018	1.942710
C	-2.264362	8.627130	-0.919811
H	-1.976443	8.875293	-1.948201
H	-1.457614	8.035391	-0.470396
H	-3.147519	7.978157	-0.963967
C	-4.131047	14.598209	-1.464376
H	-3.645483	14.939194	-0.531469
C	-3.215412	14.977438	-2.619590
H	-3.652775	14.667630	-3.577638
H	-3.053647	16.062318	-2.647402
H	-2.231252	14.501648	-2.523913
C	-5.463760	15.343336	-1.574147
H	-6.165291	15.039159	-0.788554
H	-5.290862	16.420703	-1.476851
H	-5.945807	15.183462	-2.546856
Pd	-0.006442	11.932941	0.959924
P	1.913999	13.003077	0.183090
C	3.279259	12.170738	-0.744028
C	3.036204	10.927724	-1.365063
C	4.119508	10.275739	-1.966206
H	3.953270	9.318898	-2.456192
C	5.384750	10.811700	-1.955724
H	6.211710	10.278480	-2.422813
C	5.602137	12.022945	-1.331554
H	6.608273	12.437583	-1.318838
C	4.565279	12.742700	-0.745045
C	4.923240	14.061963	-0.084280
H	3.991108	14.646228	0.012185
C	5.470962	13.858747	1.330230
H	6.419571	13.306219	1.305046
H	5.641927	14.822736	1.825445
H	4.761237	13.290969	1.943712
C	5.886466	14.900695	-0.919571
H	5.527112	15.027635	-1.947605
H	5.998788	15.894487	-0.469372
H	6.889509	14.459173	-0.965386
C	1.648724	10.298573	-1.464412
H	1.110784	10.548520	-0.531230
C	0.862529	10.901951	-2.619640
H	1.339096	10.661699	-3.578928
H	-0.163969	10.515235	-2.634912
H	0.800152	11.994098	-2.534368
C	1.669785	8.771848	-1.574221
H	2.280178	8.316084	-0.785903
H	0.649304	8.384297	-1.481794
H	2.054202	8.435308	-2.545255

20

Pd8(PDip)6 (HOpt) trunc

Pd	-1.899907	13.039877	2.524647
Pd	-0.000155	14.117128	4.111686
P	-0.018565	11.902626	3.301803
H	-0.025667	10.722003	4.104857
Pd	1.882564	13.010056	2.524841
P	1.926827	15.208189	3.301379
H	2.952860	15.792183	4.104642
Pd	0.017154	16.300682	2.524229
P	-1.908570	15.240167	3.301182
H	-2.927543	15.836260	4.104546
Pd	1.899860	15.193620	0.959585
Pd	0.000107	14.116368	-0.627454
P	0.018518	16.330871	0.182429
H	0.025619	17.511495	-0.620623
Pd	-1.882611	15.223441	0.959389
P	-1.926875	13.025307	0.182853
H	-2.952906	12.441312	-0.620410
Pd	-0.017202	11.932815	0.960002
P	1.908522	12.993330	0.183051
H	2.927495	12.397237	-0.620315

20

Pd8(PDip)6 (HOpt) trunc, *Oh* symmetry

Pd	-1.346520	-1.346520	-1.346520
Pd	-1.346520	1.346520	-1.346520
P	-2.708371	0.000000	0.000000
H	-4.136057	0.000000	0.000000
Pd	-1.346520	1.346520	1.346520
P	0.000000	2.708371	0.000000
H	0.000000	4.136057	0.000000
Pd	1.346520	1.346520	-1.346520
P	0.000000	0.000000	-2.708371
H	0.000000	0.000000	-4.136057
Pd	1.346520	1.346520	1.346520
Pd	1.346520	-1.346520	1.346520
P	2.708371	0.000000	0.000000
H	4.136057	0.000000	0.000000
Pd	1.346520	-1.346520	-1.346520
P	0.000000	-2.708371	0.000000
H	0.000000	-4.136057	0.000000
Pd	-1.346520	-1.346520	1.346520
P	0.000000	0.000000	2.708371
H	0.000000	0.000000	4.136057

188

Pd8(PDip)6 (r²SCAN-3c)

Pd	-1.902734	12.976065	2.525180
Pd	-0.006440	14.121490	4.124199
P	0.029297	11.894102	3.322998
C	0.037310	10.372595	4.356075
C	-1.089268	10.056197	5.161029
C	-1.057478	8.895010	5.932578
H	-1.910824	8.642970	6.553662
C	0.043601	8.052349	5.920692
H	0.046825	7.152222	6.529311
C	1.140170	8.359889	5.129753
H	1.995195	7.691373	5.127857
C	1.165388	9.510150	4.341582
C	2.376638	9.760442	3.458707
H	2.464811	10.851060	3.313964
C	2.169494	9.131503	2.073837
H	2.100147	8.040267	2.157969
H	3.004181	9.381489	1.408024
H	1.245637	9.498577	1.607661
C	3.701315	9.294069	4.068605
H	3.815537	9.649732	5.098035
H	4.534032	9.685680	3.474334
H	3.794623	8.202506	4.072746
C	-2.308365	10.958603	5.251771
H	-2.406113	11.483463	4.285596
C	-2.107768	12.032279	6.329587
H	-2.038720	11.570034	7.321426
H	-2.945165	12.740537	6.327830
H	-1.186553	12.602286	6.152915
C	-3.625245	10.209272	5.469857
H	-3.742126	9.387977	4.754957
H	-4.463027	10.900849	5.334479
H	-3.707188	9.798952	6.482431
Pd	1.929434	13.042028	2.530880
P	1.908311	15.262136	3.323199
C	3.228667	16.035185	4.344473
C	4.084557	15.220737	5.133617
C	5.080227	15.833316	5.893949
H	5.740754	15.222645	6.500731
C	5.247331	17.209807	5.887921
H	6.029749	17.665831	6.488342
C	4.415615	18.002582	5.112159
H	4.557791	19.078456	5.112774
C	3.401366	17.444982	4.333908

C	2.564904	18.368051	3.463272	H	-4.534015	18.548047	0.009799
H	1.574938	17.897067	3.332303	H	-3.794447	20.031146	-0.588608
C	3.194280	18.505047	2.069358	C	2.308545	17.274870	-1.766788
H	4.177162	18.986235	2.140563	H	2.406162	16.749962	-0.800624
H	2.553271	19.107541	1.414705	C	2.108000	16.201259	-2.844680
H	3.326137	17.522086	1.598678	H	2.039075	16.663567	-3.836499
C	2.312265	19.747786	4.077079	H	2.945364	15.492962	-2.842879
H	1.968565	19.668373	5.113787	H	1.186740	15.631283	-2.668135
H	1.542383	20.268652	3.497530	C	3.625493	18.024132	-1.984699
H	3.207367	20.379560	4.062622	H	3.742372	18.845353	-1.269713
C	3.930617	13.711043	5.212439	H	4.463213	17.332484	-1.849315
H	3.531848	13.372409	4.240132	H	3.707548	18.434538	-2.997229
C	2.904816	13.324227	6.285855	Pd	-1.929415	15.191563	0.953934
H	3.264609	13.614746	7.279981	P	-1.908297	12.971453	0.161623
H	2.728662	12.241664	6.277805	C	-3.228704	12.198349	-0.859549
H	1.942452	13.821035	6.109467	C	-4.084852	13.012698	-1.648546
C	5.246875	12.958560	5.424248	C	-5.080730	12.400002	-2.408509
H	6.014869	13.286305	4.716024	H	-5.741471	13.010591	-3.015146
H	5.081065	11.886845	5.273939	C	-5.247795	11.023509	-2.402280
H	5.637588	13.083641	6.440146	H	-6.030375	10.567398	-3.002425
Pd	-0.035832	16.338210	2.526813	C	-4.415828	10.230842	-1.626680
P	-1.947157	15.202167	3.315004	H	-4.557953	9.154963	-1.627157
C	-3.279704	15.951274	4.338084	C	-3.401350	10.788557	-0.848809
C	-2.999454	17.082043	5.150879	C	-2.564711	9.865655	0.021833
C	-4.029732	17.632610	5.912653	H	-1.574764	10.336735	0.152611
H	-3.828157	18.495897	6.538258	C	-3.193884	9.728798	1.415853
C	-5.310396	17.101480	5.884495	H	-4.176748	9.247545	1.344851
H	-6.098031	17.547204	6.485810	H	-2.552748	9.126422	2.070492
C	-5.583382	16.001284	5.086380	H	-3.325725	10.711810	1.886431
H	-6.589393	15.594233	5.070126	C	-2.312062	8.485859	-0.591836
C	-4.590795	15.406729	4.307449	H	-1.968523	8.565166	-1.628605
C	-4.971800	14.232997	3.420679	H	-1.542058	7.965120	-0.012336
H	-4.064963	13.622534	3.270042	H	-3.207117	7.854024	-0.577167
C	-5.422078	14.724340	2.038306	C	-3.931050	14.522390	-1.727612
H	-6.332889	15.328667	2.126758	H	-3.531821	14.861175	-0.755544
H	-5.626251	13.873964	1.376965	C	-2.905859	14.909160	-2.801627
H	-4.645905	15.340719	1.565585	H	-3.266221	14.618589	-3.795532
C	-6.025174	13.305753	4.033598	H	-2.729703	15.991724	-2.793732
H	-5.771175	13.038810	5.064757	H	-1.943395	14.412361	-2.625769
H	-6.087588	12.384923	3.443638	C	-5.247474	15.274740	-1.938837
H	-7.024071	13.755864	4.035032	H	-6.015054	14.947071	-1.230128
C	-1.610094	17.688410	5.256043	H	-5.081656	16.346495	-1.788822
H	-1.105746	17.527008	4.287366	H	-5.638738	15.149435	-2.954497
C	-0.780189	16.966867	6.326556	Pd	0.035853	11.895371	0.958004
H	-1.222190	17.117263	7.318606	P	1.947186	13.031416	0.169809
H	0.247946	17.348524	6.337613	C	3.279782	12.282337	-0.853241
H	-0.733891	15.887507	6.132119	C	2.999470	11.151808	-1.666356
C	-1.604308	19.200318	5.497031	C	4.029775	10.601225	-2.428075
H	-2.253284	19.722384	4.786042	H	3.828176	9.738062	-3.053844
H	-0.585953	19.582468	5.373079	C	5.310519	11.132150	-2.399625
H	-1.924614	19.460221	6.512015	H	6.098196	10.686356	-3.000833
Pd	1.902764	15.257521	0.959643	C	5.583555	12.232145	-1.601260
Pd	0.006465	14.112092	-0.639381	H	6.589649	12.638984	-1.584700
P	-0.029266	16.339479	0.161818	C	4.590971	12.826636	-0.822274
C	-0.037164	17.860977	-0.871281	C	4.972042	14.000246	0.064620
C	1.089498	18.177348	-1.676122	H	4.065277	14.610824	0.215230
C	1.057816	19.338540	-2.447668	C	5.422122	13.508717	1.446987
H	1.911221	19.590544	-3.068685	H	6.332882	12.904308	1.358578
C	-0.043248	20.181222	-2.435908	H	5.626299	14.359003	2.108444
H	-0.046393	21.081338	-3.044544	H	4.645834	12.892358	1.919543
C	-1.139913	19.873700	-1.645097	C	6.025614	14.927364	-0.548145
H	-1.994936	20.542223	-1.643307	H	5.771736	15.194420	-1.579304
C	-1.165234	18.723445	-0.856919	H	6.088134	15.848142	0.041885
C	-2.376627	18.473190	0.025767	H	7.024438	14.477088	-0.549540
H	-2.464853	17.382583	0.170529	C	1.610036	10.545683	-1.771836
C	-2.169700	19.102173	1.410649	H	1.105616	10.706883	-0.803160
H	-2.100335	20.193406	1.326494	C	0.780360	11.267673	-2.842227
H	-3.004492	18.852211	2.076338	H	1.222414	11.117452	-3.834279
H	-1.245916	18.735111	1.876980	H	-0.247850	10.886226	-2.853465
C	-3.701188	18.939578	-0.584371	H	0.734265	12.346992	-2.647513
H	-3.815263	18.583845	-1.613792	C	1.604059	9.033850	-2.013280

H	2.252899	8.511478	-1.302391
H	0.585644	8.651801	-1.889538
H	1.924423	8.774218	-3.028315

188

Pd8(PDip)6 (PBE)

Pd	-1.925575	12.965354	2.537712
Pd	-0.003686	14.130320	4.138862
P	0.031536	11.915274	3.323548
C	0.044605	10.408361	4.376433
C	-1.079965	10.118311	5.209650
C	-1.039952	8.960180	6.007549
H	-1.892934	8.722876	6.653691
C	0.066846	8.104293	5.992220
H	0.076192	7.206995	6.623529
C	1.161491	8.393826	5.169813
H	2.022238	7.714962	5.164245
C	1.178552	9.538303	4.351977
C	2.374214	9.781011	3.436935
H	2.440167	10.883868	3.271902
C	2.143409	9.144713	2.054767
H	2.050452	8.045970	2.138208
H	2.979975	9.377574	1.370917
H	1.216719	9.533063	1.587662
C	3.719209	9.346124	4.033117
H	3.861990	9.752017	5.050461
H	4.547043	9.711519	3.399335
H	3.817862	8.246314	4.091019
C	-2.293052	11.038770	5.294954
H	-2.372788	11.566073	4.313293
C	-2.083658	12.132280	6.356642
H	-1.967215	11.689008	7.362838
H	-2.941298	12.829273	6.375059
H	-1.176638	12.730160	6.138115
C	-3.622802	10.301821	5.501970
H	-3.756712	9.491590	4.763395
H	-4.464100	11.008089	5.388689
H	-3.706085	9.859699	6.512042
Pd	1.958368	13.032459	2.544128
P	1.897801	15.263747	3.308880
C	3.179144	16.054042	4.366648
C	3.999598	15.241121	5.209034
C	4.959132	15.872449	6.021548
H	5.596111	15.263938	6.673867
C	5.118169	17.262284	6.012574
H	5.871684	17.733520	6.655943
C	4.316708	18.050194	5.178745
H	4.453142	19.137686	5.175511
C	3.341009	17.474654	4.344700
C	2.536598	18.374013	3.412272
H	1.544589	17.881816	3.259417
C	3.206402	18.457555	2.028809
H	4.212516	18.909852	2.106098
H	2.598665	19.065148	1.333920
H	3.315763	17.450171	1.580692
C	2.248609	19.771495	3.975285
H	1.831296	19.719869	4.996707
H	1.516150	20.290812	3.331803
H	3.153227	20.406463	4.011559
C	3.843298	13.726020	5.281645
H	3.443210	13.399087	4.291505
C	2.791602	13.327042	6.330704
H	3.103369	13.641906	7.343670
H	2.635990	12.232669	6.333856
H	1.813300	13.798346	6.110217
C	5.163419	12.972903	5.492311
H	5.929839	13.287851	4.762320
H	5.001688	11.887692	5.368000
H	5.576054	13.123463	6.506949
Pd	-0.037814	16.364457	2.522056
P	-1.933941	15.201143	3.303060
C	-3.242346	15.923244	4.376276
C	-2.923294	17.011968	5.244723
C	-3.934246	17.519573	6.081769
H	-3.704062	18.352724	6.755705
C	-5.226252	16.983561	6.069820
H	-5.997936	17.393883	6.733059
C	-5.533629	15.925358	5.206916
H	-6.550406	15.516048	5.200254
C	-4.563352	15.377015	4.348682
C	-4.961876	14.263027	3.386773
H	-4.045893	13.649666	3.205405
C	-5.373083	14.846407	2.024026
H	-6.252487	15.508392	2.129574
H	-5.622342	14.040592	1.309949
H	-4.547136	15.439947	1.583240
C	-6.031006	13.308698	3.933270
H	-5.768681	12.940930	4.941232
H	-6.130022	12.434933	3.264726
H	-7.027755	13.783483	3.994509
C	-1.524074	17.613036	5.324568
H	-1.041606	17.442308	4.332247
C	-0.660700	16.874863	6.361928
H	-1.094452	16.965197	7.374917
H	0.365957	17.284038	6.380798
H	-0.581390	15.795790	6.119020
C	-1.512843	19.129573	5.559579
H	-2.155992	19.656210	4.832284
H	-0.485400	19.518257	5.449403
H	-1.853973	19.400742	6.575717
Pd	1.925503	15.268307	0.947085
Pd	0.003624	14.103333	-0.654069
P	-0.031604	16.318377	0.161211
C	-0.044646	17.825233	-0.891746
C	1.079915	18.115110	-1.725022
C	1.039966	19.273153	-2.523055
H	1.892963	19.510342	-3.169217
C	-0.066755	20.129140	-2.507759
H	-0.076039	21.026388	-3.139139
C	-1.161371	19.839812	-1.685240
H	-2.022036	20.518781	-1.679667
C	-1.178495	18.695431	-0.867272
C	-2.374119	18.452971	0.047884
H	-2.440273	17.350131	0.212951
C	-2.143078	19.089261	1.430016
H	-2.049956	20.187987	1.346540
H	-2.979620	18.856544	2.113945
H	-1.216411	18.700774	1.897052
C	-3.719083	18.888109	-0.548186
H	-3.862060	18.482183	-1.565489
H	-4.546929	18.522926	0.085704
H	-3.817510	19.987937	-0.606143
C	2.292990	17.194613	-1.810126
H	2.372696	16.667505	-0.828358
C	2.083575	16.100887	-2.871587
H	1.967113	16.543950	-3.877872
H	2.941207	15.403880	-2.889879
H	1.176556	15.503064	-2.652910
C	3.622760	17.931492	-2.017272
H	3.756662	18.741893	-1.278882
H	4.464044	17.225236	-1.903801
H	3.706082	18.373379	-3.027443
Pd	-1.958425	15.201205	0.940672
P	-1.897862	12.969910	0.175917
C	-3.179205	12.179592	-0.881833
C	-3.999695	12.992476	-1.724223
C	-4.959256	12.361105	-2.536673
H	-5.596291	12.969586	-3.188965
C	-5.118294	10.971270	-2.527620
H	-5.871849	10.500001	-3.170918
C	-4.316795	10.183401	-1.693788
H	-4.453229	9.095909	-1.690491

C	-3.341060	10.758985	-0.859816
C	-2.536578	9.859694	0.072618
H	-1.544584	10.351940	0.225403
C	-3.206312	9.776176	1.456115
H	-4.212417	9.323850	1.378885
H	-2.598526	9.168617	2.150991
H	-3.315679	10.783569	1.904210
C	-2.248548	8.462200	-0.490356
H	-1.831306	8.513805	-1.511809
H	-1.516018	7.942949	0.153099
H	-3.153134	7.827183	-0.526547
C	-3.843478	14.507583	-1.796849
H	-3.443493	14.834564	-0.806685
C	-2.791710	14.906587	-2.845825
H	-3.103368	14.591672	-3.858809
H	-2.636157	16.000968	-2.849001
H	-1.813401	14.435340	-2.625239
C	-5.163621	15.260620	-2.007662
H	-5.930090	14.945663	-1.277727
H	-5.001957	16.345845	-1.883385
H	-5.576158	15.109992	-3.022330
Pd	0.037749	11.869229	0.962739
P	1.933874	13.032522	0.181742
C	3.242310	12.310306	-0.891351
C	2.923345	11.221322	-1.759508
C	3.934347	10.713560	-2.596395
H	3.704219	9.880257	-3.270161
C	5.226315	11.249667	-2.584571
H	5.998020	10.839253	-3.247729
C	5.533613	12.308110	-1.721935
H	6.550349	12.717522	-1.715397
C	4.563276	12.856639	-0.863884
C	4.961782	13.970750	0.097893
H	4.045717	14.583936	0.279433
C	5.373420	13.387522	1.460577
H	6.252917	12.725686	1.354871
H	5.622693	14.193420	2.174554
H	4.547675	12.793859	1.901586
C	6.030584	14.925279	-0.448894
H	5.767958	15.292924	-1.456823
H	6.129547	15.799109	0.219575
H	7.027428	14.450718	-0.510318
C	1.524111	10.620277	-1.839329
H	1.041524	10.791363	-0.847124
C	0.660892	11.358111	-2.877065
H	1.094761	11.267379	-3.889968
H	-0.365780	10.948973	-2.895900
H	0.581597	12.437279	-2.634567
C	1.512833	9.103661	-2.073819
H	2.155869	8.577239	-1.346268
H	0.485355	8.715070	-1.963629
H	1.854063	8.832131	-3.089827

188

Pd8(PDip)6 (PBE0)

Pd	-1.893712	12.975964	2.529106
Pd	-0.003304	14.128287	4.130390
P	0.036295	11.929162	3.308548
C	0.043089	10.404921	4.321488
C	-1.078966	10.093705	5.134062
C	-1.053513	8.916659	5.886630
H	-1.905550	8.665756	6.514920
C	0.038837	8.058239	5.846751
H	0.037488	7.145238	6.440748
C	1.132628	8.367103	5.047140
H	1.982859	7.688239	5.022639
C	1.162082	9.532558	4.276324
C	2.362002	9.792679	3.384577
H	2.429609	10.885705	3.243550
C	2.150039	9.180833	1.999549
H	2.071699	8.087551	2.064803
H	2.982565	9.433393	1.330812
H	1.227246	9.560278	1.538633
C	3.691087	9.349455	3.987424
H	3.816836	9.733848	5.006656
H	4.519902	9.725880	3.375548
H	3.788866	8.256946	4.024229
C	-2.279225	11.015045	5.245407
H	-2.365530	11.548418	4.281915
C	-2.050621	12.081809	6.316325
H	-1.965534	11.624363	7.310691
H	-2.879087	12.801226	6.330037
H	-1.126989	12.644181	6.121675
C	-3.603882	10.289391	5.456097
H	-3.746605	9.490707	4.718441
H	-4.433967	10.997930	5.352749
H	-3.681840	9.846633	6.457405
Pd	1.928571	13.050288	2.533896
P	1.879443	15.258249	3.294877
C	3.221950	16.037300	4.265910
C	4.088807	15.228120	5.047912
C	5.130618	15.844854	5.745235
H	5.804732	15.237532	6.345477
C	5.325370	17.220001	5.685101
H	6.144929	17.679695	6.235969
C	4.472841	18.006205	4.919951
H	4.632447	19.081858	4.877781
C	3.413608	17.442377	4.203259
C	2.548513	18.346835	3.345203
H	1.567598	17.850395	3.239084
C	3.135441	18.479906	1.939450
H	4.112760	18.979508	1.971179
H	2.466296	19.058942	1.290498
H	3.274188	17.493009	1.477036
C	2.274556	19.713674	3.964698
H	1.919369	19.620905	4.997912
H	1.504437	20.236442	3.384315
H	3.164376	20.356286	3.971318
C	3.894401	13.729311	5.177296
H	3.462554	13.385000	4.220413
C	2.872427	13.402497	6.266266
H	3.244079	13.709263	7.252650
H	2.661517	12.325753	6.290210
H	1.921781	13.921328	6.083317
C	5.186604	12.944815	5.376857
H	5.941168	13.214216	4.628506
H	4.985616	11.871448	5.282525
H	5.620866	13.104183	6.372262
Pd	-0.042521	16.337825	2.523090
P	-1.924126	15.183620	3.289932
C	-3.273026	15.926023	4.281381
C	-2.998829	17.036225	5.123320
C	-4.053560	17.613253	5.834769
H	-3.858305	18.466544	6.480707
C	-5.348784	17.118730	5.732764
H	-6.156688	17.585004	6.295182
C	-5.609182	16.026210	4.914813
H	-6.625037	15.642375	4.843485
C	-4.591439	15.409300	4.181828
C	-4.946001	14.245862	3.274712
H	-4.027853	13.646197	3.147641
C	-5.340375	14.741163	1.884173
H	-6.258316	15.342116	1.928103
H	-5.506110	13.896322	1.203560
H	-4.546264	15.365609	1.451527
C	-6.001020	13.306191	3.849727
H	-5.747414	12.995949	4.870480
H	-6.073301	12.406594	3.226270
H	-6.998805	13.762698	3.873918
C	-1.593135	17.575133	5.311145
H	-1.058239	17.412422	4.358851
C	-0.849211	16.770264	6.377324

H	-1.326700	16.888767	7.358738
H	0.195924	17.095178	6.454424
H	-0.846688	15.699078	6.130518
C	-1.525317	19.071183	5.595976
H	-2.082385	19.648534	4.848382
H	-0.480996	19.403481	5.570608
H	-1.919573	19.325182	6.588237
Pd	1.893704	15.257644	0.955703
Pd	0.003286	14.105288	-0.645544
P	-0.036327	16.304419	0.176283
C	-0.043125	17.828769	-0.836496
C	1.078867	18.140098	-1.649130
C	1.053387	19.317305	-2.401445
H	1.905364	19.568295	-3.029782
C	-0.038904	20.175787	-2.361246
H	-0.037581	21.088905	-2.955063
C	-1.132621	19.866810	-1.561579
H	-1.982826	20.545699	-1.536858
C	-1.162071	18.701162	-0.791055
C	-2.361887	18.440898	0.100790
H	-2.429564	17.347838	0.241519
C	-2.149644	19.052313	1.485963
H	-2.071148	20.145602	1.421016
H	-2.982112	18.799685	2.154745
H	-1.226843	18.672591	1.946632
C	-3.691027	18.884403	-0.501733
H	-3.816975	18.500282	-1.521043
H	-4.519779	18.507899	0.110180
H	-3.788710	19.976930	-0.538243
C	2.279083	17.218745	-1.760811
H	2.365606	16.685313	-0.797370
C	2.050214	16.152053	-2.831744
H	1.964943	16.609565	-3.826065
H	2.878649	15.432603	-2.845668
H	1.126601	15.589706	-2.636939
C	3.603701	17.944387	-1.971778
H	3.746620	18.743025	-1.234111
H	4.433798	17.235826	-1.868675
H	3.681422	18.387201	-2.973081
Pd	-1.928624	15.183299	0.950895
P	-1.879444	12.975312	0.189925
C	-3.221868	12.196281	-0.781238
C	-4.088575	13.005494	-1.563363
C	-5.130262	12.388800	-2.260907
H	-5.804273	12.996155	-2.861232
C	-5.325039	11.013653	-2.200860
H	-6.144505	10.553988	-2.751891
C	-4.472665	10.227413	-1.435572
H	-4.632301	9.151761	-1.393462
C	-3.413567	10.791202	-0.718652
C	-2.548635	9.886698	0.139519
H	-1.567689	10.383063	0.245725
C	-3.135724	9.753702	1.545212
H	-4.113069	9.254156	1.513385
H	-2.466683	9.174640	2.194250
H	-3.274469	10.740616	2.007590
C	-2.274722	8.519817	-0.479902
H	-1.919438	8.612518	-1.513089
H	-1.504693	7.997015	0.100569
H	-3.164589	7.877269	-0.486574
C	-3.894169	14.504318	-1.692587
H	-3.462503	14.848554	-0.735597
C	-2.871981	14.831232	-2.781324
H	-3.243411	14.524513	-3.767805
H	-2.661104	15.907986	-2.805157
H	-1.921357	14.312423	-2.598201
C	-5.186347	15.288811	-1.892330
H	-5.941052	15.019326	-1.144152
H	-4.985397	16.362174	-1.797858
H	-5.620414	15.129531	-2.887833
Pd	0.042499	11.895707	0.961729
P	1.924093	13.049975	0.194881
C	3.272925	12.307593	-0.796672
C	2.998739	11.197252	-1.638430
C	4.053458	10.620191	-2.349871
H	3.858205	9.766820	-2.995703
C	5.348664	11.114786	-2.248000
H	6.156551	10.648504	-2.810435
C	5.609067	12.207386	-1.430157
H	6.624908	12.591278	-1.358935
C	4.591330	12.824359	-0.697218
C	4.945922	13.987774	0.209920
H	4.027810	14.587505	0.336925
C	5.340160	13.492455	1.600492
H	6.258035	12.891396	1.556622
H	5.505958	14.337291	2.281095
H	4.545953	12.868113	2.033111
C	6.001052	14.927364	-0.365023
H	5.747538	15.237629	-1.385792
H	6.073363	15.826953	0.258444
H	6.998802	14.470779	-0.389147
C	1.593066	10.658229	-1.826094
H	1.058107	10.821377	-0.873906
C	0.849184	11.462553	-2.892717
H	1.326705	11.343536	-3.874054
H	-0.195955	11.137619	-2.969680
H	0.846671	12.533869	-2.646470
C	1.525304	9.162038	-2.110197
H	2.082361	8.585072	-1.362297
H	0.480993	8.829722	-2.084708
H	1.919609	8.907566	-3.102316

188

Pd8(PDip)6 (B3LYP)

Pd	-1.922374	12.963026	2.535953
Pd	-0.006756	14.126067	4.164916
P	0.031225	11.912777	3.316409
C	0.035385	10.377390	4.322965
C	-1.093634	10.058275	5.130835
C	-1.068042	8.875310	5.880817
H	-1.920220	8.618651	6.505633
C	0.029241	8.017115	5.841477
H	0.027295	7.101442	6.431650
C	1.128300	8.331682	5.044738
H	1.977365	7.652245	5.019996
C	1.159458	9.503818	4.277855
C	2.366921	9.769828	3.385372
H	2.438361	10.859793	3.246837
C	2.158307	9.159133	1.989831
H	2.097764	8.064214	2.049836
H	2.983801	9.429142	1.319040
H	1.228869	9.526482	1.535173
C	3.704284	9.327625	3.990691
H	3.832013	9.721002	5.006666
H	4.531112	9.701502	3.373674
H	3.802358	8.235287	4.036150
C	-2.304162	10.978763	5.238215
H	-2.389879	11.515803	4.280338
C	-2.089968	12.048702	6.320978
H	-2.011550	11.587629	7.314521
H	-2.923538	12.762662	6.328937
H	-1.169423	12.617487	6.135247
C	-3.636096	10.245692	5.434335
H	-3.771173	9.453771	4.687439
H	-4.465657	10.955376	5.330080
H	-3.720678	9.792449	6.430633
Pd	1.952204	13.032845	2.544934
P	1.891881	15.261051	3.308540
C	3.238225	16.039281	4.285744
C	4.111452	15.224585	5.063062
C	5.149504	15.842146	5.772425
H	5.825102	15.234853	6.370066

C	5.337017	17.222554	5.726401	H	-2.097008	20.169523	1.434989
H	6.152092	17.681422	6.284868	H	-2.983175	18.804759	2.165932
C	4.480917	18.013914	4.963515	H	-1.228290	18.707115	1.949554
H	4.637759	19.089762	4.932270	C	-3.704032	18.906369	-0.505600
C	3.423719	17.450511	4.236823	H	-3.831993	18.512995	-1.521547
C	2.551586	18.362011	3.380287	H	-4.530833	18.532655	0.111554
H	1.572072	17.869814	3.272349	H	-3.801913	19.998724	-0.551065
C	3.137117	18.505496	1.965517	C	2.304262	17.254535	-1.753441
H	4.108721	19.016508	1.996615	H	2.389950	16.717615	-0.795495
H	2.459534	19.077738	1.319245	C	2.090142	16.184454	-2.836076
H	3.284812	17.522182	1.500929	H	2.011716	16.645402	-3.829678
C	2.270642	19.732242	4.008498	H	2.923746	15.470532	-2.843938
H	1.906621	19.631441	5.038396	H	1.169622	15.615646	-2.650287
H	1.503043	20.256179	3.424857	C	3.636183	17.987627	-1.949585
H	3.159721	20.375964	4.026606	H	3.771193	18.779648	-1.202783
C	3.927717	13.715547	5.174820	H	4.465763	17.277986	-1.845198
H	3.493838	13.376345	4.220679	H	3.720801	18.440749	-2.945935
C	2.911672	13.361679	6.272605	Pd	-1.952136	15.200709	0.939928
H	3.290156	13.654599	7.260946	P	-1.891827	12.972502	0.176346
H	2.709870	12.282731	6.279597	C	-3.238253	12.194246	-0.800728
H	1.956257	13.875104	6.105189	C	-4.111502	13.008911	-1.578059
C	5.232703	12.931163	5.351979	C	-5.149768	12.391353	-2.287112
H	5.975996	13.214107	4.597020	H	-5.825418	12.998628	-2.884712
H	5.034221	11.857906	5.245969	C	-5.337500	11.010987	-2.240738
H	5.679355	13.081343	6.343575	H	-6.152768	10.552127	-2.798930
Pd	-0.040244	16.365311	2.533407	C	-4.481401	10.219664	-1.477815
P	-1.936986	15.193923	3.298434	H	-4.638420	9.143848	-1.446281
C	-3.288231	15.948390	4.287877	C	-3.423977	10.783061	-0.751448
C	-3.015224	17.077953	5.112141	C	-2.551840	9.871603	0.105127
C	-4.069658	17.651718	5.833926	H	-1.572167	10.363570	0.212626
H	-3.877475	18.513793	6.468072	C	-3.136985	9.728739	1.520116
C	-5.363933	17.140518	5.754836	H	-4.108771	9.218044	1.489473
H	-6.169253	17.604023	6.323515	H	-2.459396	9.156461	2.166350
C	-5.624922	16.035101	4.947944	H	-3.284204	10.712242	1.984455
H	-6.638469	15.644224	4.892393	C	-2.271458	8.501097	-0.522737
C	-4.607622	15.417879	4.208138	H	-1.907672	8.601478	-1.552759
C	-4.963503	14.238374	3.310116	H	-1.503863	7.977110	0.060865
H	-4.048749	13.639266	3.181863	H	-3.160733	7.857634	-0.540425
C	-5.372139	14.719110	1.908965	C	-3.927663	14.517919	-1.690041
H	-6.298669	15.307102	1.952062	H	-3.493618	14.857210	-0.736006
H	-5.528582	13.865831	1.236977	C	-2.911758	14.871564	-2.788028
H	-4.589161	15.349296	1.467559	H	-3.290428	14.578567	-3.776274
C	-6.013992	13.291289	3.902046	H	-2.709855	15.950492	-2.795175
H	-5.745938	12.984652	4.920612	H	-1.956364	14.358070	-2.620709
H	-6.087698	12.389230	3.281431	C	-5.232613	15.302382	-1.867115
H	-7.013594	13.743552	3.937017	H	-5.975822	15.019594	-1.112016
C	-1.609777	17.648416	5.266388	H	-5.034031	16.375637	-1.761273
H	-1.085288	17.476265	4.313270	H	-5.679414	15.152109	-2.858630
C	-0.823618	16.887504	6.346428	Pd	0.040304	11.868272	0.951464
H	-1.283057	17.027990	7.333840	P	1.937048	13.039661	0.186419
H	0.215347	17.238709	6.389050	C	3.288225	12.285195	-0.803121
H	-0.799883	15.810789	6.131979	C	3.015108	11.155760	-1.627523
C	-1.563918	19.161270	5.508760	C	4.069484	10.581993	-2.349391
H	-2.143572	19.705434	4.753037	H	3.877200	9.720072	-2.983714
H	-0.525489	19.509604	5.455547	C	5.363792	11.093108	-2.270309
H	-1.948184	19.437961	6.499115	H	6.169047	10.629640	-2.839110
Pd	1.922437	15.270546	0.948926	C	5.624881	12.198435	-1.463330
Pd	0.006811	14.107486	-0.680039	H	6.638449	12.589262	-1.407802
P	-0.031162	16.320767	0.168442	C	4.607653	12.815633	-0.723403
C	-0.035286	17.856044	-0.838277	C	4.963662	13.995043	0.174694
C	1.093707	18.175008	-1.646235	H	4.048951	14.594189	0.303060
C	1.068130	19.357877	-2.396370	C	5.372381	13.514171	1.575774
H	1.920312	19.614443	-3.021220	H	6.298881	12.926141	1.532560
C	-0.029094	20.216146	-2.357058	H	5.528912	14.367388	2.247820
H	-0.027119	21.131760	-2.947321	H	4.589408	12.883983	2.017187
C	-1.128105	19.901762	-1.560177	C	6.014165	14.942112	-0.417236
H	-1.977109	20.581274	-1.535429	H	5.746057	15.248843	-1.435759
C	-1.159280	18.729724	-0.793148	H	6.087969	15.844119	0.203444
C	-2.366655	18.463936	0.099520	H	7.013738	14.489793	-0.452312
H	-2.438263	17.373987	0.238088	C	1.609561	10.585616	-1.782032
C	-2.157728	19.074617	1.495022	H	1.084915	10.757945	-0.829028

C	0.823807	11.346634	-2.862296
H	1.283423	11.205968	-3.849600
H	-0.215233	10.995674	-2.905113
H	0.800282	12.423372	-2.647936
C	1.563405	9.072758	-2.024320
H	2.142770	8.528506	-1.268439
H	0.524884	8.724670	-1.971306
H	1.947815	8.795918	-3.014576

264

Pd8(PDip)6(XylNC)4 (HOpt)

Pd	10.028164	13.523327	8.346104
Pd	9.255918	11.941891	4.541874
Pd	11.945201	9.960162	7.098547
Pd	12.976867	13.672050	5.364849
Pd	9.474956	11.102840	7.143018
Pd	10.297445	14.190096	5.717132
Pd	12.546652	12.537713	7.816708
Pd	11.894242	11.257573	4.667013
P	8.461324	13.049205	6.554730
P	10.934075	11.326485	8.888069
P	10.215981	9.869359	5.351661
P	11.896293	14.671990	7.298850
P	11.162361	13.250623	3.803353
P	13.633774	11.413462	6.142252
N	9.219112	15.027169	11.043694
N	6.825887	12.256327	2.555896
N	15.223943	15.653391	4.330697
C	9.429588	14.500962	10.026874
C	9.033881	15.638874	12.290547
C	8.046876	15.121713	13.134957
C	7.896392	15.726428	14.367301
H	7.135252	15.350586	15.050296
C	8.711814	16.786830	14.757641
H	8.581996	17.239627	15.739201
C	9.682215	17.271942	13.891430
H	10.317628	18.103291	14.194569
C	9.868844	16.707437	12.634896
C	10.886832	17.245978	11.679131
H	11.413472	16.453774	11.134984
H	11.628976	17.858074	12.199728
H	10.409979	17.873630	10.916473
C	7.197612	13.953649	12.720378
H	6.626771	14.181760	11.812681
H	6.495023	13.684946	13.514304
H	7.819561	13.078681	12.492148
C	7.716377	12.023251	3.251257
C	5.831550	12.605118	1.647424
C	4.548414	12.110635	1.862161
C	3.586860	12.451493	0.895596
H	2.571707	12.075869	1.022679
C	3.897976	13.235357	-0.188867
H	3.127721	13.478028	-0.919621
C	5.176175	13.733151	-0.345500
H	5.419940	14.366236	-1.197738
C	6.178484	13.419927	0.565402
C	7.576916	13.929904	0.406418
H	8.305419	13.112479	0.359566
H	7.874028	14.554049	1.259378
H	7.677930	14.528429	-0.503037
C	4.235064	11.272902	3.055572
H	4.812102	10.339551	3.046813
H	3.171609	11.018374	3.088412
H	4.494453	11.793981	3.986846
C	12.568733	8.067745	7.476223
N	12.884550	6.960905	7.488424
C	13.219860	5.609329	7.449775
C	14.586313	5.374435	7.167238
C	15.019878	4.053849	7.077801
C	14.129611	3.006643	7.297558
C	12.805757	3.253123	7.621279
C	12.350380	4.571525	7.645333
C	10.932449	4.925858	7.942377
C	15.545671	6.501257	6.857939
C	14.402628	14.884507	4.623149
C	16.123236	16.712345	4.127893
C	17.316115	16.444901	3.436077
C	18.152040	17.545309	3.230819
H	19.086930	17.384161	2.693225
C	17.836720	18.790804	3.678653
H	18.515687	19.623070	3.498868
C	16.652842	19.007552	4.372893
H	16.407156	20.003210	4.739889
C	15.764185	17.965658	4.604758
C	14.511521	18.171256	5.400648
H	14.275593	19.235263	5.493606
H	14.614937	17.761115	6.414697
H	13.655860	17.660583	4.950081
C	17.655341	15.072844	2.956770
H	16.908680	14.707862	2.239481
H	17.665640	14.353999	3.785853
H	18.636091	15.058405	2.472505
C	6.621905	13.269177	6.599242
C	5.811394	12.397085	7.378352
C	4.412580	12.517374	7.244417
H	3.773608	11.850468	7.817378
C	3.830246	13.458693	6.435484
H	2.744193	13.519429	6.366801
C	4.617387	14.330877	5.712049
H	4.150394	15.082176	5.079923
C	6.012923	14.254340	5.775769
C	6.821441	15.287181	5.006355
H	7.754474	14.783617	4.708042
C	6.166227	15.777430	3.742166
H	6.868534	16.394799	3.170182
H	5.289990	16.408871	3.942467
H	5.840878	14.952940	3.097008
C	7.186952	16.398672	5.914584
H	7.822984	17.134722	5.407310
H	7.738997	16.027662	6.785160
H	6.295119	16.924426	6.284690
C	6.340203	11.427027	8.385790
H	7.242650	10.950422	7.959185
C	6.774771	12.146386	9.657207
H	7.496070	12.938252	9.438531
H	7.253686	11.449778	10.356757
H	5.902450	12.595117	10.150932
C	5.357968	10.299286	8.787081
H	4.529262	10.688002	9.392140
H	5.890205	9.568406	9.406266
H	4.935490	9.773548	7.924307
C	10.622938	10.941996	10.664054
C	9.807106	9.822850	11.000065
C	9.244936	9.770502	12.270873
H	8.603705	8.929855	12.530339
C	9.495208	10.736050	13.212924
H	9.024636	10.684222	14.194104
C	10.416944	11.726171	12.937492
H	10.678813	12.432739	13.721110
C	11.009101	11.849076	11.688496
C	12.127117	12.843744	11.493073
H	11.960114	13.360377	10.537161
C	12.219178	13.899624	12.587577
H	12.957823	14.658475	12.305072
H	12.551885	13.468391	13.541074
H	11.268069	14.411051	12.761828
C	13.428534	12.095747	11.396894
H	14.267460	12.779192	11.229214
H	13.408752	11.383524	10.565795
H	13.625359	11.531510	12.318725
C	9.804054	8.560236	10.116239
H	9.889304	8.873573	9.067244

C	8.565764	7.720757	10.277709
H	8.573307	6.888103	9.564140
H	7.653780	8.301812	10.095493
H	8.489970	7.277806	11.279027
C	11.067639	7.763244	10.484904
H	11.155247	6.868433	9.863146
H	11.031484	7.456065	11.538284
H	11.968865	8.367147	10.333956
C	9.625199	8.270111	4.617598
C	8.392798	7.728680	5.035740
C	7.925126	6.559529	4.413150
H	6.966874	6.147685	4.722659
C	8.639936	5.923924	3.423235
H	8.253952	5.018639	2.956342
C	9.844640	6.456942	3.019605
H	10.404711	5.949167	2.238816
C	10.381202	7.621655	3.591387
C	11.774714	8.050660	3.146693
H	11.811599	9.152745	3.177042
C	12.107959	7.599726	1.720766
H	11.331399	7.877686	0.999565
H	12.255510	6.514156	1.663328
H	13.051237	8.058736	1.405488
C	12.801090	7.523140	4.108438
H	12.730612	6.430923	4.201513
H	12.667432	7.964238	5.097494
H	13.816430	7.770733	3.776768
C	7.468190	8.381208	6.052173
H	8.067739	9.068031	6.668352
C	6.839414	7.337579	6.983906
H	6.083622	6.730518	6.471158
H	6.336471	7.830310	7.822356
H	7.597147	6.660542	7.394221
C	6.419736	9.183694	5.320448
H	6.886245	9.954917	4.697526
H	5.740954	9.693029	6.013819
H	5.817179	8.534073	4.671148
C	12.523715	16.239031	8.065134
C	13.743244	16.255856	8.821224
C	14.127904	17.439723	9.431661
H	15.048074	17.457204	10.010629
C	13.392065	18.601542	9.329646
H	13.732121	19.509806	9.826088
C	12.238043	18.601360	8.595384
H	11.657758	19.518890	8.514399
C	11.766191	17.444832	7.951003
C	10.464565	17.596184	7.199721
H	10.267628	16.674985	6.632780
C	10.499597	18.773032	6.226507
H	9.567868	18.814519	5.649871
H	11.333784	18.692088	5.520635
H	10.596048	19.733015	6.747453
C	9.309329	17.813367	8.199047
H	8.369874	18.010463	7.673516
H	9.518081	18.676611	8.843919
H	9.167665	16.933186	8.835149
C	14.657907	15.064169	8.966917
H	14.050606	14.147329	8.890945
C	15.409157	15.041708	10.303830
H	15.923779	14.083009	10.420634
H	14.738580	15.178942	11.159671
H	16.181886	15.818884	10.350078
C	15.694880	15.037557	7.824691
H	15.202364	14.922475	6.857938
H	16.381988	14.193235	7.947526
H	16.281143	15.965951	7.816388
C	11.239337	13.906261	2.076651
C	11.231780	15.306666	1.837050
C	11.268875	15.770899	0.522398
H	11.272581	16.845143	0.347823
C	11.276223	14.920714	-0.557508
H	11.281625	15.312512	-1.573818
C	11.278075	13.561210	-0.330014
H	11.293736	12.879402	-1.178895
C	11.304034	13.020533	0.967718
C	11.401438	11.517315	1.079039
H	11.611038	11.265121	2.127514
C	12.559428	10.983114	0.220122
H	12.362674	11.105181	-0.851636
H	12.703689	9.914274	0.402531
H	13.496012	11.503805	0.449989
C	10.088275	10.831232	0.688449
H	9.803512	11.077011	-0.343340
H	9.277271	11.135647	1.358117
H	10.183370	9.742004	0.764267
C	11.144901	16.358342	2.938718
H	11.473439	15.890040	3.876022
C	12.039927	17.559882	2.697182
H	13.082389	17.260006	2.536146
H	12.008435	18.230994	3.562408
H	11.725273	18.156587	1.831659
C	9.690301	16.762494	3.109843
H	9.285242	17.181079	2.178510
H	9.581161	17.516365	3.897121
H	9.082829	15.894598	3.388188
C	15.322876	10.669250	6.015541
C	16.060351	10.408724	7.202315
C	17.264354	9.689719	7.101242
H	17.829461	9.484942	8.009043
C	17.728492	9.240965	5.902980
H	18.661017	8.678260	5.854353
C	17.023953	9.491239	4.752541
H	17.411650	9.132235	3.802493
C	15.828792	10.213117	4.768878
C	15.162449	10.541535	3.448982
H	14.071255	10.493808	3.605184
C	15.508840	11.971224	3.020727
H	14.960742	12.251823	2.112581
H	16.585211	12.054729	2.820317
H	15.242073	12.690514	3.799239
C	15.524122	9.574621	2.294207
H	14.880582	9.782098	1.433481
H	15.393027	8.523367	2.574758
H	16.561196	9.713818	1.964351
C	15.649934	10.818369	8.606406
H	14.789896	11.499311	8.529036
C	16.768558	11.552317	9.350386
H	17.618487	10.893070	9.565228
H	16.394314	11.918882	10.313223
H	17.145623	12.406862	8.777817
C	15.211190	9.598813	9.424436
H	14.312190	9.141608	8.997243
H	14.988900	9.884845	10.458570
H	16.006516	8.842240	9.452893
H	10.625093	5.845224	7.432649
H	10.780624	5.074975	9.018937
H	10.249832	4.127084	7.630902
H	15.236790	7.049738	5.958558
H	16.556180	6.116761	6.697295
H	15.579421	7.239808	7.666781
H	16.059242	3.847840	6.828381
H	14.486071	1.978778	7.234627
H	12.103973	2.440353	7.796896

32

Pd8(PDip)6(XylNC)4 (HOpt) trunc

Pd	9.972223	13.551995	8.259768
Pd	9.285756	11.802210	4.513237
Pd	11.956273	9.966187	7.195296
Pd	12.969375	13.615928	5.324152
Pd	9.471054	11.075916	7.150679
Pd	10.277837	14.111671	5.609873

Pd	12.511946	12.579523	7.814141
Pd	11.930182	11.159794	4.710773
P	8.442553	12.981921	6.464238
P	10.897062	11.392347	8.908697
P	10.258540	9.778709	5.425376
P	11.842997	14.681164	7.196163
P	11.187478	13.104747	3.751944
P	13.642034	11.401058	6.206753
N	9.097710	15.156332	10.877863
N	6.886227	12.000001	2.475798
N	15.208014	15.582684	4.244848
C	9.332357	14.590917	9.887731
C	7.767619	11.808427	3.195012
C	12.597672	8.099935	7.662604
N	12.927525	6.998988	7.726636
C	14.391826	14.815592	4.555773
H	6.122846	12.170677	1.856637
H	15.902516	16.240832	3.962003
H	8.893115	15.646202	11.722918
H	13.211915	6.045017	7.791080
H	12.275581	15.951316	7.685247
H	7.061805	13.345757	6.495852
H	9.850284	8.484168	4.984741
H	15.007765	10.996309	6.127124
H	11.259013	13.553521	2.398537
H	10.808938	10.980334	10.272232

264

Pd8(PDip)6(XylNC)4 (r^2SCAN-3c)

Pd	-1.826225	-7.008355	-2.259655
Pd	-0.228746	-5.824756	-0.318142
P	-0.079777	-8.111465	-1.214598
C	-0.117322	-9.469147	0.051870
C	-1.293705	-9.711013	0.813007
C	-1.249504	-10.642681	1.852946
H	-2.140645	-10.810003	2.449384
C	-0.103954	-11.362676	2.137170
H	-0.095683	-12.084261	2.949830
C	1.029761	-11.163913	1.366689
H	1.920227	-11.745075	1.580147
C	1.054496	-10.229560	0.331585
C	2.321849	-10.134917	-0.496773
H	2.404078	-9.101430	-0.867255
C	2.206518	-11.068005	-1.709351
H	2.120228	-12.108798	-1.372937
H	3.095376	-10.983270	-2.344946
H	1.330946	-10.826293	-2.316094
C	3.601234	-10.466924	0.280808
H	3.630319	-9.974079	1.257376
H	4.474475	-10.144475	-0.295073
H	3.707103	-11.545636	0.442664
C	-2.625987	-9.012642	0.593719
H	-2.606928	-8.543399	-0.400472
C	-2.840865	-7.907397	1.633654
H	-2.876239	-8.334963	2.643506
H	-3.783827	-7.379601	1.453158
H	-2.035671	-7.170101	1.596911
C	-3.803756	-9.996983	0.618071
H	-3.622141	-10.865872	-0.021625
H	-4.710006	-9.493869	0.271760
H	-4.011819	-10.361711	1.629595
Pd	1.656569	-6.838118	-2.085409
P	1.686609	-4.630388	-1.336964
C	2.773280	-3.704584	-0.153890
C	3.735745	-4.407198	0.613377
C	4.343857	-3.765559	1.695982
H	5.044513	-4.315469	2.316134
C	4.086532	-2.438376	1.982165
H	4.567139	-1.956793	2.829621
C	3.232726	-1.715739	1.160806
H	3.078066	-0.662755	1.365526
C	2.566205	-2.314522	0.092726
C	1.724654	-1.437114	-0.817646
H	0.821520	-2.010486	-1.090615
C	2.496357	-1.138540	-2.113413
H	3.383776	-0.534589	-1.886571
H	1.866613	-0.581516	-2.818293
H	2.817899	-2.058212	-2.609220
C	1.274564	-0.118850	-0.182075
H	0.783018	-0.268771	0.784654
H	0.570466	0.387213	-0.848487
H	2.117379	0.566243	-0.035757
C	4.196110	-5.821520	0.301284
H	3.891129	-6.051761	-0.729697
C	3.544408	-6.855257	1.222709
H	3.744516	-6.620053	2.275402
H	3.947869	-7.849858	1.010012
H	2.462578	-6.884802	1.068162
C	5.727249	-5.932474	0.376337
H	6.216051	-5.134069	-0.191161
H	6.056767	-6.895327	-0.025837
H	6.086998	-5.882817	1.409120
Pd	-0.270055	-3.893728	-2.318983
P	-2.204788	-4.869627	-1.472063
C	-3.654179	-4.240104	-0.499727
C	-3.553329	-3.013320	0.214585
C	-4.643588	-2.567786	0.963193
H	-4.563428	-1.638910	1.517506
C	-5.827307	-3.281414	1.013459
H	-6.665908	-2.914475	1.599242
C	-5.934055	-4.468688	0.310298
H	-6.866899	-5.019509	0.353422
C	-4.873885	-4.977034	-0.443185
C	-5.130682	-6.273265	-1.197361
H	-4.167623	-6.795517	-1.314695
C	-5.673200	-5.982833	-2.601549
H	-6.628695	-5.446739	-2.538039
H	-5.837886	-6.917206	-3.149804
H	-4.969082	-5.383847	-3.179440
C	-6.099957	-7.218514	-0.477234
H	-5.845444	-7.357125	0.577878
H	-6.083922	-8.193503	-0.971123
H	-7.133395	-6.859051	-0.533137
C	-2.293428	-2.165798	0.258474
H	-1.733285	-2.340661	-0.671984
C	-1.410216	-2.602896	1.433229
H	-1.922780	-2.399086	2.381771
H	-0.458920	-2.058428	1.428573
H	-1.185445	-3.670452	1.383418
C	-2.577436	-0.661809	0.361260
H	-3.350778	-0.340972	-0.344545
H	-1.663981	-0.098343	0.149726
H	-2.901138	-0.378673	1.368891
Pd	1.979999	-4.656170	-3.781295
Pd	-0.046550	-6.033327	-5.094578
P	-0.162260	-3.761962	-4.629605
C	0.061710	-2.203922	-5.618115
C	0.951541	-2.175370	-6.729994
C	1.425590	-0.947195	-7.182860
H	2.135842	-0.915248	-8.001725
C	0.998286	0.246723	-6.612612
H	1.398428	1.193869	-6.964588
C	0.013501	0.219407	-5.642870
H	-0.387546	1.154182	-5.260975
C	-0.488025	-0.989814	-5.150855
C	-1.669499	-0.917627	-4.203835
H	-1.986366	-1.937973	-3.966179
C	-1.314986	-0.231046	-2.888625
H	-0.916322	0.777709	-3.048328
H	-2.200856	-0.146246	-2.253708
H	-0.564289	-0.819458	-2.352632
C	-2.848737	-0.218898	-4.897871

H	-3.095027	-0.712649	-5.843758
H	-3.737672	-0.244856	-4.258912
H	-2.626407	0.831735	-5.113895
C	1.262366	-3.432730	-7.516795
H	1.556695	-4.211764	-6.802131
C	-0.034623	-3.880546	-8.214106
H	-0.423910	-3.066193	-8.836945
H	0.154738	-4.745062	-8.856570
H	-0.800629	-4.150790	-7.480734
C	2.371071	-3.286660	-8.557337
H	3.298976	-2.895847	-8.130316
H	2.587331	-4.266914	-8.994900
H	2.066665	-2.627453	-9.378957
Pd	-2.295280	-4.847241	-3.968042
P	-1.939726	-7.254002	-4.551237
C	-3.048026	-8.294473	-5.609217
C	-3.110029	-8.075487	-7.015153
C	-3.847329	-8.957002	-7.805648
H	-3.897000	-8.798357	-8.877285
C	-4.523712	-10.033355	-7.253753
H	-5.083154	-10.713375	-7.890836
C	-4.492475	-10.225946	-5.884507
H	-5.035450	-11.062663	-5.455905
C	-3.780547	-9.371607	-5.039912
C	-3.837292	-9.682970	-3.552283
H	-3.478993	-8.799523	-3.004517
C	-5.264441	-9.974562	-3.070931
H	-5.643242	-10.927345	-3.456089
H	-5.270603	-10.051197	-1.979297
H	-5.964323	-9.188425	-3.370955
C	-2.917071	-10.856094	-3.197893
H	-1.884374	-10.648672	-3.488150
H	-2.928578	-11.038005	-2.118069
H	-3.250455	-11.772223	-3.701465
C	-2.491819	-6.865153	-7.694667
H	-1.590274	-6.576973	-7.130995
C	-2.075341	-7.110382	-9.147982
H	-2.941815	-7.209852	-9.811082
H	-1.500269	-6.253115	-9.512150
H	-1.460146	-8.010199	-9.253639
C	-3.482063	-5.693358	-7.630738
H	-3.744839	-5.456570	-6.596525
H	-3.048145	-4.795619	-8.086487
H	-4.399171	-5.950069	-8.175544
Pd	0.061026	-8.451737	-3.697447
P	1.922816	-7.042475	-4.386917
C	3.301316	-7.790332	-5.380789
C	3.052521	-8.389466	-6.646871
C	4.112999	-8.973436	-7.341170
H	3.926224	-9.433672	-8.305822
C	5.399733	-8.982956	-6.825828
H	6.208742	-9.448668	-7.382476
C	5.645028	-8.394409	-5.596904
H	6.652608	-8.405533	-5.192931
C	4.622777	-7.791908	-4.861680
C	4.973948	-7.247257	-3.490327
H	4.202480	-6.527079	-3.200556
C	4.933891	-8.409643	-2.490833
H	5.704660	-9.152516	-2.731787
H	5.099853	-8.057175	-1.468430
H	3.958143	-8.902386	-2.526578
C	6.312329	-6.506700	-3.430845
H	6.367490	-5.717264	-4.187653
H	6.420447	-6.034010	-2.450091
H	7.171244	-7.173479	-3.564976
C	1.700048	-8.343471	-7.331355
H	0.922845	-8.354964	-6.559129
C	1.581356	-7.019240	-8.092900
H	2.353726	-6.956131	-8.870062
H	0.601157	-6.942976	-8.571846
H	1.699508	-6.169717	-7.414493
C	1.429563	-9.510953	-8.286432
H	1.693540	-10.477486	-7.849163
H	0.363382	-9.535334	-8.536731
H	1.976559	-9.403840	-9.230401
C	0.087496	-5.832961	1.643749
N	0.542852	-5.874965	2.730672
C	1.243561	-5.908870	3.910728
C	1.615175	-4.689133	4.503187
C	2.357947	-4.746988	5.679228
C	2.707018	-5.969348	6.244632
C	2.314225	-7.160799	5.643057
C	1.572317	-7.158901	4.464503
H	3.286762	-5.993824	7.162663
H	2.586959	-8.112322	6.091552
H	2.663152	-3.819095	6.155477
C	1.137967	-8.424009	3.791317
H	0.046736	-8.474960	3.699385
H	1.476739	-9.300300	4.348483
H	1.530934	-8.493781	2.770399
C	1.210726	-3.394174	3.870317
H	0.119284	-3.292344	3.852364
H	1.551809	-3.337877	2.830683
H	1.630637	-2.545429	4.415255
C	-3.854527	-3.789235	-4.585157
N	-4.762251	-3.077878	-4.834873
C	-5.802921	-2.187652	-4.937049
C	-6.372740	-1.959935	-6.200907
C	-7.415576	-1.041520	-6.282146
C	-7.870090	-0.376267	-5.148228
C	-6.240826	-1.528507	-3.772827
C	-5.855699	-2.691579	-7.402748
H	-6.376439	-2.370975	-8.307855
H	-5.986144	-3.774245	-7.291522
H	-4.781681	-2.519734	-7.538174
C	-5.595646	-1.807111	-2.450861
H	-5.983965	-1.142138	-1.676451
H	-4.506732	-1.686919	-2.498374
H	-5.770181	-2.840185	-2.127898
H	-7.872815	-0.845585	-7.248223
C	-7.286307	-0.618649	-3.909125
H	-8.683405	0.338493	-5.231151
H	-7.643481	-0.093111	-3.027570
C	0.345671	-10.205522	-4.575676
N	0.630141	-11.137873	-5.239710
C	0.855740	-12.176090	-6.108498
C	-0.198814	-12.586494	-6.944747
C	0.061877	-13.614176	-7.846746
C	1.320028	-14.204096	-7.915777
C	2.346100	-13.772843	-7.081321
C	2.138290	-12.749590	-6.160139
C	3.227070	-12.254506	-5.257405
H	2.987423	-12.463924	-4.208626
H	3.355807	-11.168688	-5.344340
H	4.178147	-12.735774	-5.496706
C	-1.531943	-11.911457	-6.862276
H	-2.226888	-12.322138	-7.597764
H	-1.449574	-10.830722	-7.033656
H	-1.975754	-12.024223	-5.867100
H	3.328729	-14.232334	-7.145126
H	-0.733449	-13.949253	-8.507019
H	1.503541	-15.002574	-8.628585
C	3.519273	-3.786150	-4.668170
N	4.327540	-3.328710	-5.394268
C	5.200917	-2.767087	-6.290963
C	5.778745	-3.588435	-7.276918
C	5.449354	-1.385411	-6.204901
C	6.315998	-0.830250	-7.142409
C	6.907109	-1.618865	-8.124449
C	6.637782	-2.982182	-8.189769
H	7.094264	-3.590222	-8.966080
H	6.525718	0.235176	-7.100829

H	7.579200	-1.166450	-8.847584
C	5.448720	-5.048256	-7.339936
H	4.365057	-5.212045	-7.356928
H	5.823495	-5.585540	-6.461992
H	5.883192	-5.509827	-8.229487
C	4.779399	-0.566081	-5.144255
H	3.687908	-0.616453	-5.241374
H	5.086663	0.480246	-5.208979
H	5.022846	-0.940490	-4.143303

34

PPh3 (r²SCAN-3c)

H	-2.237976	-2.397561	1.364988
C	-2.864707	-1.603710	0.939963
C	-4.203169	-1.482711	1.340451
H	-4.618492	-2.180149	2.080461
C	-5.027925	-0.472478	0.798263
C	-4.478300	0.427952	-0.136872
H	-5.107133	1.218760	-0.563415
C	-3.133393	0.314692	-0.525777
H	-2.717508	1.023040	-1.253267
C	-2.324879	-0.701791	0.007099
H	-1.275327	-0.789114	-0.299498
C	-7.581176	-1.665239	0.283381
C	-8.908113	-2.038145	0.590624
H	-9.408335	-1.588343	1.458943
C	-9.589394	-2.972961	-0.202231
H	-10.622025	-3.248707	0.045273
C	-8.945623	-3.564420	-1.302479
H	-9.473851	-4.303056	-1.917784
C	-7.621640	-3.210067	-1.607064
H	-7.113112	-3.669196	-2.464131
C	-6.942763	-2.263505	-0.822259
H	-5.911520	-1.984307	-1.070145
H	-6.643727	2.309702	2.324217
C	-7.141245	2.360207	1.346244
C	-7.542667	3.600505	0.828851
H	-7.353925	4.517206	1.401274
C	-8.196618	3.666154	-0.413164
H	-8.519923	4.634446	-0.814530
C	-8.440360	2.486513	-1.134656
H	-8.952756	2.531209	-2.103884
C	-8.026849	1.245266	-0.624103
H	-8.213561	0.327817	-1.195399
C	-7.369124	1.170402	0.620782
P	-6.783568	-0.410985	1.392445

9. References

[1] A. Schumann, F. Reiss, H. Jiao, J. Rabeah, J. E. Siewert, I. Krummenacher, H. Braunschweig, C. Hering-Junghans, *Chem. Sci.* **2019**, *10*, 7859-7867.

[2] a) A. Grünwald, N. Orth, A. Scheurer, F. W. Heinemann, A. Pöthig, D. Munz, *Angew. Chem. Int. Ed.* **2018**, *57*, 16228-16232; *Angew. Chem.* **2018**, *130*, 16463-16467; b) A. Grünwald, F. W. Heinemann, D. Munz, *Angew. Chem. Int. Ed.* **2020**, *59*, 21088-21095; *Angew. Chem.* **2020**, *132*, 21274-21281; c) A. Grünwald, B. Goswami, K. Breitwieser, B. Morgenstern, M. Gimferrer, F. W. Heinemann, D. M. Momper, C. W. M. Kay, D. Munz, *J. Am. Chem. Soc.* **2022**, *144*, 8897-8901.

[3] D. Fenske, H. Schottmüller, *Z. Anorg. Allg. Chem.* **1998**, *624*, 443-451.

[4] G. M. Sheldrick, *Acta Crystallogr. A: Found. Adv.* **2015**, *71*, 3-8.

[5] O. V. Dolomanov, L. J. Bourhis, R. J. Gildea, J. A. K. Howard, H. Puschmann, *J. Appl. Cryst.* **2009**, *42*, 339-341.

[6] C. B. Hübschle, G. M. Sheldrick, B. Dittrich, *J. Appl. Cryst.* **2011**, *44*, 1281-1284.

[7] L. Krause, R. Herbst-Irmer, G. M. Sheldrick, D. Stalke, *J. Appl. Crystallogr.* **2015**, *48*, 3-10.

[8] K. Brandenburg, in *DIAMOND*, V. 4.6.7 ed., **2022**.

[9] D. A. Shirley, *Phys. Rev. B: Condens. Matter* **1972**, *5*, 4709-4714.

[10] J. J. Yeh, I. Lindau, *At. Data Nucl. Data Tables* **1985**, *32*, 1-155.

[11] NIST X-ray Photoelectron Spectroscopy Database, NIST Standard Reference Database Number 20, National Institute of Standards and Technology, Gaithersburg MD, 20899 (2000), DOI: https://dx.doi.org/10.18434/T4T88K, (accessed: 20.11.2023).

[12] a) F. Neese, *WIREs Comput. Mol. Sci.* **2012**, *2*, 73-78; b) F. Neese, *WIREs Comput. Mol. Sci.* **2022**, *12*, e1606.

[13] S. Grimme, A. Hansen, S. Ehlert, J.-M. Mewes, *J. Chem. Phys.* **2021**, *154*, 064103.

[14] J. P. Perdew, K. Burke, M. Ernzerhof, *Phys. Rev. Lett.* **1996**, *77*, 3865-3868.

[15] a) H. Kruse, S. Grimme, *J. Chem. Phys.* **2012**, *136*, 154101; b) C. Adamo, V. Barone, *J. Chem. Phys.* **1999**, *110*, 6158-6170.

[16] C. van Wüllen, *J. Chem. Phys.* **1998**, *109*, 392-399.

[17] a) F. Weigend, R. Ahlrichs, *Phys. Chem. Chem. Phys.* **2005**, *7*, 3297-3305; b) F. Weigend, *Phys. Chem. Chem. Phys.* **2006**, *8*, 1057-1065.

[18] a) S. Grimme, J. Antony, S. Ehrlich, H. Krieg, *J. Chem. Phys.* **2010**, *132*, 154104; b) S. Grimme, S. Ehrlich, L. Goerigk, *J. Comput. Chem.* **2011**, *32*, 1456-1465.

[19] A. D. Becke, *J. Chem. Phys.* **1993**, *98*, 5648-5652.

[20] G. Knizia, *J. Chem. Theory Comput.* **2013**, *9*, 4834-4843.

[21] V. N. Staroverov, G. E. Scuseria, J. Tao, J. P. Perdew, *J. Chem. Phys.* **2003**, *119*, 12129-12137.

[22] S. Hirata, M. Head-Gordon, *Chem. Phys. Lett.* **1999**, *314*, 291-299.

[23] a) R. Ditchfield, *Mol. Phys.* **1974**, *27*, 789-807; b) K. Wolinski, J. F. Hinton, P. Pulay, *J. Am. Chem. Soc.* **2002**, *112*, 8251-8260.

[24] D. Van Allen, D. Venkataraman, *J. Org. Chem.* **2003**, *68*, 4590-4593.

[25] a) A. D. Becke, K. E. Edgecombe, *J. Chem. Phys.* **1990**, *92*, 5397-5403; b) T. Lu, F.-W. Chen, *Acta Phys.-Chim. Sin.* **2011**, *27*, 2786-2792; A. Savin, R. Nesper, S. Wengert, T. F. Fässler *Angew. Chem. Int. Ed.* **1997**, *36*, 1808-1832; *Angew. Chem.* **1997**, *109*, 1892-1918.

[26] T. Lu, F. Chen, *J. Comput. Chem.* **2012**, *33*, 580-592.

10. Author Contributions.

Kevin Breitwieser (Investigation, Lead, equal; Writing – Review and Editing; Formal Analysis – Supporting), Matteo Bevilacqua (Investigation, Lead, equal; Writing – Review and Editing), Sneha Mullassery (Investigation, Supporting), Samuel Grandthyll (Investigation, Supporting), Frank Müller (Investigation, Supporting), Fabian Dankert (Investigation, Supporting), Bernd Morgenstern (Investigation, Supporting), Andrea Biffis (Supervision, Supporting), Christian Hering-Junghans (Conceptualization, Lead, equal; Writing – Review and Editing; Formal Analysis – Supporting, Validation – Supporting), Dominik Munz (Conceptualization – Lead, equal; Writing – Original Draft; Writing – Review and Editing; Funding Acquisition – Lead; Formal Analysis – Lead; Project Administration – Lead; Supervision – Lead; Validation – Lead).
